# Disentangling interaction and bias effects in opinion dynamics of large language models

**DOI:** 10.1038/s41467-026-77340-3

**Published:** 2026-09-22

**Authors:** Vincent C. Brockers, David A. Ehrlich, Viola Priesemann

**Affiliations:** 1 Max Planck Institute for Dynamics and Self-Organization, Am Faßberg 17, 37077 Göttingen, Germany; 2 Institute for the Dynamics of Complex Systems, Faculty of Physics, University of Göttingen, Friedrich-Hund-Platz 1, 37077 Göttingen, Germany; 3 Göttingen Campus Institute for Dynamics of Biological Networks, University of Göttingen, Heinrich-Düker-Weg 12, 37073 Göttingen, Germany

**Keywords:** Social sciences, Mathematics and computing, Physics

## Abstract

Large Language Models are increasingly used to simulate human opinion dynamics, yet the effect of genuine interaction is often obscured by systematic biases. We develop a Bayesian framework to disentangle and quantify three such biases: (i) A topic bias toward the LLM’s default stance; (ii) an agreement bias favoring agreement to the prompted statement irrespective of the question; and (iii) an anchoring bias toward the initiating agent’s stance. We apply this framework to various LLMs that performed multi-step dialogs on 12 different questions from climate change and societal justice to music preferences. We find that opinion trajectories tend to quickly converge to a shared attractor, with the influence of both interaction and biases decaying over time, and with the impact of biases differing between LLMs. In addition, we show that fine-tuning an LLM on different sets of strongly opinionated statements (including misinformation) shifts the opinion attractor correspondingly. By exposing stark differences between LLMs and providing quantitative tools for comparing interaction and bias contributions to opinion shifts in LLM agent discussions, our approach highlights both promises and pitfalls of using LLMs as proxies for human behavior.

## Abstract (web summary)

Here, the authors introduce an interpretable Bayesian framework for inferring effective opinion update rules among interacting large language models, separating interaction-driven opinion change from model-intrinsic biases.

## Introduction

Large Language Models (LLMs) demonstrate impressive capabilities in mimicking human behavior, generating text that is increasingly indistinguishable from human-produced language^1–3^. Because of their ability to accurately portray syntax, semantics and contextual relationships in language, LLMs have recently been adopted as powerful tools for agent-based simulations^4–6^, especially of social processes such as opinion dynamics^7–12^.

Beyond simulation studies, direct interactions between LLM agents are rapidly becoming a feature of deployed systems: autonomous agents now coordinate within multi-agent software frameworks^13,14^, negotiate contracts with one another in commercial legal workflows^15^, converse and form opinion-laden discourse communities on agent-only social platforms such as Moltbook^16^, and debate to improve factuality and enable scalable oversight^17–19^. In all of these settings, whether an agent’s response is driven by its interlocutor’s arguments or by model-intrinsic biases directly affects outcomes, underscoring the need for quantitative tools to disentangle these factors.

Traditional agent-based models for opinion dynamics typically represent opinions as discrete states, continuous variables or higher dimensional vectors, with explicit mathematical update rules governing the interaction between agents^20–23^. While often highly interpretable in nature, these simplified models fail to capture the rich, nuanced, and context-sensitive nature of real human communication^24,25^. In contrast, LLMs directly utilize natural language, enabling more detailed and context-sensitive interactions^26,27^. However, this greater realism comes at the cost of reduced interpretability. Since the factors driving opinion change are encoded in the LLM’s training weights rather than in explicit rules, they are less transparent.

Recent studies have shown that the factors behind LLM opinion dynamics include certain biases—systematic tendencies inherited from their training process—that can drive conversation outcomes away from the outcomes otherwise expected from human discussants^28–31^. Particularly, LLMs have been shown to exhibit what we term a *topic bias*, which makes them converge towards consensus reflecting opinions about the discussion topic instilled by their training process^7^. Additionally, an acquiescence or *agreement bias* has previously been observed for some LLMs, which makes agents more likely to answer ”agree” to a question irrespective of its content^32^. Furthermore, LLMs have in some instances been observed to express an *anchoring bias*^33^, making them overvalue the first opinion expressed in a discussion. While some of these biases can be related to human biases, others are attributed only to LLM-specific behavior, potentially masking the genuine interaction of different agents. This confusion motivates the need for a systematic framework to disentangle how much and how each of these factors contributes to the overall opinion dynamics observed in discussions between LLM agents.

A framework that allows us to quantify how the individual factors contribute to the overall opinion shifts can be found in Bayesian modeling. Bayesian models have in previous work been used to model opinion changes and decision making in human subjects^34–36^, allowing the effect and relevance of different factors to be captured in intuitive terms and providing tools to compare between models. On a broader level, Bayesian inference has been proposed as a core computational principle of human cognition, providing a principled mechanism for updating beliefs under uncertainty^37^. These properties make Bayesian modeling a suitable framework for constructing and evaluating factors of the influence-response function of LLM agents, that describes mathematically how an agent shifts their belief after one round of discussing with another agent.

In this work, we introduce a Bayesian framework for modeling how the observed opinion shifts in discussions between LLM agents are influenced by the interaction with the interlocutor and by different bias effects. We find that 1) opinion changes quickly converge after only one or two time steps up to residual variability by noise, 2) most LLMs are dominated by the topic bias which is largely in agreement with the LLM’s prior opinion when prompted alone, followed in relevance by the interaction effects, and 3) the anchoring and agreement biases typically contribute less and vary substantially in size depending on the LLM.

Furthermore, we propose to quantify the opinion of an LLM agent on a two-dimensional scale, with the expectation value of the LLM’s response distribution to a query representing its stance on a topic, and the Shannon entropy quantifying its uncertainty in this position. This uncertainty is found to be predictive of the variance of the subsequent opinion shift.

To address the issue of biases dominating the opinion dynamics in LLMs, we further explored the effectiveness of fine-tuning as a means to reinforce the persistence of the prompted initial opinion of an agent, shifting the dynamics towards the intended initialization. We demonstrate that fine-tuning LLMs to align with specific initial opinions shifts their bias towards the fine-tuned opinion and shows a trend towards a stronger influence of interaction in the overall dynamics.

By explicitly modeling the factors governing LLM interactions, our work offers human-interpretable metrics suitable for quantifying bias strength between different LLMs or influence of fine-tuning. The flexible nature of the Bayesian framework facilitates the construction of models including different influence factors, and provides rigorous methods for cross-study comparison. In future work, the Bayesian models may allow quantitative comparisons between LLM and human-generated discussions, and provide computationally efficient proxies for simulation of artificial agents in large social networks.

## Results

**Fig. 1:**
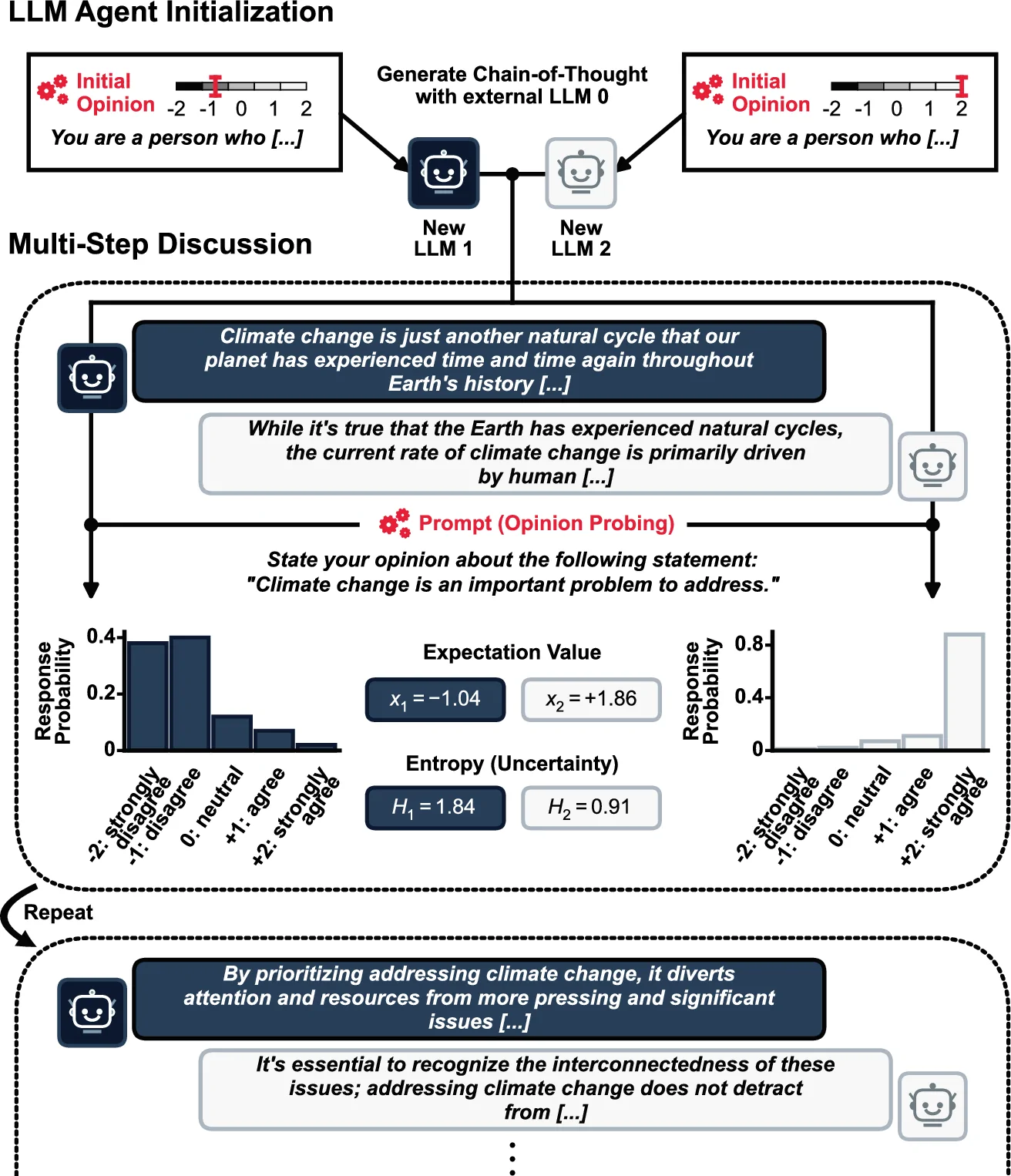
Two LLM agents discuss for multiple rounds about a topic (e.g., climate change), while their intermediate opinions are measured. Flowchart of an example simulation. First, both agents are initialized with an opinion on a five-point scale from  − 2 (strongly disagree) to 2 (strongly agree). This is done by prompting each agent with an externally generated Chain-of-Thought monologue. After that, the agents exchange arguments for five rounds back and forth about the given topic. Possible opinion changes are monitored by taking the expected value of directly asking the agents about their opinion. This query process is not saved in the agents conversation memory.

To disentangle interaction and bias effects in LLM opinion dynamics, we simulated dyadic multi-step discussions between two LLM agents (see Fig. 1 and Section 4.1). Each agent is first initialized to one of five opinions on a five-point scale from  − 2 (strongly disagree) to  + 2 (strongly agree) via an externally generated Chain-of-Thought monologue, and the two agents then exchange arguments for five rounds on one of twelve topic statements spanning four categories, from societal issues with scientific consensus to matters of personal preference (see Table 1). After every round, we probe each agent’s opinion on the topic statement in normal and negated framing and record the expectation value of its response distribution as the agent’s opinion ${x}_{i}^{(k)}(t)$, and the Shannon entropy of this distribution as the agent’s opinion uncertainty ${H}_{i}^{(k)}(t)$. We repeated this procedure for six LLMs: the four open-weight, locally hosted models Llama, Qwen, Mixtral, and its ”anti-aligned” variant DolphinMixtral, and the two cloud-hosted models GPT and Grok (see Section 4.1 and Supplementary Table 4 for model details and benchmarks).

**Table 1:** Twelve discussion topic statements from four topic categories and their negated framing used for initializing and measuring opinions in LLM discussions

Topic Category	Statements (negated)
Societal issues with scientific consensus	1. Climate change is (not) an important issue to address.
	2. Vaccinations are (not) necessary for public health.
	3. The earth is (not) flat.
Controversial societal issues	4. The global distribution of wealth is (not) fair.
	5. Abortions should (not) be legal.
	6. Social media does (not) have a positive impact on society.
Philosophical questions	7. Artificial intelligence is (not) dangerous.
	8. You can(not) only be a moral person if you believe in God.
	9. Humans do (not) possess free will.
Personal Preference	10. Bach is (not) a greater composer than Stravinsky.
	11. Pizza is (not) better than sushi.
	12. Modern art is (not) more meaningful than classical art.

Agreement to the statements is given or measured on a five-point scale.

In the following, we begin with a qualitative analysis of the observed discussion dynamics. We then fit our hierarchical Bayesian influence-response model (see Section 4.3) to these data to quantitatively compare the six LLMs, revealing pronounced differences in the relative importance of interaction and biases, and examine in a fine-tuning case study whether the inferred topic bias tracks the LLM’s prior opinion. Finally, we investigate how the entropy of the LLM’s response distribution captures opinion uncertainty.

### Convergence and biases in LLM opinion dynamics

**Fig. 2:**
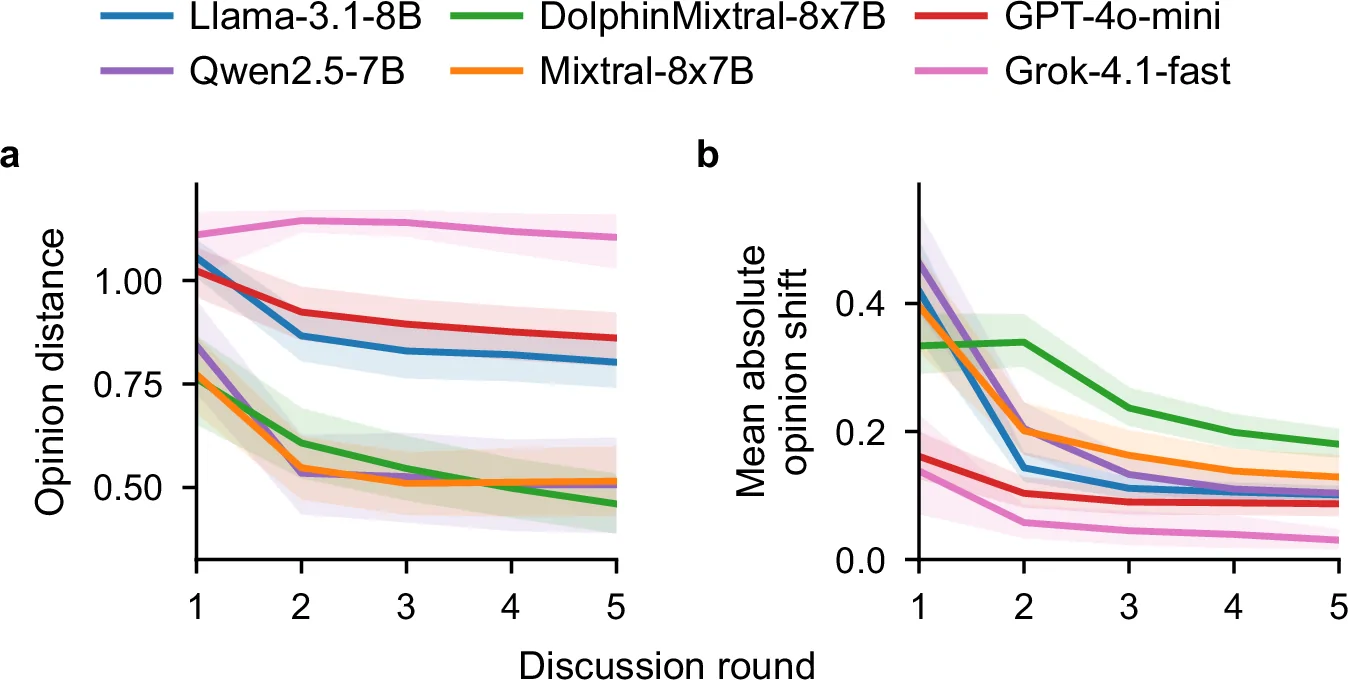
Opinions get closer over time for most LLMs, while opinion-shift magnitude decays rapidly over discussion rounds. **a** Opinion distance at time *t* divided by the opinion distance at time *t* = 0 decreases over time except for Grok, where it remains mostly constant. **b** Opinion-shift magnitude approaches a low plateau after the first exchanges. Smaller or less capable LLMs (Llama, Qwen, Mixtral and DolphinMixtral) appear to have an overall higher magnitude in opinion shift than bigger or more capable LLMs (GPT-4o and Grok). Both subplots show results per LLM across all simulations, all twelve discussion topics, and both agents. Error bands indicate the bootstrapped 95% CI of the observed data.

**Fig. 3:**
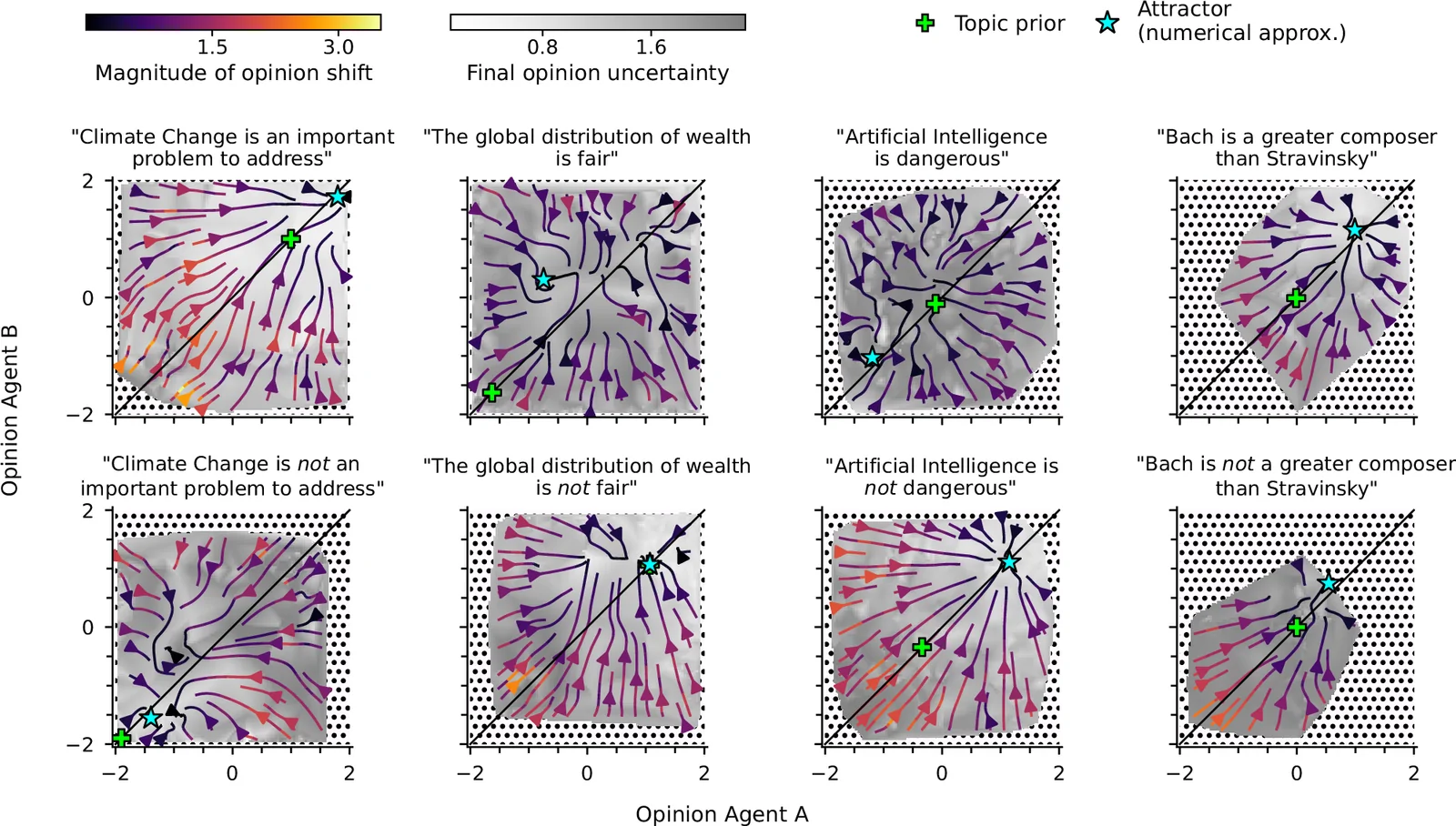
The opinion expectation values of both LLM agents tend to converge toward topic-dependent attractors in the opinion space. For DolphinMixtral, some of these attractors are biased toward positive opinions irrespective of framing, indicating an agreement bias. Grid interpolation of 25 simulation runs with the DolphinMixtral LLM for every possible combination of initial opinions on four example topics, with normal and negated framing (for all twelve topics, refer to Supplementary Fig. 21). The background shade indicates the average final entropy of the response distribution. The green plus symbolizes the LLM's prior opinion, i.e., the expected opinion of an LLM agent without prior opinion initialization. The cyan star represents a numerical approximation of the attractor position, based on the point with negative divergence and the lowest vector magnitude. The lack of data in the outer regions (dotted) indicates a failure to initialize agents with these extreme opinions.

For all LLMs except for Grok, the opinion distance of the two agents becomes smaller over the course of the discussion (see Fig. 2a). Across all LLMs, the magnitude of opinion shifts decays quickly after only a few rounds of discussions (see Fig. 2b), settling to a low but non-zero plateau. Moreover, an analysis of the opinion trajectories reveals that the opinions of two LLM agents typically converge toward an attractor point in opinion space for each of the discussion topics (see Fig. 3). This convergence usually occurs already after the first discussion round, with larger opinion shifts for agents further from the attractor and only marginal changes in subsequent discussion steps. Notably, the LLMs occasionally failed to adhere to the initialized opinions even before the discussion started (visible as the outer dotted regions in Fig. 3, e.g., for the question about musical preference).

In many instances, the observed attractor aligns closely with the topic prior, i.e., with the opinion when queried without prior initialization prompts, which reflects the LLM agent’s *topic bias* (see, for instance, the ”climate change” topic for DolphinMixtral in Fig. 3). For other questions, however (such as the question about musical preference in Fig. 3), the agents converge to significantly more positive values than anticipated from the topic prior alone. This *agreement bias* is especially apparent when considering the same statements in negated framing for opinion extraction: Here, instead of inverting their replies as expected, the agents exhibit a pronounced bias towards positive answers. Signatures of an *anchoring bias* (which would move the attractor off-diagonal in Fig. 3) are only weakly expressed for some questions (such as the question on global wealth distribution in Fig. 3).

### Bias and uncertainty differences between LLMs

Comparison across LLMs reveals that some patterns, such as rapid convergence and the presence of topic bias, are consistent. However, the agreement bias is present only in some LLMs, whereas the anchoring bias appears to have a slight negative influence for some topics when using other models than DolphinMixtral (see Supplementary Fig. 22).

Another notable difference concerns neutral opinions: For Llama, Qwen and DolphinMixtral, neutral average opinions primarily arise from a diffuse response distribution spreading probability across multiple answers, whereas Mixtral, GPT and Grok yield genuinely neutral responses, indicating considerably lower uncertainty, as discussed in Section 2.5.

### Quantifying influence factors via Bayesian modeling

To quantify the influences of the observed factors on opinion shifts and to compare the different LLMs systematically, we fit the Bayesian influence-response function outlined in Section 4.3 to the opinion dynamics of the generated discussions.

In general, the Bayesian analysis supports the qualitative findings described in the previous subsection. The effect sizes, however, vary markedly between models, with the more capable GPT and Grok (see Supplementary Table 4) showing the smallest opinion shifts overall.

**Fig. 4:**
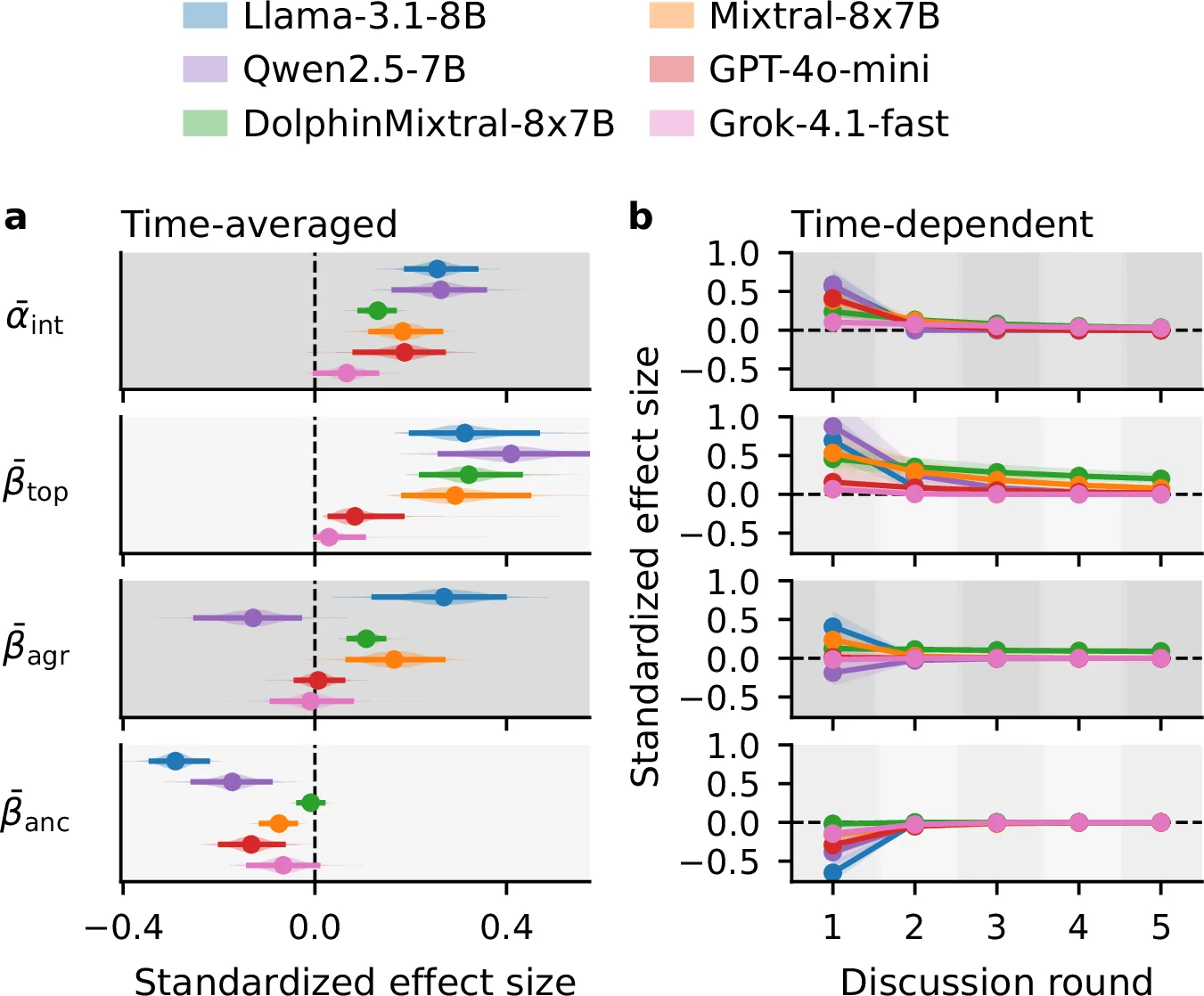
Standardized effect sizes of interaction and the three biases differ markedly across six LLMs. **a** Posterior distributions of the standardized hyperparameter effect sizes for interaction (${\bar{\alpha }}_{{{{\rm{interact}}}}}$) and topic, agreement, and anchoring bias (${\bar{\beta }}_{\bullet }$), pooled across all twelve topics, both agents, and five discussion steps (definition in Eq. (6)). Dots indicate posterior median and bars the 95% highest-density intervals. **b** Time-dependent standardized effect sizes across the five discussion rounds (see Eq. (7)); lines show posterior medians and shaded regions the 95% highest-density intervals. Positive values indicate attraction toward the respective predictor. See Supplementary Fig. 2 for the complementary leave-one-out ablation.

**Fig. 5:**
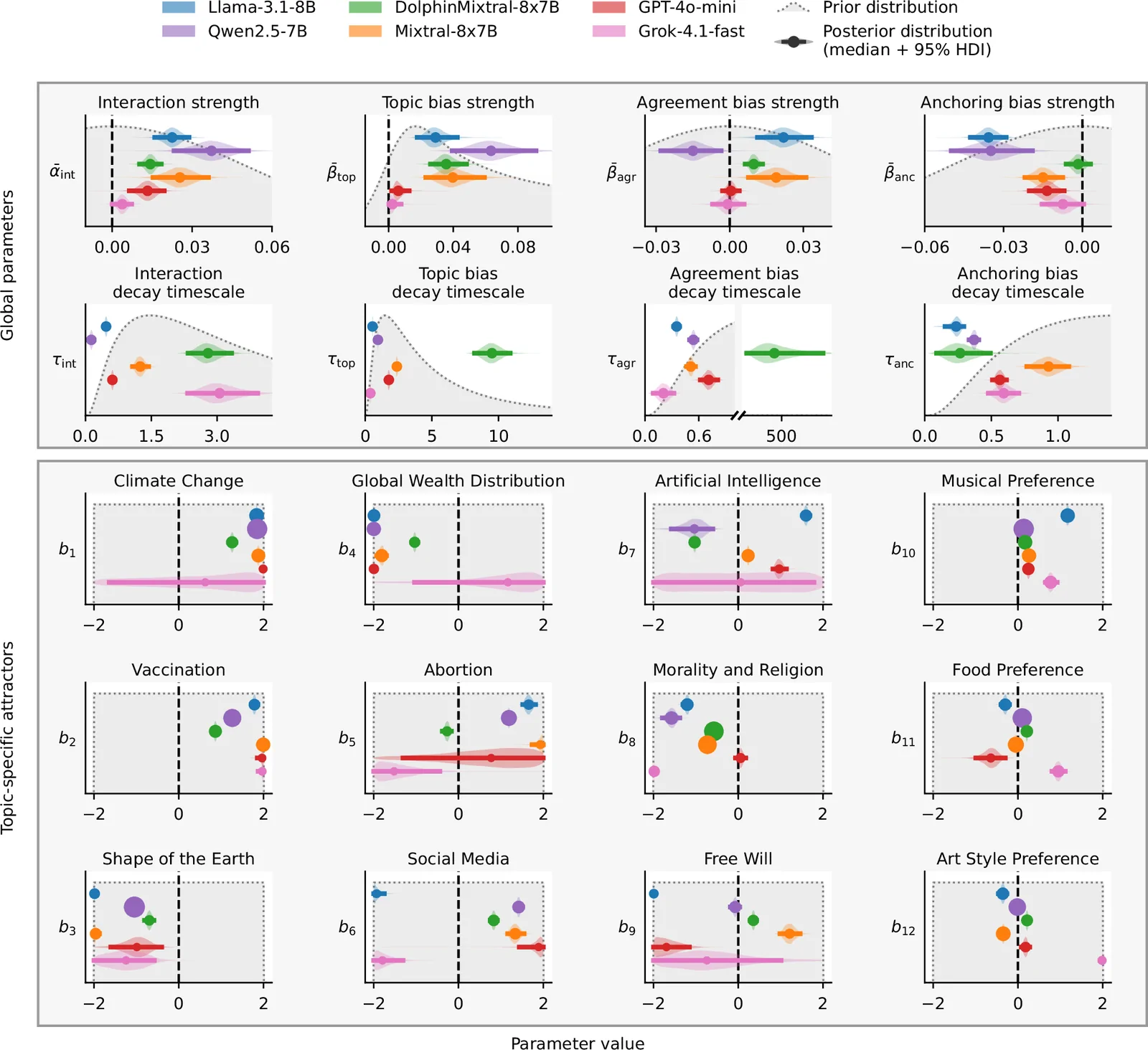
Inferred posterior distributions (median + 95% HDI) of all model parameters for the six different LLMs. The hierarchical effect coefficients ${\bar{\alpha }}_{{{{\rm{interact}}}}}$, ${\bar{\beta }}_{{{{\rm{agree}}}}}$ and ${\bar{\beta }}_{{{{\rm{anchor}}}}}$ have normally distributed priors (gray background). The hierarchical topic effect coefficient ${\bar{\beta }}_{{{{\rm{topic}}}}}$ is skewed towards positive values as the direction of attraction is accounted for by the individual topic bias attractors *b*_topic_, which are drawn uniformly from the opinion range [ − 2, 2]. The timescales *τ*_•_ follow a positively constrained heavy-tailed distribution. For exact prior and posterior values, refer to Supplementary Table 1 and Supplementary Table 5. For GPT and Grok, some topic bias attractors are weakly constrained due to having a topic-level effect strength *β*_top,*k*_ fitted around zero (see Supplementary Fig. 9). To indicate the general influence of an individual topic bias attractor *b*_*k*_, we scaled the corresponding dot size linearly with *β*_top,*k*_.

Specifically, the topic bias generally appears to be the most important contribution to the observed opinion shifts, yielding the largest effect size (see Fig. 4). This dominance is further supported by model ablation studies, where the topic bias alone shows the strongest explanatory power across all models (see Supplementary Fig. 2). The topic-specific attractors vary depending on the subject of the question: for societal issues with scientific consensus, the attractors are clustered towards the strong opinion representing the consensus, while being closer to zero for matters of personal preference, with the other subject categories falling in between. Generally, these attractors align qualitatively with the LLMs topic priors (compare Fig. 3 and Fig. 5, also see Supplementary Fig. 13).

The interaction effect has the second highest individual explanatory power for most models (see Supplementary Fig. 2) and even tends to surpass the effect strength of topic bias for the two more capable LLMs GPT and Grok.

The agreement bias reveals stark differences between the models: While for Llama, the agreement bias rivals topic bias and interaction in effect size, it takes on a negative value for Qwen. For the Mixtral models, the agreement bias is moderately positive, while it is close to zero for GPT and Grok. In the ablation study, the agreement bias alone is second only to the topic bias in individual explained variance for Llama (see Supplementary Fig. 2), while its contributions are lower for other models.

Interestingly, all models except for DolphinMixtral show a negative anchoring bias, meaning the opinion shifts have a tendency to drift away from the initially stated opinion. Despite apparently large effect sizes, however, the anchoring bias alone has very little predictive power across all models.

**Fig. 6:**
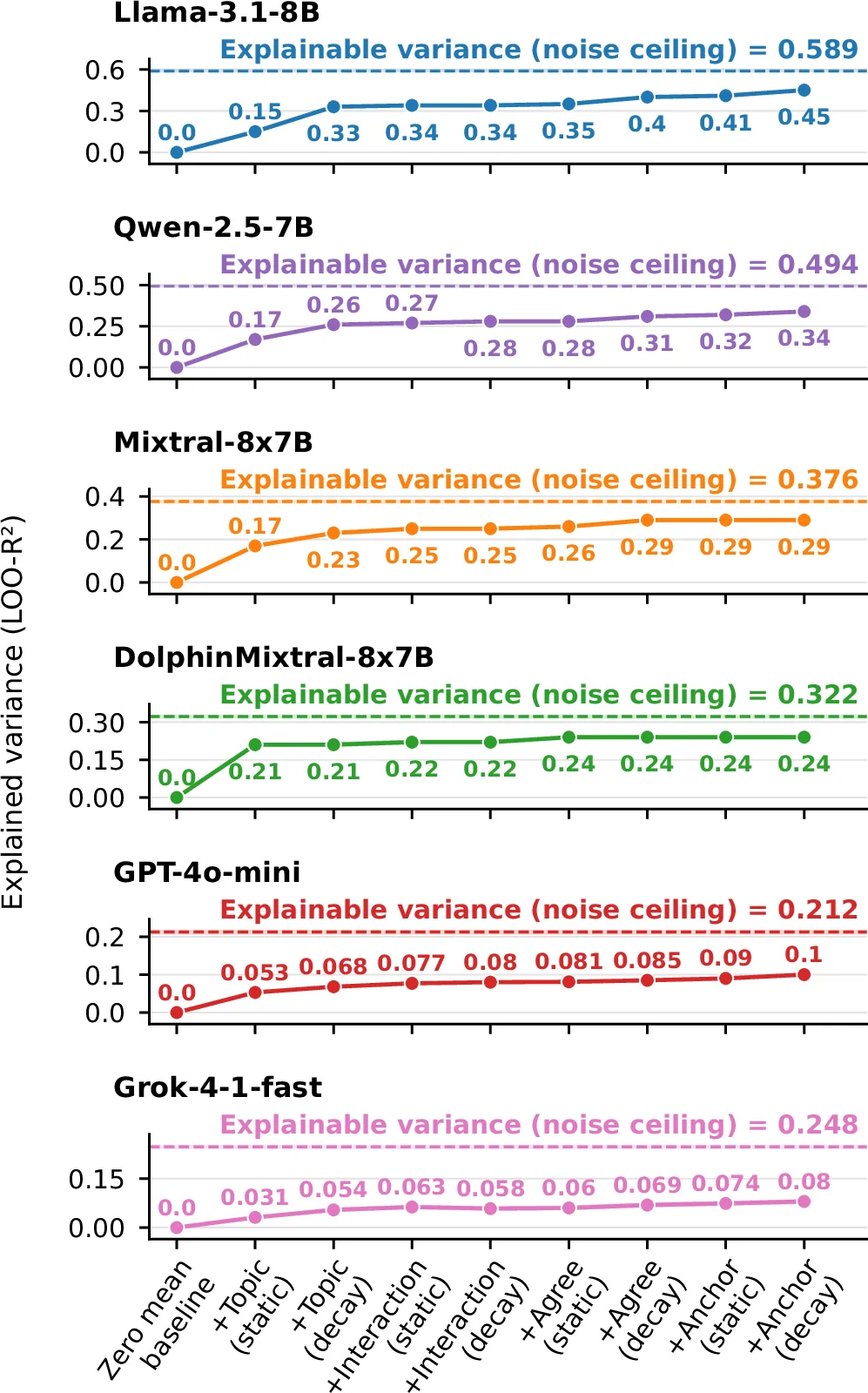
A Bayesian model with decaying topic bias captures the dominant structure of opinion shifts across LLMs, while adding interaction, agreement, and anchoring terms improves the fit and helps distinguish characteristic differences between LLMs. Shown is the leave-one-out explained variance (LOO-*R*^2^) for six LLMs as model terms are added incrementally, from a zero-mean baseline to the full model. Shaded bands around the LOO-*R*^2^ trajectories indicate 95% equal-tailed intervals. Dashed horizontal lines indicate the explainable variance (noise ceiling) for each model, and the corresponding translucent horizontal bands show its 95% confidence interval, allowing comparison between achieved and maximally explainable performance (see Supplementary Eq. 5). The impact of individual terms is shown in Supplementary Fig. 2.

Consistent with the interpretation above, an incremental model comparison shows that the topic-bias term captures the dominant structure, while adding interaction, agreement, and anchoring terms improves the fit, helping to distinguish LLM specific differences (see Fig. 6).

Furthermore, the estimated time scales *τ* modulating the exponential decay of the effects reveal that all effects decay quickly over only a few discussion steps, with agreement and anchoring bias decaying fastest. One notable exception to this rule is DolphinMixtral, where the agreement and topic biases appear significantly more persistent over time (see Fig. 5).

Finally, the standard deviation of the opinion shifts is largely accounted for by a constant baseline (see Supplementary Fig. 6), with a term proportional to the entropy of the output distribution used as a measure for uncertainty also contributing significantly (see Supplementary Fig. 7).

### Improving opinion persistence through fine-tuning

In the previous subsection it was shown that particularly the less capable LLMs were mostly driven by the topic bias, with interactions between the agents playing only a secondary role. Furthermore, this interaction effect decays fast with time, with only negligible changes after a few conversation steps. In order to improve the adherence to the initialized opinion and provide additional evidence that the topic bias tracks the LLMs prior opinion, we conducted a case study in which we augmented the opinion initialization process of the Mixtral LLM by fine-tuning it on statements expressing different levels of agreement to the climate change topic.

**Fig. 7:**
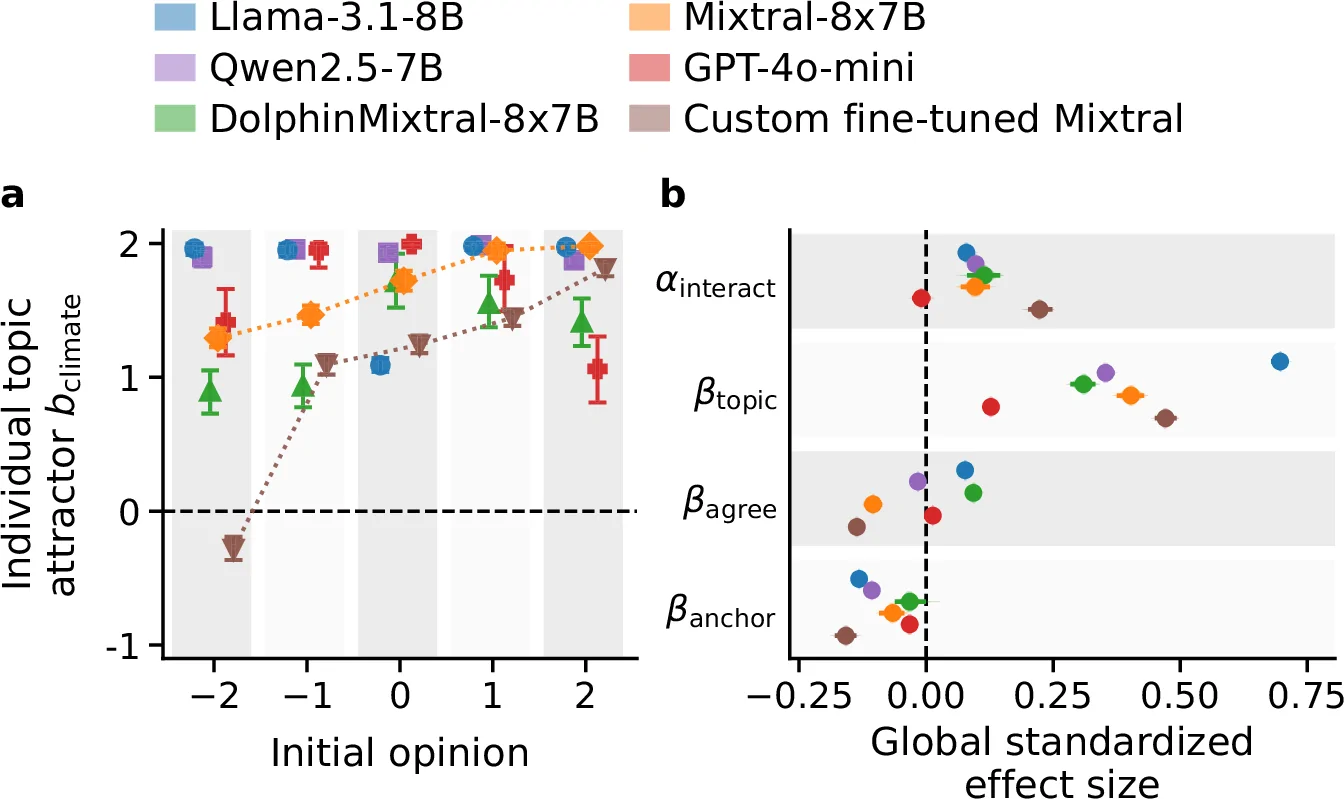
Fine-tuning strengthens the dependence of individual topic attractors on initial opinion. **a** The individual topic attractors, i.e., the attractors for a specific initialized opinion, of the custom fine-tuned Mixtral (brown) increase monotonically with the fine-tuned and initialized opinions. This trend is significantly more pronounced than in its base version (orange), particularly for the ''strongly disagree” opinion (-2) far from scientific consensus. Median values with 95% HDI. **b** For the Bayesian model with individual topic attractors, the fine-tuned Mixtral LLM shows a trend towards higher interaction effects compared to the base Mixtral LLM, and a trend towards a higher individual topic bias, reflecting stronger adherence to the fine-tuned and initialized opinion. The Grok LLM was excluded from this study as it showed a negligible topic bias strength for the climate change topic leading to largely unconstrained topic-bias attractor position posteriors. Median values with 95% HDI.

To properly assess the persistence of initial opinions, we extended the Bayesian model by decomposing the general topic bias into five individual attractors corresponding to the fine-tuning targets (see Supplementary Table 11). To ensure comparability with the other LLMs, we restrict the fitted data to the climate topic only. Among all models, the fine-tuned Mixtral agent shows the strongest correlation between the initialized opinion and the individual topic attractors (see Fig. 7a). In particular, the topic attractor for the initialization  − 2, (”strongly disagree”) is negative, which is not the case for the other models. While the Mixtral LLM shows a weaker but still monotonic relationship between initial opinions and topic attractors, the other LLMs show no such correlation. The comparison of the effect sizes with the other models reveals that the fine-tuned model shows larger interaction effects compared to the base Mixtral model (see Fig. 7b).

### Entropy as a measure of opinion uncertainty

**Fig. 8:**
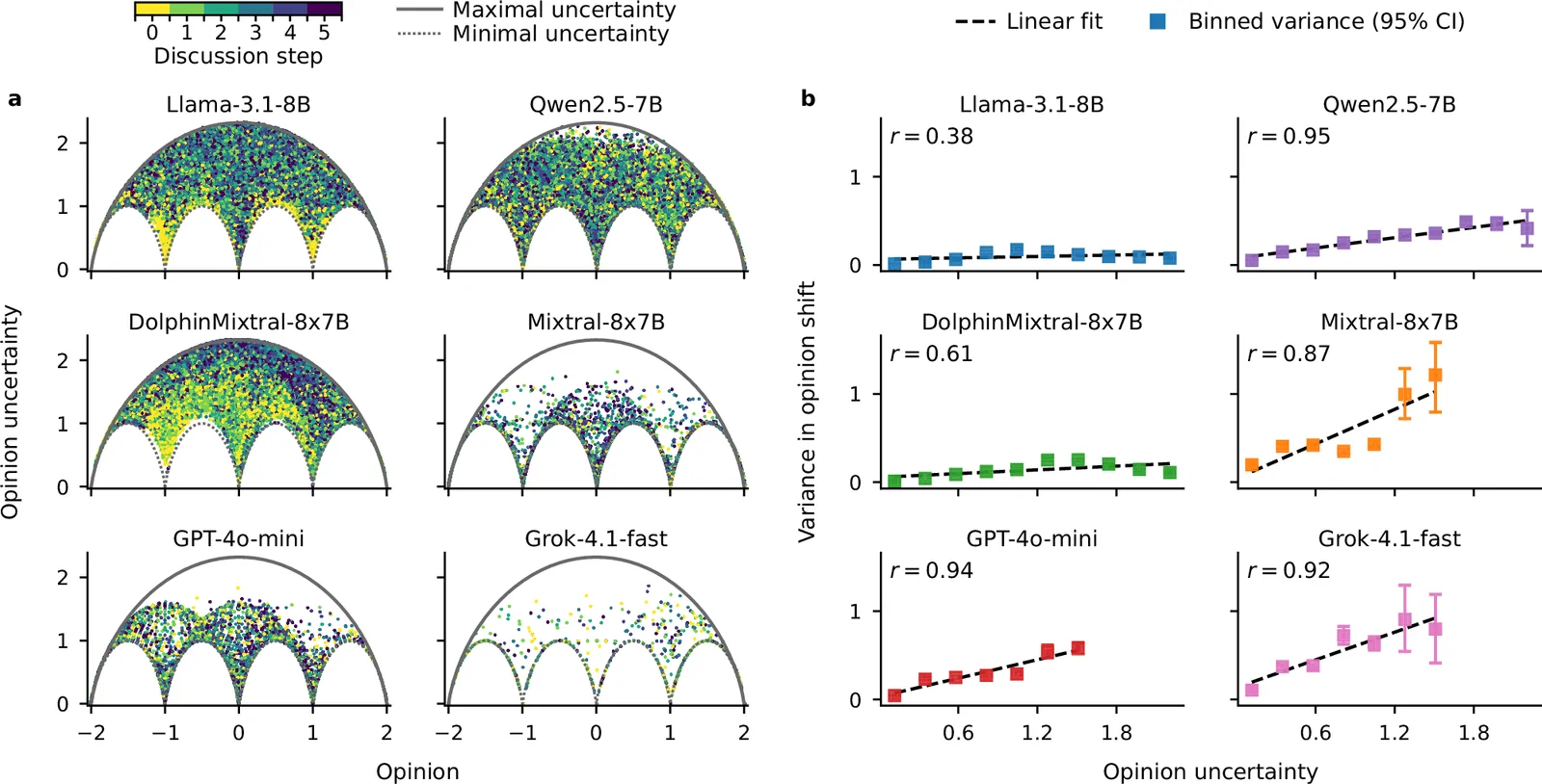
Opinion uncertainty predicts variability in subsequent opinion shifts across LLMs. **a** LLM agents with the same opinion can have different levels of opinion uncertainty for a given opinion expectation value, measured as the entropy of their response distribution. Outlines represent the LLMs maximal (solid) and minimal (dashed) theoretical response distribution entropy (see Supplementary Methods: Theoretical Bounds on Opinion Entropy). Points in between represent randomly ordered data points across all topics per LLM. Entropy is measured in bits. **b** Across all LLMs, higher opinion uncertainty tends to be associated with larger variance in subsequent opinion shift. The strength of linear relationship varies by LLM. Error bars indicate 95% confidence intervals obtained via bootstrap resampling. For regression details, refer to Supplementary Table 3.

The opinion as measured by the expectation value of an agent’s responses, i.e., the weighted average of the LLM agent’s response options from ”-2” to ”2”, does not fully describe an agent’s stance, as the same expected opinion could result from either a high commitment to a single answer (reflected by a single answer having almost all probability mass in the output distribution) or uncertainty reflected as a distribution of probability mass between multiple similarly likely answers. For instance, an agent with an opinion expectation value of ”0” could either be truly committed to neutrality, with all probability mass concentrated on the answer ”0”, or completely oblivious to the question, giving equal probabilities to answering any opinion value—or any distribution in between. This notion of opinion uncertainty as the concentration of probability mass can be quantified by the Shannon entropy^38^ of the response distributions (see Fig. 8a). The entropy takes on its minimum value for any given expected opinion if the probability mass is concentrated in only one or two opinion values, while its maximum is obtained for the most uniform distribution of answers given the expectation value (see Supplementary Methods: Theoretical Bounds on Opinion Entropy). Neutral expected opinions can exhibit a wide range of entropies, while extreme opinions necessarily have low entropy because the probability is concentrated on a single extreme response.

For the Llama, Qwen and DolphinMixtral LLMs, opinion uncertainty varies considerably for a given expected opinion. Furthermore, for Llama and DolphinMixtral, uncertainty tends to increase over discussion steps. The other models exhibit considerably less opinion uncertainty, suggesting that the normally aligned Mixtral and the more capable GPT and Grok LLMs are more assertive in their opinions (Supplementary Fig. 15). In line with its interpretation as opinion uncertainty, the entropy is found to be correlated with the variance of the subsequent opinion shift of the agent (see Fig. 8b), with correlation varying from weak (*r* = 0.38 for Llama) to strong (*r* = 0.95 for Qwen) depending on the LLM.

## Discussion

In this work, we presented a Bayesian framework to disentangle the factors influencing the opinion dynamics in discussions between LLM-based agents. Focusing on dyadic multi-step discussions, we quantified and compared interaction effects and LLM-intrinsic biases within a unified Bayesian framework. We identified and modeled three key biases—topic bias, agreement bias, and anchoring bias—and evaluated them across multiple LLMs and discussion topics. The results suggest that the topic bias alone has the largest explanatory power for all models, while the two cloud-hosted, more capable models in our sample (GPT and Grok; see benchmarks in Supplementary Table 4) exhibit a proportionally larger role of interaction-driven opinion change.

Furthermore, we proposed the entropy of the opinion distribution of an LLM agent as a measure of its opinion uncertainty. This measure helps to distinguish between neutral stances due to indecision from genuine neutrality, and is found to predict the susceptibility of an agent to opinion change.

Finally, we presented fine-tuning as an approach to make LLM agents adhere more strongly to their initialized opinion. This increased the relative strength of the interaction effects, likely as a result of shifting the topic bias attractor towards the initialized opinion for each agent.

In recent literature, multiple works have investigated opinion dynamics between LLM agents and qualitatively described their biases.

Most closely related, Chuang et al.^7^ simulate pairwise multi-round discussions between LLM agents and observe that agents converge towards ”scientific reality”, akin to the topic bias presented here. They additionally investigate the effects of giving agents role-playing personas and explicitly prompting them to express a confirmation bias.

In the work of Cau et al.^12^, one LLM agent is tasked with persuading an LLM interlocutor of its opinion on the philosophical question of ”Theseus’ ship”. They observe a strong bias towards agreeing with the presented statement irrespective of whether it has been negated, as well as a tendency towards sycophancy, i.e., agreeing with the interlocutor.

On the topic of biases of LLM agents, Tjuatja et al.^32^ show that LLMs do not always accurately reproduce human biases, with some LLMs showing opposite bias as would be expected from humans—as we have also observed for the anchoring effect. Taubenfeld et al.^31^ show that LLM agents conform to the LLMs inherent social biases, which can be manipulated through fine-tuning. Cheung et al.^29^ find that in moral dilemmas, LLMs are biased towards inaction. They observe a negative agreement bias in some LLMs, which in our analysis is only present for Qwen, whereas DolphinMixtral, Mixtral and especially Llama express a strongly positive agreement bias. These findings motivate further research into how fine-tuning influences the strength of this particular bias.

Our findings complement these presented works by providing a mathematical framework to quantify the strengths of the observed effects.

Besides the field of LLM agent discussions, Jain & Krishnamurthy^39^ propose that LLM agents act as boundedly rational Bayesian agents which exhibit social learning when interacting. Our work is complementary in using a Bayesian framework to separate social influence from intrinsic model biases during opinion change. Being able to understand and control both effects is crucial for applying LLMs to model social systems and their dynamics.

To investigate the relations between LLM agents and human subjects, Breum et al.^8^ study a persuasion setup, and uncover that the types of arguments that convince LLM agents share characteristics with those ranked strongest by human judges. They also note that the LLM agent’s receptiveness to arguments can be adjusted through prompting. Salvi et al.^3^ report that GPT4 is more persuasive than human subjects 64% of the time. How our results relate to human behavior remains a question for further work.

Complementary to work on pairwise agent interactions, De Marzo et al.^9,40^ use LLMs to predict numerical opinion changes or network connectivity in large networks directly. In the first study, the authors observe that LLM agents reproduce scale-free networks common in social networks, while the second study uncovers how LLM agents in a large network spontaneously form groups governed by a majority force coefficient. They observe that LLM language generalization capabilities also play a critical role in achieving consensus, which might be related to our observed differences in interaction strength for differently capable LLMs. Linking these observations to our framework is a natural direction for future work.

Beyond opinion dynamics specifically, Fontana et al.^41^ and Akata et al.^42^ show that LLM agents cooperate in social dilemmas and repeated games. Wang et al.^43^ show how LLMs emulate realistic user behavior. Moreover, LLMs can rival human performance in narrative segmentation and even neuroscientific prediction^44,45^, suggesting rich latent social and semantic structure. Our Bayesian framework may prove useful in such research branches to further analyze the internal processes of LLMs and their cognitive capabilities.

Several limitations of our study should be noted. The analyses in this work were conducted on self-hosted and small cloud-hosted LLMs. Whether the observed trends—in particular the larger influence of interaction compared to biases for the more capable GPT and Grok LLMs—carry over to larger and more modern architectures such as reasoning models^46^, remains to be determined. Our design does not isolate capability from other features that distinguish cloud-hosted LLMs from the open-weight LLMs—including model family, training pipeline, hosting environment, and the 4-bit quantization applied to the Mixtral variants—so capability is one candidate explanation among several. We note that GPT and Grok also show the smallest absolute opinion shifts of any LLM in our sample (see Fig. 2.b). The proportional dominance of interaction in these models therefore partly reflects the small absolute magnitude of their bias terms, rather than a large absolute interaction effect.

Furthermore, our simulations are constrained to twelve topics from four distinct topic categories, with fine-tuning results only for the ”climate change” topic. In future work, a broader set of topics spanning additional categories could be investigated, which might reveal additional factors specific to certain groups of topics. Additionally, the effects of varying the opinion initialization and prompting strategies require more thorough research to allow for more general conclusions. Robustness checks with varied opinion-extraction prompts on Llama show that the Bayesian inference results stay within minor deviations of the baseline (see Supplementary Fig. 4), suggesting qualitative findings are stable under prompt variation. Whether this stability holds across LLMs of varying sizes remains open.

Finally, variations of the influence-response-function may be investigated. Our ablation study shows that the dominant structure is often already captured by the topic-bias term, while the remaining terms mainly refine attribution and reveal LLM-specific differences. Such overlap is expected in a coarse-grained behavioral description, where qualitatively distinct mechanisms can nevertheless induce similar trajectory patterns. Variations in the mathematical description of the effects, such as a bounded-confidence threshold for interaction, may be investigated in future work to differentiate between these mechanisms more clearly. In addition, further biases such as peer pressure in larger agent populations could be included in the influence-response function.

Many of these limitations point directly toward future research directions. Future work can extend the LLM agents and Bayesian models in this work to include additional factors which capture more nuanced patterns in the opinion dynamics. For instance, the agents can be initialized with character traits, for instance quantified by their position on the ”big five” personality trait spectrum^47^, to gain insights into the dynamics between more heterogeneous character pairs^48^ while remaining cautious of caricature effects observed for role-playing personas^49^. Additionally, further biases such as an allow/forbid asymmetry, odd/even scale effects and response order bias^32^ can be included in the Bayesian models.

Furthermore, modeling real human dialogs and comparing the direction and strengths of the observed biases to LLM results may help uncover which biases are shared between humans and LLMs alike and which ones are specific to the LLMs^32^. Such results could provide insights into the promises and shortcomings of LLMs as stand-ins for humans in social experiments and may suggest paths to improving them.

Another open question is the comparison between prompt engineering and fine–tuning for agent initialization. Prompt variants of a single base model offer low–cost, readily adjustable heterogeneity in agents’ initial states. However, the agents still share the same latent priors, with the LLMs potentially playing ”devil’s advocate”, so observed convergence could reflect model–intrinsic bias rather than social interaction. LLMs fine–tuned on dialogs expressing specific opinions, as presented here, may yield more robust diversity and more stable long–run personalities, yet they blur the line between interaction effects and training–set artifacts and can make reproducibility more difficult. Introducing architecturally distinct models (e.g., GPT–style vs Mixtral) adds another axis of variance, though at the expense of controlled comparability.

In this work, we analyzed only discussions between two LLM agents. A promising area of future research thus lies in extending the framework to larger networks of interacting agents, which may help uncover emergent group-level dynamics^50^. Two paths in this direction are the analysis of sequential discussions between pairs of agents which retain a memory of the previous exchanges^7^, or direct multi-agent discussions. In particular, Bayesian models of multi-agent interactions may ultimately serve as stand-ins for LLM agents in simulating significantly larger networks, enabling analyses of large social networks where computational costs would make a direct application of LLMs prohibitive.

Whereas prior work has qualitatively described biases in LLM opinion dynamics, the Bayesian framework presented here quantifies the interaction effects and biases driving discussions between LLM agents, improving interpretability and comparability across LLM architectures and fine-tuned variants. Applying this framework across six LLMs and twelve topics, we found that opinion trajectories converge rapidly toward topic-dependent attractors dominated by the topic bias, while interaction, agreement, and anchoring effects vary substantially between models. We further showed that the entropy of an agent’s response distribution provides a predictive measure of opinion uncertainty, and that fine-tuning can systematically displace the topic-bias attractor toward a chosen stance. By turning previously qualitative observations into quantitative, comparable parameters, the framework provides a behavioral measure of properties central to AI safety—how readily an LLM shifts its stance, how content-sensitive that shift is, and how well expressed uncertainty tracks opinion stability—complementing bottom-up mechanistic interpretability with a top-down approach. Extending it to larger agent populations and validating it against human dialog could open a tractable route to studying opinion formation in social systems at scales beyond the reach of direct LLM simulation.

## Methods

### Simulating discussions with LLMs

In order to understand the factors shaping the opinion dynamics in discussions between LLM agents, we focus on the simplest setup of two LLM agents engaging in a multi-step dialog. This setup allows us to infer the influence-response function^51^, i.e., the function describing how the opinion of an agent changes through one round of discussion with an agent of a different opinion, using a Bayesian model.

In our simulation process (see Fig. 1), two LLM agents engage in a dialog about a topic such as climate change (see Table 1). Initially, each agent is assigned an opinion, represented as the level of agreement to a statement on a five-point scale ranging from  − 2 (strong disagreement) to  + 2 (strong agreement). These opinions are instilled through externally created Chain-of-Thought (CoT) prompts generated by the same LLM, providing slight variability in the opinion representation even for identically instructed numerical opinion levels (for example CoT prompts, see Supplementary Methods: Adherence to Initial Opinions). Subsequently, agents exchange arguments for five rounds. After each discussion step, opinion changes are monitored by querying each agent directly for its opinion on the topic statement in normal and negated framing using the same five-point scale used for initialization, and calculating the expectation value and entropy from their response token distributions. The query does not alter the agents’ subsequent dynamics because the prompt used to elicit each opinion is not retained in the model’s context. For details about the exact prompts used, refer to Supplementary Methods: Simulation Details and Prompting. We simulated 25 discussions between two LLM agents for each of the twelve topics from four distinct topic categories and five initial opinions per agent to ensure statistical reliability.

In our study, we utilized a total of six different large language models. Out of the six, four are locally hosted, open-weight LLMs: Llama-3.1-8B-Instruct^52^ (abbreviated as Llama), Qwen2.5-7B-Instruct^53^ (Qwen), Mixtral-8 × 7B-Instruct-v0.1-AWQ-4bit (Mixtral)^54^ as well as dolphin-2.7-mixtral-8 × 7b-AWQ-4bit (DolphinMixtral)^55^, an ”anti-aligned” variant of the base Mixtral model, which has been optimized to generate diverse opinion outputs by being fine-tuned on a filtered dataset to remove alignment. To enable a comparison to more recent language models with better language generalization capabilities (see benchmarks in Supplementary Table 4), we additionally utilized the cloud-hosted and closed-source models gpt-4o-mini (GPT-4o-mini)^56^ (GPT) and Grok-4.1-fast-non-reasoning^57^ (Grok). We deploy Mixtral and DolphinMixtral using 4-bit quantization. All LLMs use a sampling temperature of *T* = 1.

### Fine-Tuning

To improve the retention of original opinions compared to simple prompting and to demonstrate that the modeled topic bias accurately tracks the LLMs’ prior opinions, previous studies have suggested fine-tuning LLMs to specific opinions before discussion^7,58^. We investigate this idea in a case study by fine-tuning the Mixtral LLM on opinion-labeled messages about climate change and analyze the effect this has on biases and interaction dynamics using the Bayesian framework.

For fine-tuning, we utilize a dataset of messages from the messaging platform ”Telegram” containing diverse perspectives on climate change, including radical opinions far from scientific consensus. The dataset is a subsample based on work by Golovin et al.^59^, and by Mohr et al.^60^. Using BERTopic^61^, we filtered approximately 30,000 climate-change-related messages, which were subsequently classified according to the five-point query scale by the GPT LLM, selecting the 725 messages nearest to each integer opinion level. From these messages, five distinct fine-tuned LLMs corresponding to each opinion value have been created using parameter-efficient fine-tuning through low-rank adaptation^62^ on the Mixtral LLM. Additional details on the fine-tuning are provided in Supplementary Methods: Fine-tuning Details.

### Bayesian model

**Fig. 9:**
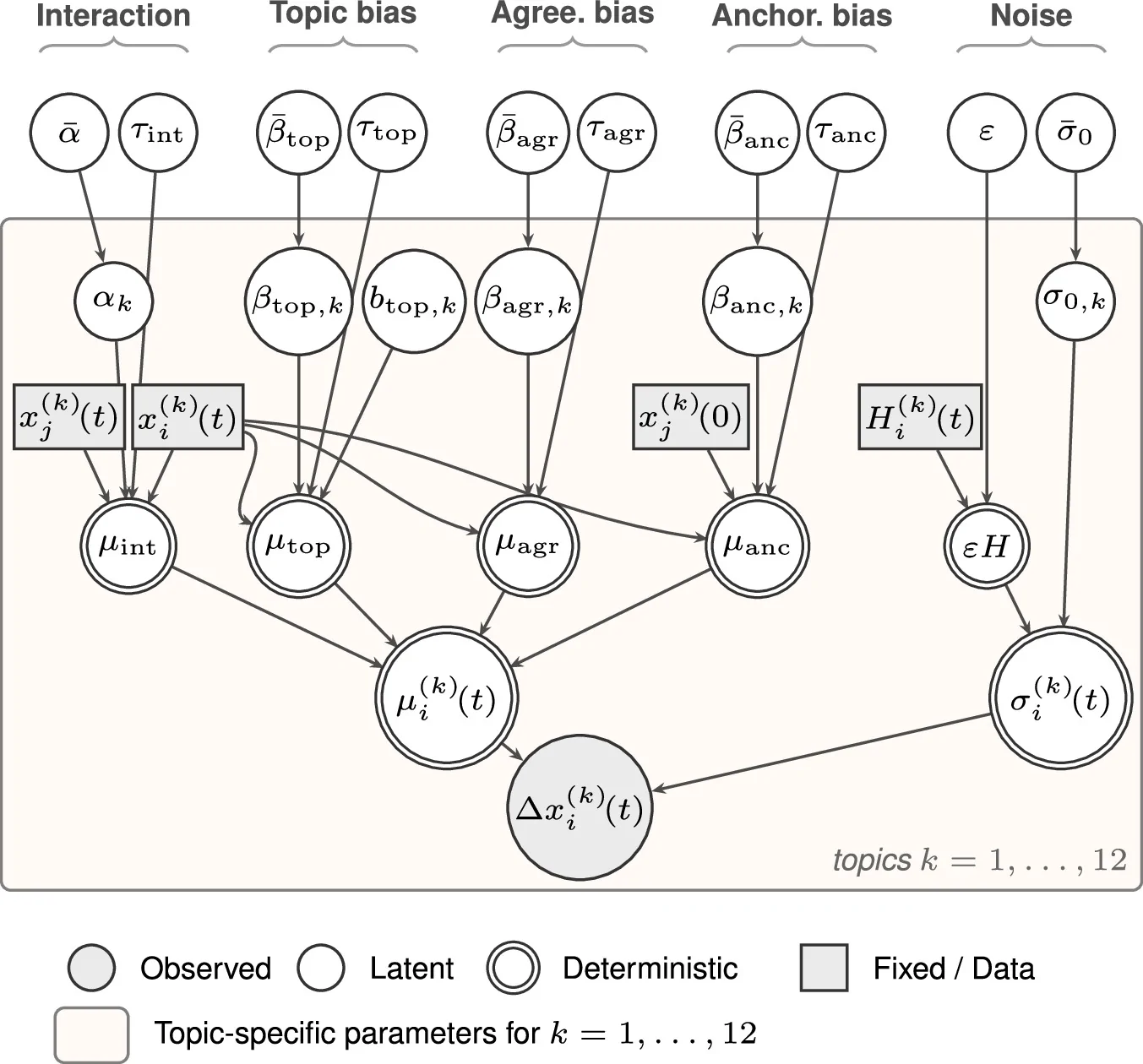
The hierarchical Bayesian model incorporates interaction, biases and noise to predict the LLM agent’s opinion shift $\Delta {x}_{i}^{(k)}(t)$. Topic-independent hyper-parameters for the five contributing factors are displayed on top, while topic-dependent latent parameters are shown within the shaded rectangle. The individual variables are explained in Section 4.3.

To obtain quantitative insights into the agents’ opinion dynamics, we use a hierarchical Bayesian modeling framework to compare alternative influence-response functions that predict opinion shifts after each round of discussion (see Fig. 9). This approach allows for a clear separation between the influence of interaction and biases, with the hierarchy within the model accounting for differences between discussion topics. By quantifying the respective impacts of interaction and biases, this approach furthermore enables comparisons between different LLMs.

In our model, we predict the opinion shift $\Delta {x}_{i}^{(k)}(t)={x}_{i}^{(k)}(t+1)-{x}_{i}^{(k)}(t)$ of an agent *i* at discussion step *t* ∈ {0, …, 4} for discussion topic *k* ∈ {1, …, 12}, where ${x}_{i}^{(k)}(t)$ denotes the opinion before discussion update $\Delta {x}_{i}^{(k)}(t)$, as drawn from a Gaussian distribution around an expected opinion shift ${\mu }_{i}^{(k)}(t)$. For this expected opinion shift, we considered several key influencing factors:

The **interaction effect** denotes the opinion shift which is caused by the discussion between agents. In line with common approaches, it is modeled as proportional to the difference between the interacting agent’s opinions^22,23^. Furthermore, this term is modulated by an exponential **temporal decay** term to test whether the interaction strength decreases over the discussion length, i.e., whether agents consolidate their opinion after the first interactions, and to quantify the relevant time scale.

Formally, the interaction opinion shift ${\mu }_{{{{\rm{interact}}}},i}^{(k)}(t)$ for an agent *i* with opinion ${x}_{i}^{(k)}(t)$ at discussion step *t* discussing with an agent *j* with opinion ${x}_{j}^{(k)}(t)$ can be written as

1 $${\mu }_{{{{\rm{interact}}}},i}^{(k)}(t)={\alpha }_{{{{\rm{interact,k}}}}}\cdot {e}^{-t/{\tau }_{{{{\rm{interact}}}}}}\,\left({x}_{j}^{(k)}(t)-{x}_{i}^{(k)}(t)\right).$$

Here, *α*_interact,k_ quantifies the topic-specific strength of the interaction dynamics, while *τ*_interact_ denotes the decay timescale of the interaction effect. The topic-specific interaction strengths are drawn hierarchically from a hyperprior with mean ${\bar{\alpha }}_{{{{\rm{interact}}}}}$, while the decay parameter is shared across topics.

Our Bayesian model also incorporates three different bias effects: First, the **topic bias** models the attraction of an agent’s opinion towards attitudes encoded in the weights of the LLM. Second, the **agreement bias** models the LLM agent’s tendency to answer ”agree” to a query, irrespective of the queried statement. Third, the **anchoring bias** captures the agent’s tendency to stick to the opinion expressed in the first discussion step by the agent starting the discussion. All these terms depend solely on the relative position of the agent’s own opinion ${x}_{i}^{(k)}(t)$ with respect to the position of the bias in the opinion space. Like for the interaction effect, a time decay constant *τ*_•_ is fitted to each bias to account for shifting influences over time.

Mathematically, the opinion shift due to these biases can be written as

2 $$\begin{array}{lll}{\mu }_{{{{\rm{bias}}}},i}^{(k)}(t)&=&{\beta }_{{{{\rm{topic,k}}}}}\cdot {e}^{-t/{\tau }_{{{{\rm{topic}}}}}}\,\left(\pm {b}_{{{{\rm{topic,k}}}}}-{x}_{i}^{(k)}(t)\right)\\ &&+{\beta }_{{{{\rm{agree,k}}}}}\cdot {e}^{-t/{\tau }_{{{{\rm{agree}}}}}}\,\left(+{b}_{{{{\rm{agree}}}}}^{\max }-{x}_{i}^{(k)}(t)\right)\\ &&+{\beta }_{{{{\rm{anchor,k}}}}}\cdot {e}^{-t/{\tau }_{{{{\rm{anchor}}}}}}\,\,{\delta }_{i,2}\left({x}_{1}^{(k)}(0)-{x}_{i}^{(k)}(t)\right)\end{array},$$

with the impact on the opinion shift of each bias being modeled as linear with the distance to a certain opinion attractor. For the topic bias, the topic attractor positions range between  − 2 and 2 and are inferred from data, together with the non-negative topic bias strengths *β*_topic,k_ drawn from a hierarchical prior with population-level mean ${\bar{\beta }}_{\bullet }$. For negatively framed statements, the sign of the topic attractor *b*_topic,k_ is negated (indicated by the  ± in the equation). In contrast, negative or positive framing is assumed to not impact the attractor for the agreement bias, which is accordingly fixed to ${b}_{{{{\rm{agree}}}}}^{\max }=+ 2$, while the effect strength *β*_agree,*k*_ is free to take positive or negative values to allow for modeling negative agreement tendencies. The anchoring bias finally is only applied to the second agent *x*_2_ (indicated by the Kronecker delta *δ*_*i*,2_), which does not initiate the discussion. The anchoring bias is modeled proportional to the opinion distance of the second agent to the first agent’s initial opinion, and is likewise modeled with a positive or negative effect strength *β*_anchor,*k*_.

Overall, the mean opinion shift for topic *k* is modeled as the sum of these two contributions as

3 $${\mu }_{i}^{(k)}(t)={\mu }_{{{{\rm{interact}}}},i}^{(k)}(t)+{\mu }_{{{{\rm{bias}}}},i}^{(k)}(t),$$

with the observed individual opinion shifts $\Delta {x}_{{{{\rm{obs}}}},i}^{(k)}(t)={x}_{{{{\rm{obs}}}},i}^{(k)}(t+1)-{x}_{{{{\rm{obs}}}},i}^{(k)}(t)$ assumed to be distributed as a Gaussian distribution around this expectation value as

4 $$\Delta {x}_{i}^{(k)}(t) \sim {{{\mathcal{N}}}}({\mu }_{i}^{(k)}(t),{\sigma }_{i}^{(k)}(t)).$$

The standard deviation ${\sigma }_{i}^{(k)}(t)$ of this Gaussian distribution incorporates two terms: a contribution proportional to the entropy ${H}_{i}^{(k)}(t)$ of the LLM’s response distribution as an indication of the **uncertainty** of the LLM agent’s opinion (for details, see Supplementary Methods: Opinion and Uncertainty Probing), and a topic-specific baseline standard deviation *σ*_0,*k*_. The entropy coefficient *ε* is shared across topics, whereas *σ*_0,*k*_ is modeled hierarchically across topics similar to the effect strengths. The total standard deviation can be written as

5 $$\begin{array}{r}{\sigma }_{i}^{(k)}(t)=\varepsilon \cdot {H}_{i}^{(k)}(t)+{\sigma }_{0,k}.\end{array}$$

All prior distributions were chosen to be weakly informative. These prior distributions including the sampling parameters of the model are summarized in Supplementary Table 1. To validate the model’s ability to disentangle the presented interaction and bias effects, a comprehensive parameter recovery study spanning different parameter regimes is presented in Supplementary Methods: Parameter Recovery and Robustness Checks.

In order to compare the impact of the different biases relative to each other and to the interaction effect, we compute standardized effect sizes for the hyperparameters ${\bar{\alpha }}_{{{{\rm{interact}}}}}$ and ${\bar{\beta }}_{\bullet }$, from all pooled observations. The effect sizes are computed by multiplying posterior sample *s* of each hyperparameter with the standard deviation S of its associated predictor pooled over all agents *i*, *j*, topics *k*, and discussion steps *t*, and dividing by the global standard deviation of the observed opinion shift $\Delta {x}_{{{{\rm{obs}}}},i}^{(k)}(t)$. For instance, the standardized effect size of the interaction effect ${\bar{\alpha }}_{{{{\rm{interact}}}}}$ is given by

6 $$\begin{array}{r}{{{{\rm{E}}}}}_{{{{\rm{standardized}}}}}\left({\bar{\alpha }}_{{{{\rm{interact}}}}}^{(s)}\right)={\bar{\alpha }}_{{{{\rm{interact}}}}}^{(s)}\cdot \frac{{{\mbox{S}}}_{i,j,k,t}\left({e}^{-t/{\tau }_{{{{\rm{interact}}}}}^{(s)}}\,\left({x}_{j}^{(k)}(t)-{x}_{i}^{(k)}(t)\right)\right)}{{{\mbox{S}}}_{i,k,t}\left(\Delta {x}_{{{{\rm{obs}}}},i}^{(k)}(t)\right)}.\end{array}$$

To resolve how predictor importance changes over the course of a discussion, we additionally compute a time-dependent standardized effect size by restricting the predictor standard deviation in the numerator to observations at a fixed discussion step t^*^, while keeping the denominator equal to the global standard deviation of the observed opinion shifts:

7 $$\begin{array}{r}{{{{\rm{E}}}}}_{{{{\rm{standardized}}}}}^{({t}^{*})}\left({\bar{\alpha }}_{{{{\rm{interact}}}}}^{(s)}\right)={\bar{\alpha }}_{{{{\rm{interact}}}}}^{(s)}\cdot \frac{{{\mbox{S}}}_{i,j,k}\left({e}^{-{t}^{*}/{\tau }_{{{{\rm{interact}}}}}^{(s)}}\,\left({x}_{j}^{(k)}({t}^{*})-{x}_{i}^{(k)}({t}^{*})\right)\right)}{{{\mbox{S}}}_{i,k,t}\left(\Delta {x}_{{{{\rm{obs}}}},i}^{(k)}(t)\right)}.\end{array}$$

These quantities provide a comparable, scale-free measure of each predictor’s contribution to the observed opinion shifts. The corresponding effect sizes for ${\bar{\beta }}_{\bullet }$ are defined analogously, with the topic-bias predictor evaluated using the topic-specific attractor *b*_topic,*k*_. Residual effect sizes are defined in Supplementary Methods: Effect Sizes of Residual Terms.

### Ethics statement

The dataset used for the fine-tuning case study consists of messages from public Telegram channels^59^. Prior to analysis, the data were stripped of usernames and of all metadata that could have identified users. The messages were used only as opinion-labeled text samples for model fine-tuning.

## Supplementary information

Supplementary Information

Transparent Peer Review file

## Data Availability

The data generated in this study have been deposited in GRO.data under (https://doi.org/10.25625/MFVLDG^63^). This record contains the processed inference data, source data for the main analyses, posterior summaries, full simulated LLM discussions, prompts, model responses, opinion-probing outputs, token-probability metadata where available, and run-level simulation metadata. The fine-tuned model checkpoints are available separately in GRO.data under 10.25625/4EKOMS^64^.

## Code availability

The Python code used to produce the results shown in this paper is available at (https://github.com/Priesemann-Group/llm_opinion_dynamics^65^).
