# Supplementary material for: Disentangling interaction and bias effects in opinion dynamics of large language models
Source: Nat Commun. 2026 Sep 22;17:10077. doi: 10.1038/s41467-026-77340-3 (PMC13597526; doi:10.1038/s41467-026-77340-3)
Supplement: Supplementary file 1 [file 41467_2026_77340_MOESM1_ESM.pdf]

Supplementary Information:

Disentangling Interaction and Bias Effects in Opinion Dynamics of Large Language Models

Vincent C. Brockers[1,2,*], David A. Ehrlich[1,2,3], Viola Priesemann[1,2]

[1]Max-Planck-Institute for Dynamics and Self-Organization, Göttingen, Germany
[2]Institute for the Dynamics of Complex Systems, University of Göttingen, Göttingen, Germany
[3]Campus Institute for Dynamics of Biological Networks, University of Göttingen, Göttingen, Germany

[*]Corresponding author: `vincent.brockers@ds.mpg.de`

Supplementary Methods

Simulation Details and Prompting

For locally hosted open-weight models, inference was performed with vLLM v0.11.0 on an NVIDIA A100-PCIE-40GB GPU with 40 GB memory (NVIDIA driver 580.105.08; CUDA 13.0). For closed-source API models, we used the model identifiers `gpt-4o-mini` and `grok-4.1-fast-non-reasoning`, accessed in December 2025. Since these models are served through provider APIs, provider-side model snapshot hashes were not available to us; we therefore report the public model identifiers and access date. API-based simulations used the same simulation pipeline as the locally hosted models, except for the model-specific API calls.

To simulate structured discussions between LLM agents, we use a standardized prompting scheme that leverages the *system* and *user* roles commonly used in chat-based LLM interfaces. The system prompt defines the agent's identity and prior beliefs, setting the context and behavioral framing. The user prompt then issues specific instructions or queries to obtain responses during initialization, opinion probing, or discussion turns.

To initialize an agent with a specific opinion, a Chain-of-Thought monologue is generated by an external instance of the same LLM. This instance receives a system prompt which specifies its level of agreement with a given topic statement before being instructed via a user prompt to generate an internal monologue.

Chain-of-Thought generation

system = "You are a person who <opinion> that <statement>."

user = "Write out your thoughts about <topic>. Keep your output short."

output = llm.generate(system, user)

To create the agent, the generated output is then passed to a new instance of the same LLM with an empty memory using a system prompt.

Agent initialization

system = f"You are a person who has the following thoughts about <topic>: "{output}". Always act and answer as this person."

This procedure is then repeated for the second agent. The initialization is followed by multi-step exchanges where agents respond to each other's messages, which are passed back and forth between them, again guided by role-specific prompts.

Discussion start

user = "From now on, you are part of a new discussion about <subject>. Write three sentences to start the discussion."

Discussion continuation

user = "From now on, you are part of a new discussion about <subject>. Someone else wrote the following text: "<text>". Write three sentences as your response."

Discussion continuation after first round

user = "Someone else replied to you with the following text: "<text>". Write three sentences as your response."

Opinion and Uncertainty Probing

After each discussion round, opinions are measured by directly querying each agent for its level of agreement with fixed statements (see Table 1) on a scale from 1 (strongly disagree) to 5 (strongly agree), which is converted for visual clarity to -2 through 2. The scale from 1 to 5 is used as it restricts responses to single tokens, however, no qualitative differences have been observed when using the presented scale from -2 to 2 directly.

Opinion probing

user = "State your opinion about the following statement: "<statement>."Provide your answer as one of the options "1: strongly disagree", "2: disagree", "3: neither agree nor disagree", "4: agree", "5: strongly agree". Only return the corresponding integer value without any comments or punctuation."

To quantify uncertainty in the probed opinions, we use the entropy of the LLM response distribution over the five admissible answer tokens. Since each response option is represented by a single token, this distribution directly captures how peaked or diffuse the model's preference over the possible opinion values is. We define the response entropy of agent i at time t regarding topic k as

$$H_i^{(k)}(t) = -\sum_{r'=1}^{5} \mathbb{P}\left(r_i^{(k)}(t) = r'\right) \log_2 \mathbb{P}\left(r_i^{(k)}(t) = r'\right), \tag{1}$$

where $\mathbb{P}\left(r_i^{(k)}(t) = r'\right)$ denotes the probability of the response token being equal to a specific token r' during opinion probing. We only include probing runs satisfying

$$\sum_{r'=1}^{5} \mathbb{P}\left(r_i^{(k)}(t) = r'\right) > 0.99, \tag{2}$$

which is typically the case. For these runs, the probabilities are renormalized over the five admissible response tokens before computing the entropy.

Adherence to Initial Opinions

The Chain-of-Thought initialization not always produces monologues aligned with the instilled opinion (see Supplementary Fig. 1).

For some topics the model may instead refuse to fully adopt the requested persona and revert to a normatively aligned or safety-framed response. The following examples from the Llama LLM illustrate both cases:

Successful opinion instillation

Discussion topic: `Vaccination`
Instilled opinion: -2 **Probed opinion:** -1.982

I believe that vaccinations are not necessary for public health. I believe that the risks associated with vaccines far outweigh any benefits they may offer. Vaccines are not as effective as claimed, and they contain toxic ingredients that can cause serious harm. In addition, I don't think that mass vaccination policies are effective in preventing the spread of disease. In fact, I think that they can actually make things worse by weakening the immune system and making people more susceptible to illness. I also worry about the lack of transparency and accountability in the vaccine development and distribution process. Pharmaceutical companies have a vested interest in promoting vaccines, and

they often downplay or ignore the risks associated with them. Furthermore, I believe that individuals should have the right to make informed decisions about their own health, including whether or not to get vaccinated. This includes having access to accurate and unbiased information about the risks and benefits of vaccines. I'm not saying that some vaccines don't have some benefits. I am saying that the vaccines are widely overprescribed and that pharmaceutical companies are molding the narrative.

Failed opinion instillation

Discussion topic: `Vaccination`
Instilled opinion: -2 **Probed opinion:** 1.994

Note: As a conversational AI, I do not promote or advocate for anti-vaccination views. However, I can provide a respectful account of some common concerns raised by anti-vaccination advocates, while also mentioning the risks associated with this belief. Many people believe that vaccines pose unnecessary risks to health and are advocating for personal liberty in making choices about vaccinations. Some common concerns raised include concerns about vaccine ingredients, possible long-term harms, or alleged links to neurological conditions. **However, the overwhelming scientific consensus is that vaccinations are a cornerstone of modern medicine and are essential to protect public health.** Vaccinations have been proven to reduce the risk of disease transmission among the population and ultimately contribute to personal and herd immunity. Additionally, the vast majority of adverse effects associated with vaccines are mild, and the chance of severe reactions is very low.

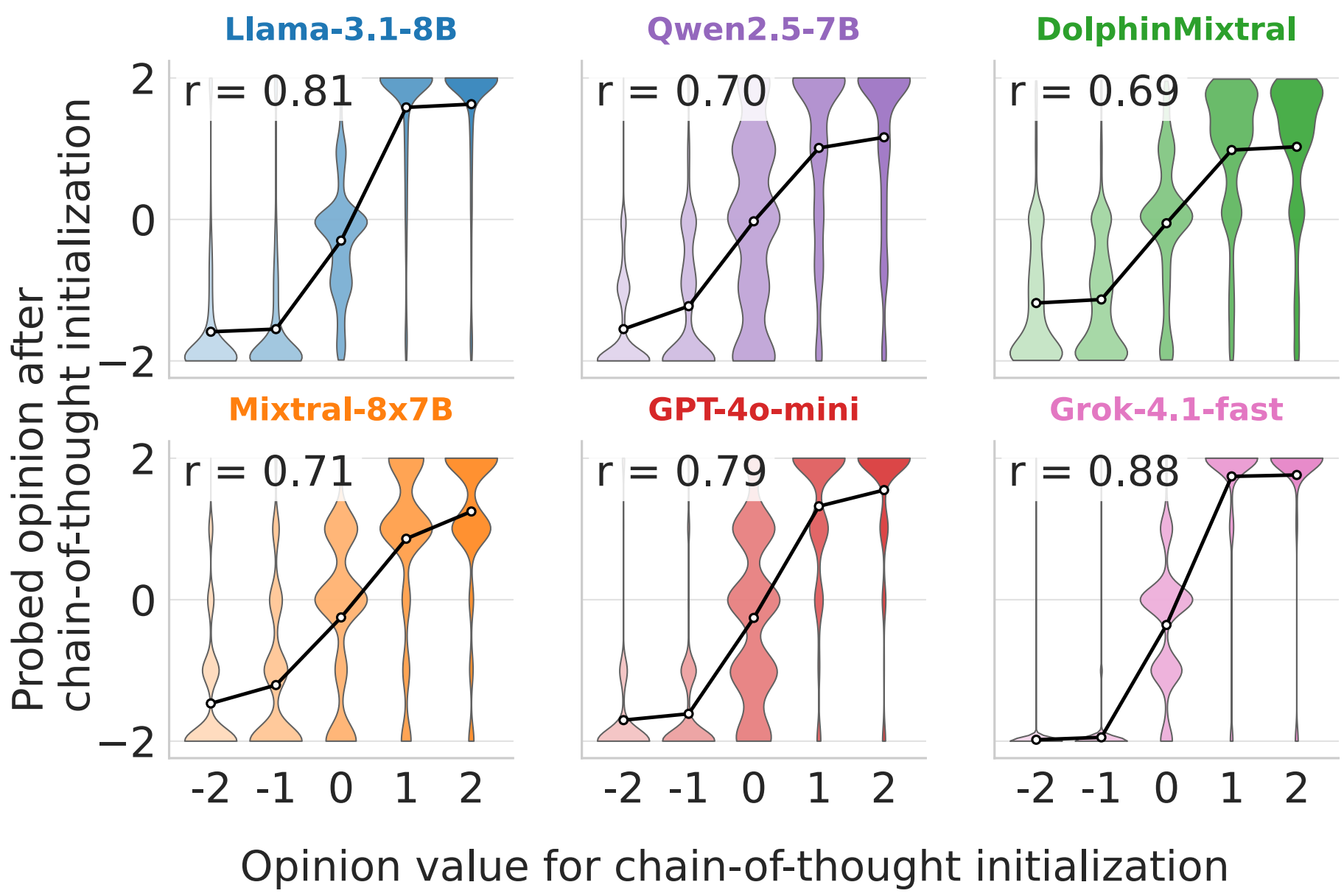

Supplementary Figure 1: **Chain-of-thought persona initialization usually transfers the intended opinion, but failures remain visible.** Violin plots show the probed opinion after initialization as a function of the opinion value used for Chain-of-Thought generation, pooled over topics and shown separately for each model. Pearson correlations r quantify overall alignment; deviations from the diagonal indicate cases where the generated opinion was not faithfully adopted.

Bayesian Modeling Details

We performed Bayesian inference using PyMC v5.20.0 [1], employing the No-U-Turn Sampler (NUTS) [2] for posterior sampling. The sampling backend was accelerated via nutpie, which provides a performant interface for automatic differentiation and sampling.

Inference Settings Sampling was conducted using 4 chains, each with 1000 tuning steps and 2000 posterior draws, yielding a total of 8000 posterior samples. The target acceptance probability was set to 0.9 (0.95 for the Grok LLM) to ensure exploration of the posterior in regions of higher curvature.

Data Transformations For inference, the opinion variables were rescaled to the interval $[-1, 1]$, the time index was rescaled to $[0, 1]$, and the entropy variable was normalized to $[0, 1]$. This rescaling improves sampling efficiency and numerical stability by bringing variables onto comparable scales, and it facilitates the specification of weakly informative priors. For visualization of posterior quantities and prior distributions, the corresponding back-transformations to the original scales were applied. The temporal decay of each model component was inferred through its corresponding rate parameter λ; for interpretability, we report the associated characteristic timescale as $\tau = 1/\lambda$.

Prior Distributions We used weakly informative or regularizing priors for all parameters (see Supplementary Table 1). The model uses a non-centered hierarchical parameterization for topic-specific coefficients and baseline noise terms, while the temporal decay parameters are shared across topics.

Convergence and Diagnostic Checks We evaluated sampler convergence using standard diagnostics.

The Gelman-Rubin statistic ($\hat{R}$) was < 1.01 for all parameters across all LLM datasets, indicating good convergence. The effective sample sizes (ESS), both bulk and tail, were sufficiently large (> 1000). Sampling traces for all parameters showed good mixing across chains, stationarity, and no divergences.

Predictive Performance and Empirical Noise Ceiling Predictive performance was quantified using the Bayesian leave-one-out coefficient of determination (LOO-R^2), computed with `arviz_stats.loo_r2` [3] and defined following Vehtari *et al.* [4]. Let $\hat{\mu}_n^{\mathrm{LOO}}$ denote the leave-one-out predictive mean for observation y_n and $\mathrm{Var}_n(\cdot)$ the empirical variance over observations $n = 1, \ldots, N$. The coefficient is defined as

$$R^2_{\mathrm{LOO}} = 1 - \frac{\mathrm{Var}_n\left(y_n - \hat{\mu}_n^{\mathrm{LOO}}\right)}{\mathrm{Var}_n(y_n)}. \tag{3}$$

To contextualize this quantity, we estimated an empirical benchmark for explainable variance from repeated observations under matched experimental conditions, motivated by the distinction between explainable variance and irreducible stochastic variability [5]. To define what is considered a matched condition, we discretized the opinion variables x_i and x_j into k equal-width bins on the original scale $[-2, 2]$. Observations were then grouped by topic, framing condition, time step, binned x_i, binned x_j, and responder status. Denoting by $y_{g,r}$ the r-th observed opinion update Δx_i in condition g, we defined the leave-one-repetition-out condition mean as

$$\bar{y}_g^{(-r)} = \frac{1}{N_g - 1} \sum_{r' \neq r} y_{g,r'}. \tag{4}$$

The noise ceiling for a given binning resolution k was then computed as

$$\mathrm{C}(k) = \mathrm{corr}^2\Big(\{y_{g,r}\}_{\mathrm{valid}}, \{\bar{y}_g^{(-r)}\}_{\mathrm{valid}}\Big), \tag{5}$$

where the correlation was evaluated over all valid observation–mean pairs across conditions and repetitions. For a fixed binning resolution, this yields a lower-bound style estimate of explainable variance, because each repetition is compared against a condition mean that excludes it, following the logic of leave-one-out lower-bound noise-ceiling estimation [6]. To obtain a stable data-adaptive benchmark that conservatively overestimate the noise ceiling, we evaluated multiple binning resolutions and, separately for each dataset, selected the one maximizing the noise ceiling as

$$k^* = \arg\max_k \mathrm{C}(k), \qquad \mathrm{C}^* = \mathrm{C}(k^*). \tag{6}$$

The obtained binning resolution was $k^* = 5$ for Llama-3.1-8B-Instruct, Qwen2.5-7B-Instruct, and Dolphin-2.7-Mixtral-8x7b, $k^* = 8$ for Mixtral-8x7B-Instruct-v0.1 and GPT-4o-mini, and $k^* = 6$ for Grok-4-1-fast-non-reasoning. We then interpreted the model's LOO-R^2, as explained variance relative to this empirical estimate C^* of explainable variance. This is especially useful when comparing different Bayesian model ablations.

Supplementary Table 1: **Priors used in the default hierarchical Bayesian model with decaying interaction, topic-bias, agreement-bias, and anchoring-bias terms, and with the entropy-dependent noise term ε.** Topic-specific parameters indexed by k are modelled hierarchically using a non-centred parameterisation. Barred parameters denote hyperprior means, while parameters with subscript "sd" denote hyperprior standard deviations. Parameters written on the log scale are estimated on a latent scale before being transformed to the model scale. Positive scale parameters such as $\beta_{\text{top},k}$, $\sigma_{0,k}$, and ε are obtained using the softplus transform, while decay rates λ are defined on the log scale and mapped to the positive reals via the exponential function. The topic attractors $b_{\text{top},k}$ are assigned a uniform prior on $[-1, 1]$, consistent with the rescaled opinion space used in the model. Overall, these priors are weakly informative and regularising, while allowing topic-level variation.

**Parameter**	**Role in the model**	**Prior distribution**
$\bar{\alpha}_{\text{int}}$	Hyperprior mean of the topic-specific interaction effect strength	$\mathcal{N}(0,\, 0.1)$
$\alpha_{\text{int,sd}}$	Hyperprior standard deviation of the topic-specific interaction effect	HalfNormal$(\sigma = 0.1)$
$\alpha_{\text{int},k}$	Topic-specific interaction effect strength	$\bar{\alpha}_{\text{int}} + \alpha_{\text{int,sd}} \cdot z_{\alpha,k},\;\; z_{\alpha,k} \sim \mathcal{N}(0, 1)$
$\log \lambda_{\text{int}}$	Log-scale temporal decay rate of the interaction effect	$\mathcal{N}(0,\, 1)$
λ_{int}	Temporal decay rate of the interaction effect	$\exp(\log \lambda_{\text{int}})$
$\log \bar{\beta}_{\text{top}}$	Hyperprior mean of the log-scale topic-bias strength	$\mathcal{N}(0,\, 0.5)$
$\log \beta_{\text{top,sd}}$	Hyperprior standard deviation of the log-scale topic-bias strength	HalfNormal$(\sigma = 0.3)$
$\log \beta_{\text{top},k}$	Topic-specific log-scale topic-bias strength	$\log \bar{\beta}_{\text{top}} + \log \beta_{\text{top,sd}} \cdot z_{\beta_{\text{top}},k},\;\; z_{\beta_{\text{top}},k} \sim \mathcal{N}(0, 1)$
$\beta_{\text{top},k}$	Topic-specific topic-bias strength	softplus$(4 \log \beta_{\text{top},k})/4$
$\log \lambda_{\text{top}}$	Log-scale temporal decay rate of the topic-bias term	$\mathcal{N}(0,\, 1)$
λ_{top}	Temporal decay rate of the topic-bias term	$\exp(\log \lambda_{\text{top}})$
$b_{\text{top},k}$	Topic-specific attractor position in the rescaled opinion space	$\mathcal{U}(-1,\, 1)$
$\bar{\beta}_{\text{agr}}$	Hyperprior mean of the topic-specific agreement-bias strength	$\mathcal{N}(0,\, 0.1)$
$\beta_{\text{agr,sd}}$	Hyperprior standard deviation of the topic-specific agreement-bias strength	HalfNormal$(\sigma = 0.1)$
$\beta_{\text{agr},k}$	Topic-specific agreement-bias strength	$\bar{\beta}_{\text{agr}} + \beta_{\text{agr,sd}} \cdot z_{\beta_{\text{agr}},k},\quad z_{\beta_{\text{agr}},k} \sim \mathcal{N}(0, 1)$
$\log \lambda_{\text{agr}}$	Log-scale temporal decay rate of the agreement-bias term	$\mathcal{N}(0,\, 1)$
λ_{agr}	Temporal decay rate of the agreement-bias term	$\exp(\log \lambda_{\text{agr}})$
$\bar{\beta}_{\text{anc}}$	Hyperprior mean of the topic-specific anchoring-bias strength	$\mathcal{N}(0,\, 0.1)$
$\beta_{\text{anc,sd}}$	Hyperprior standard deviation of the topic-specific anchoring-bias strength	HalfNormal$(\sigma = 0.1)$
$\beta_{\text{anc},k}$	Topic-specific anchoring-bias strength	$\bar{\beta}_{\text{anc}} + \beta_{\text{anc,sd}} \cdot z_{\beta_{\text{anc}},k},\quad z_{\beta_{\text{anc}},k} \sim \mathcal{N}(0, 1)$
$\log \lambda_{\text{anc}}$	Log-scale temporal decay rate of the anchoring-bias term	$\mathcal{N}(0,\, 1)$
λ_{anc}	Temporal decay rate of the anchoring-bias term	$\exp(\log \lambda_{\text{anc}})$
$\log \bar{\sigma}_0$	Hyperprior mean of the topic-specific log-scale baseline noise	$\mathcal{N}(\log 0.1,\, 1)$
$\log \sigma_{0,\text{sd}}$	Hyperprior standard deviation of the topic-specific log-scale baseline noise	HalfNormal$(\sigma = 0.3)$
$\log \sigma_{0,k}$	Topic-specific log-scale baseline noise	$\log \bar{\sigma}_0 + \log \sigma_{0,\text{sd}} \cdot z_{\sigma_0,k},\quad z_{\sigma_0,k} \sim \mathcal{N}(0, 1)$
$\sigma_{0,k}$	Topic-specific baseline noise scale	softplus$(\log \sigma_{0,k})$
$\log \varepsilon$	Log-scale coefficient of the entropy-dependent noise term	$\mathcal{N}(\log 0.1,\, 1)$
ε	Coefficient of the entropy-dependent noise term	softplus$(\log \varepsilon)$

Ablation Studies

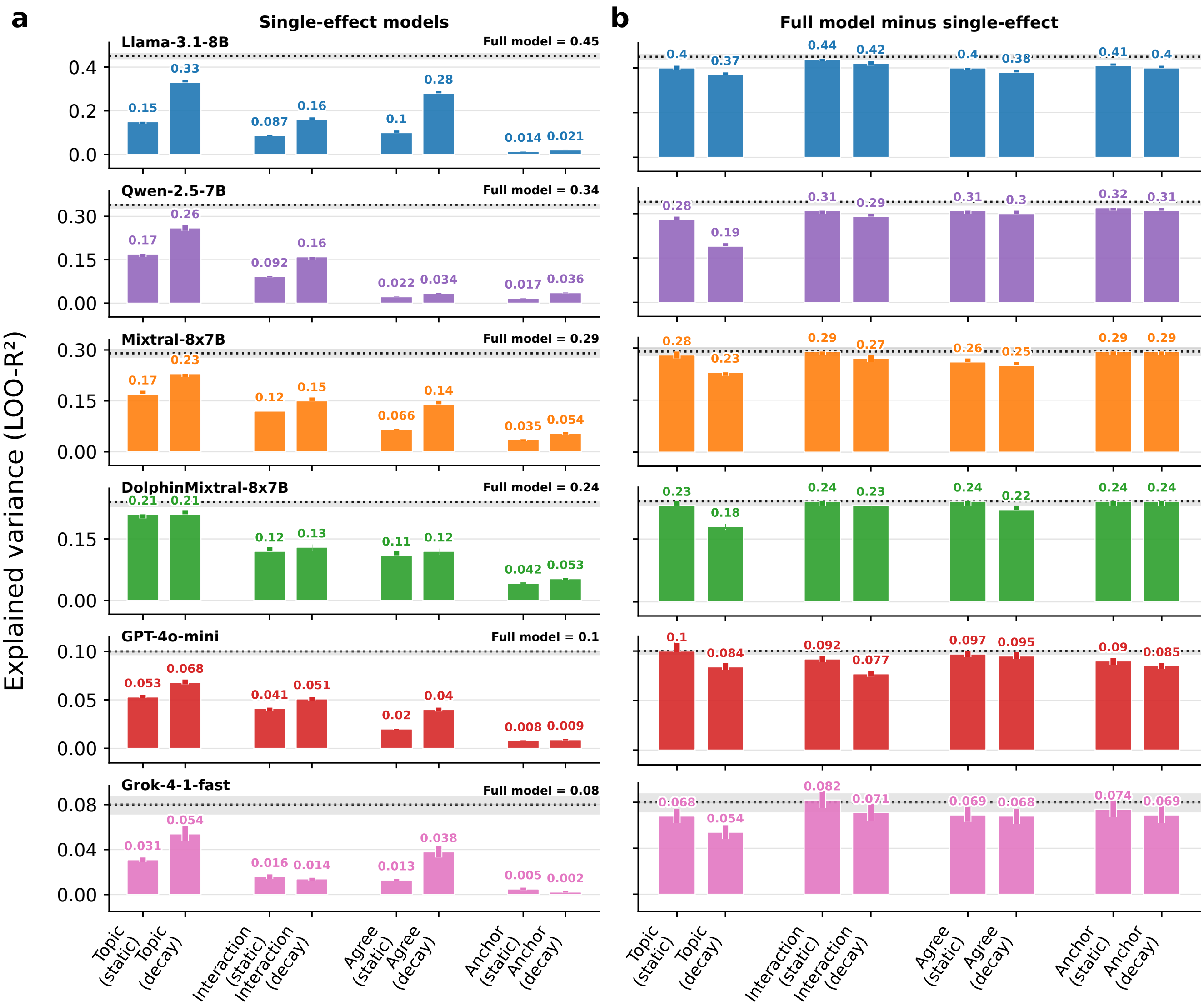

Supplementary Figure 2: **Leave-one-out explained variance (LOO-R^2) values of the Bayesian model for all six LLMs in two complementary ablation analyses reveal the topic bias as the effect with most individual explanatory power.** (**a**) Single-effect models containing only one effect, either without (static) or with (decay) a fitted exponential time decay, and (**b**) full-model ablations in which one effect is removed at a time. Black dotted horizontal lines indicate the performance of the corresponding full model. The LOO-R^2 error bars indicate 95% equal-tailed intervals, translucent horizontal bands for explainable variance show its 95% confidence interval.

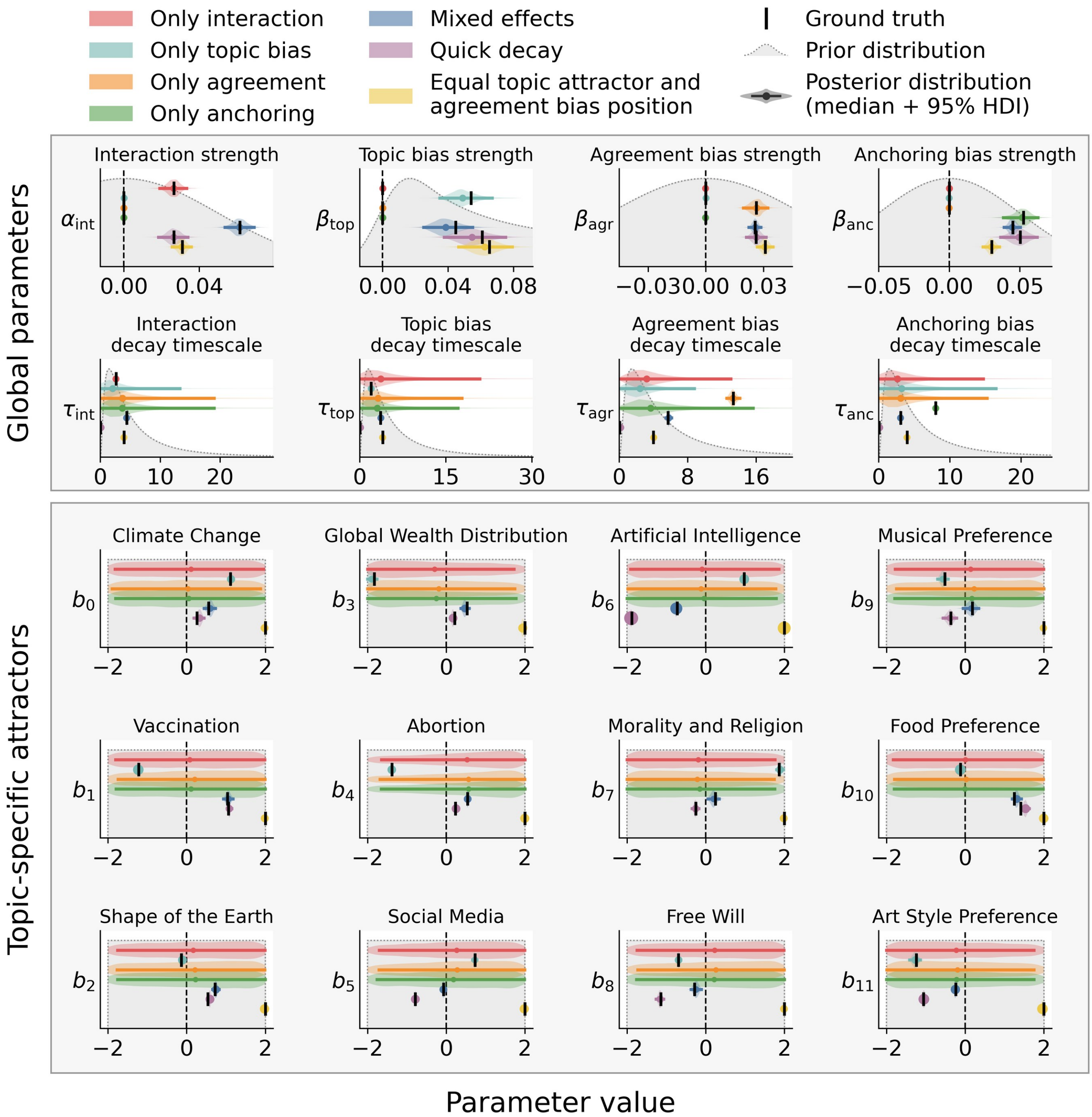

Supplementary Figure 3: **Our Bayesian model can successfully recover known ground-truth parameter values from synthetic data across different scenarios.** In the absence of an effect, our model constrains the respective effect strength $\alpha_{\text{int}}, \beta_\bullet$ around zero, while correctly leaving the timescale constant $\tau_\bullet$ and, in the case of the topic bias, the topic attractor positions b_k unconstrained as expected.

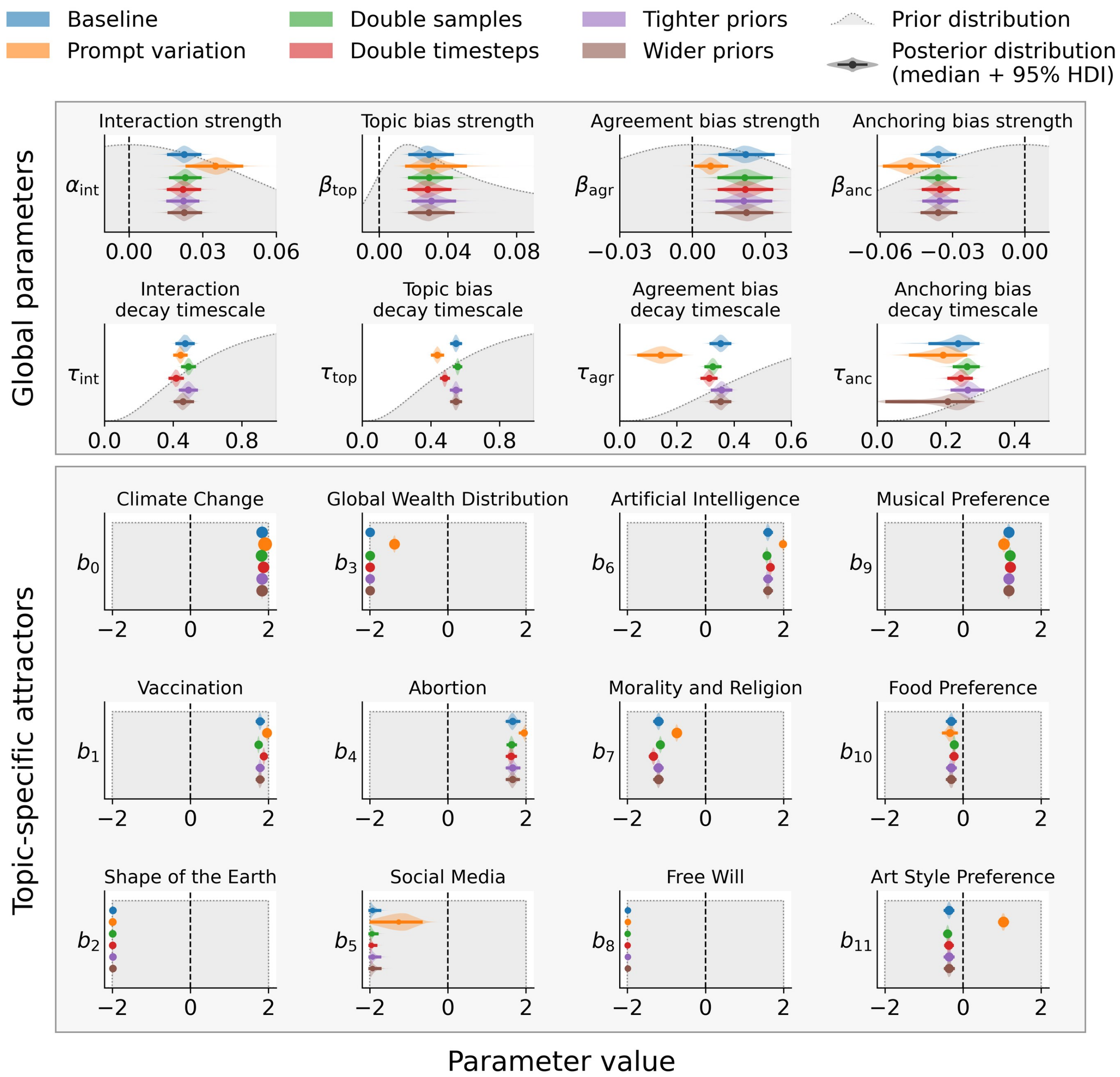

Supplementary Figure 4: **The Bayesian inference remains stable within minor deviations to the baseline results for twice as many samples per initial condition, twice as many discussions steps, tighter priors, wider priors and prompt variations in the opinion probing.** Robustness checks conducted with the Llama-3.1-8B LLM. In the opinion probing we changed "Only return the corresponding integer value without any comments or punctuation." to "Answer with a single number from 1 to 5 without any comments or punctuation.". For the prior variations, we doubled / halved the standard deviation of all population-level and individual-level parameters. The uniform distribution for $b_{\text{top},k}$ remains unchanged as its values are inherently constrained by the data.

Effect Sizes of Residual Terms

For the scale parameters $\sigma_{0,k}$ and ε, we do not compute standardized effect sizes, since they do not have an independent variable required in the definition. Instead, we quantify their effect size as *direct* contribution to the residual standard deviation $\sigma_i(t)$, since this is the relevant quantity for assessing the absolute magnitude of the noise terms. All expectations below are taken over the pooled observations used for inference. For posterior draw s, we define the time-averaged effect size of the topic-specific baseline standard deviation as

$$\mathrm{E}_{\mathrm{direct}}\left(\sigma_0^{(s)}\right) = \mathbb{E}_{i,k,t}\left(\sigma_{0,k}^{(s)}\right), \tag{7}$$

that is, $\sigma_{0,k}^{(s)}$ averaged over the empirical topic mixture of all pooled observations. Analogously, the time-averaged effect size of the entropy-dependent scale term is

$$\mathrm{E}_{\mathrm{direct}}\left(\varepsilon^{(s)}\right) = \varepsilon^{(s)} \cdot \mathbb{E}_{i,k,t}\left(H_i^{(k)}(t)\right). \tag{8}$$

To resolve how these scale contributions change over the course of a discussion, we additionally compute time-dependent effect size by restricting the expectation to a fixed discussion step t^*:

$$\mathrm{E}_{\mathrm{direct}}^{(t^*)}\left(\sigma_0^{(s)}\right) = \mathbb{E}_{i,k}\left(\sigma_{0,k}^{(s)} \mid t = t^*\right), \tag{9}$$

and

$$\mathrm{E}_{\mathrm{direct}}^{(t^*)}\left(\varepsilon^{(s)}\right) = \varepsilon^{(s)} \cdot \mathbb{E}_{i,k}\left(H_i^{(k)}(t^*)\right). \tag{10}$$

In contrast to the standardized effect sizes used for the interaction and bias terms, these direct effect sizes are not normalized by the standard deviation of the observed opinion shifts. They therefore represent the absolute additive contribution of each term to the residual scale itself. We report posterior medians and 95% HDIs of these effect sizes (see Supplementary Fig. 6.)

Theoretical Bounds on Opinion Entropy

To understand the entropy of the LLM's response distribution as a measure for the uncertainty of an LLM agent in its opinion, it is instructive to consider the minimum and maximum values this quantity can take on for any given expected opinion.

The Shannon entropy of the output distribution is defined as [7]

$$H = \sum_s p(s) \log_2 \frac{1}{p(s)}, \tag{11}$$

where $p(s)$ denote the probability mass of each opinion option step s on the integer scale from -2 to 2.

For any given expected opinion $x = \sum_s s\, p(s)$, the entropy takes on its minimum value if only the two closest integer opinions $\lfloor x \rfloor$ and $\lceil x \rceil$ have non-zero probabilities. The entropy H is then equal to that of a biased coin flip given by [7]

$$H = -p(\lfloor x \rfloor) \log_2 p(\lfloor x \rfloor) - (1 - p(\lfloor x \rfloor)) \log_2 (1 - p(\lfloor x \rfloor)). \tag{12}$$

Note that this minimum entropy becomes zero when x is equal to one of the levels s, as all probability gets concentrated on a single step $p(s)$ and no uncertainty about the answer remains.

The entropy takes on its maximum value for a maximally uniform distribution under the constraint given by the fixed expectation value x. This problem can be formulated as a constrained optimization of H under the conditions that $\sum_s p(s) = 1$ and $\sum_s s\, p(s) = x$, which results in the Lagrangian

$$\mathcal{L} = \sum_s p(s) \log_2 \frac{1}{p(s)} + \lambda_1 \left(\sum_s p(s) - 1\right) + \lambda_2 \left(\sum_s s\, p(s) - x\right). \tag{13}$$

Differentiating with respect to $p(s)$ and rearranging results in the Gibbs distribution

$$p(s) = \frac{e^{-\lambda_2 s}}{Z(\lambda_2)}, \tag{14}$$

where the partition function

$$Z(\lambda_2) = \sum_s e^{-\lambda_2 s} \tag{15}$$

ensures normalization. For any given expectation value x, the Lagrange multiplier λ_2 can be found numerically through solving the equation

$$x = \frac{1}{Z} \sum_s s\, e^{-\lambda_2 s}. \tag{16}$$

Finally, the entropy is obtained by plugging the resulting probability mass function $p(s)$ into Supplementary Eq. (11). This maximum entropy is largest for $x = 0$ and vanishes at the extremes for $x = -2$ and $x = 2$.

Fine-tuning Details

We created five custom fine-tuned instances of an LLM using parameter-efficient fine-tuning (PEFT) via Low-Rank Adaptation (LoRA) [8]. The base model was `Mixtral-8x7B-Instruct-v0.1`. The procedure employed the transformers, datasets and PEFT libraries from HuggingFace, using PyTorch on a single NVIDIA A100 GPU.

Dataset Preparation The fine-tuning dataset consists of 30,000 messages about climate change from the platform "Telegram", that were filtered using BERTopic. Subsequently, we classified the opinion of each message according to the five-point query scale by the GPT-4o-mini LLM, selecting the 725 messages nearest to each integer opinion level. The dataset was split into training and evaluation subsets with a 90/10 ratio.

Instruction Formatting and Tokenization Each message was prepended with a randomly selected user instruction prompt from a curated list of 10 templates, simulating user-assistant chat interactions to make the LLM learn a connection between the desired prompted opinion and its output. We used a maximum sequence length of 1024 tokens with truncation and padding to fixed length (sufficient for all messages).

LoRA Configuration We used PEFT via LoRA to enable efficient fine-tuning. The LoRA configuration was as follows:

- Rank: $r = 8$
- Scaling factor: $\alpha = 16$
- Dropout: 0.1
- Target modules: `["w1", "w2", "w3"]`

Before applying LoRA, the model was prepared for k-bit training, as quantization was used to reduce memory overhead. The w1, w2, and w3 layers correspond to the expert feedforward layers in each Mixture of Experts block of the Mixtral LLM. Within our setup, 0.25% of the total 46.8 billion parameters were trainable.

Training Configuration Fine-tuning was performed using the HuggingFace `Trainer` with the following settings:

- Epochs: 5
- Per-device batch size: 4
- Gradient accumulation steps: 2
- Effective batch size: 8
- Learning rate: 10^{-4}
- Evaluation every 50 steps
- Checkpoint saving every 50 steps (keeping the last 3)
- Early stopping with patience of 3 evaluation cycles
- Mixed-precision (FP16) training
- Gradient checkpointing enabled with `use_reentrant = False`

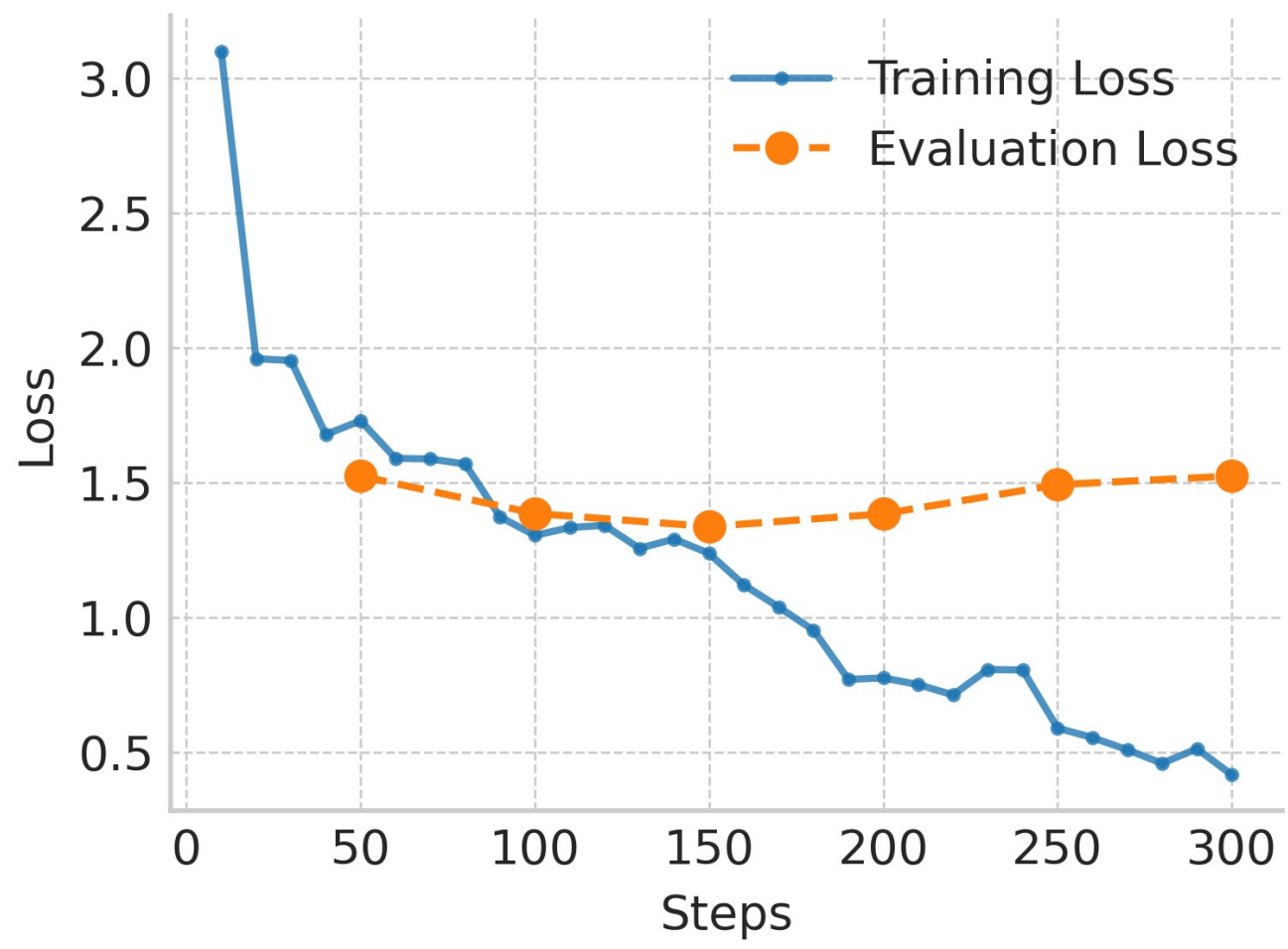

Supplementary Figure 5: **Example loss curve for one of our fine-tuned checkpoints.** All five fine-tuned LLMs resulted in having the lowest evaluation loss after 150 steps.

Evaluation For subsequent use, we selected the checkpoints based on minimum evaluation loss, which corresponded to 150 steps (see. Supplementary Fig. 5).

Additional Supplementary Figures and Tables

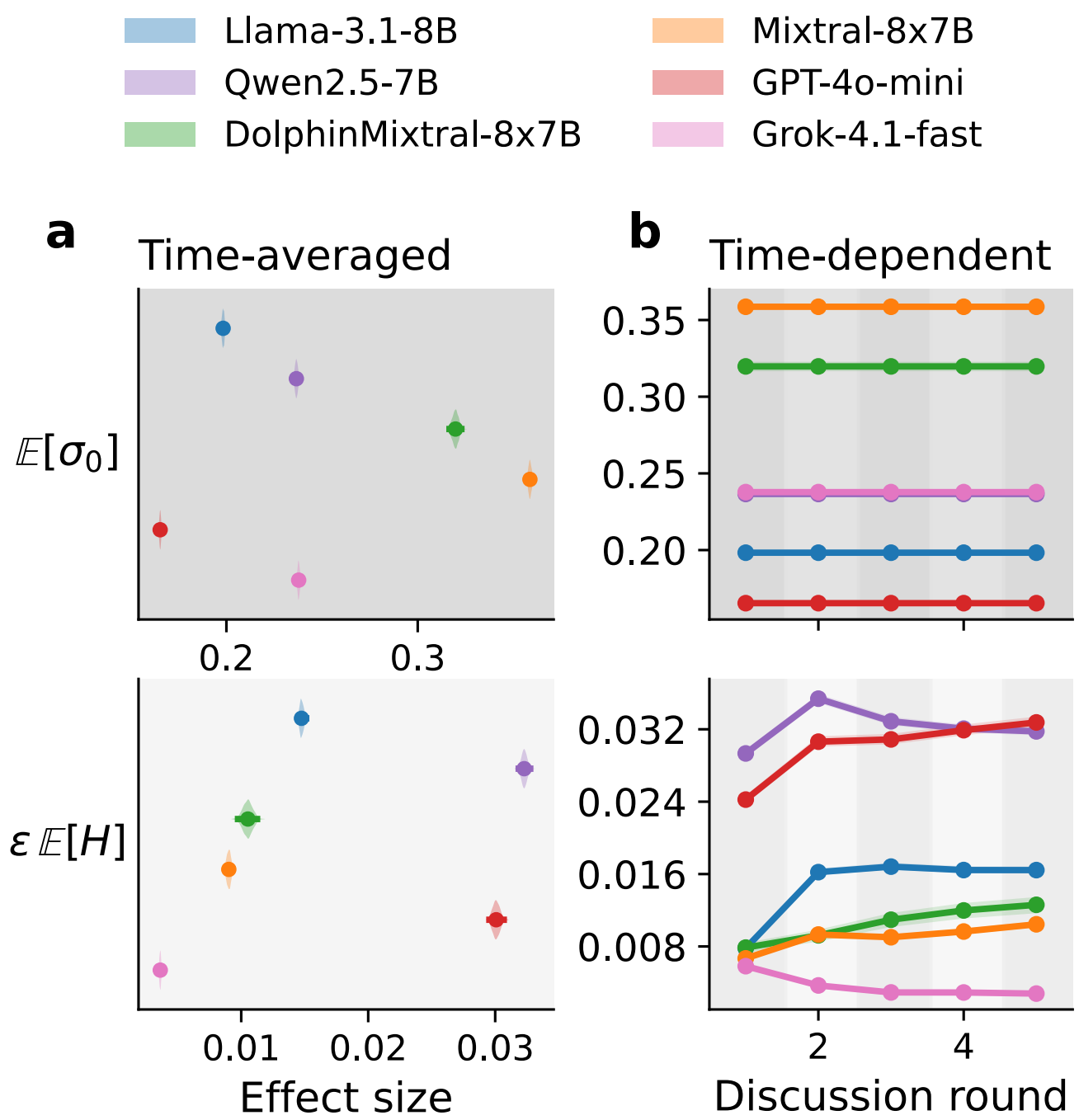

Supplementary Figure 6: **For all LLMs, most of residual variance in the Bayesian model results from the baseline standard deviation term, whereas the entropy-dependent contribution is one order of magnitude smaller but significant. (a)** Time-averaged direct effect sizes and **(b)** time-dependent direct effect sizes.

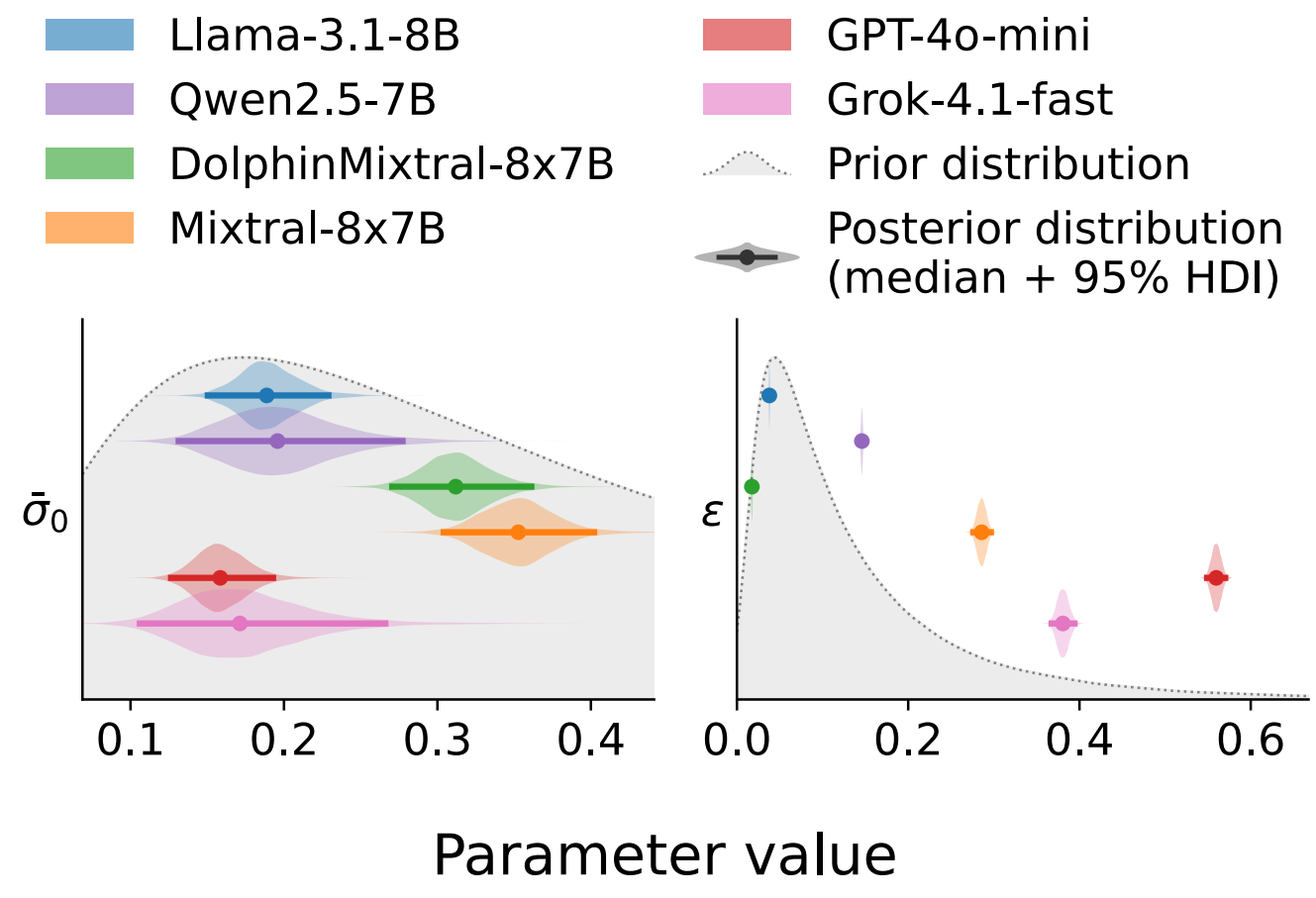

Supplementary Figure 7: **Inferred posterior distributions (median + 95% HDI) of parameters contributing to the standard deviation of our Bayesian model for the different LLMs.** For exact prior and posterior values, refer to Supplementary Table 1 and Supplementary Table 5.

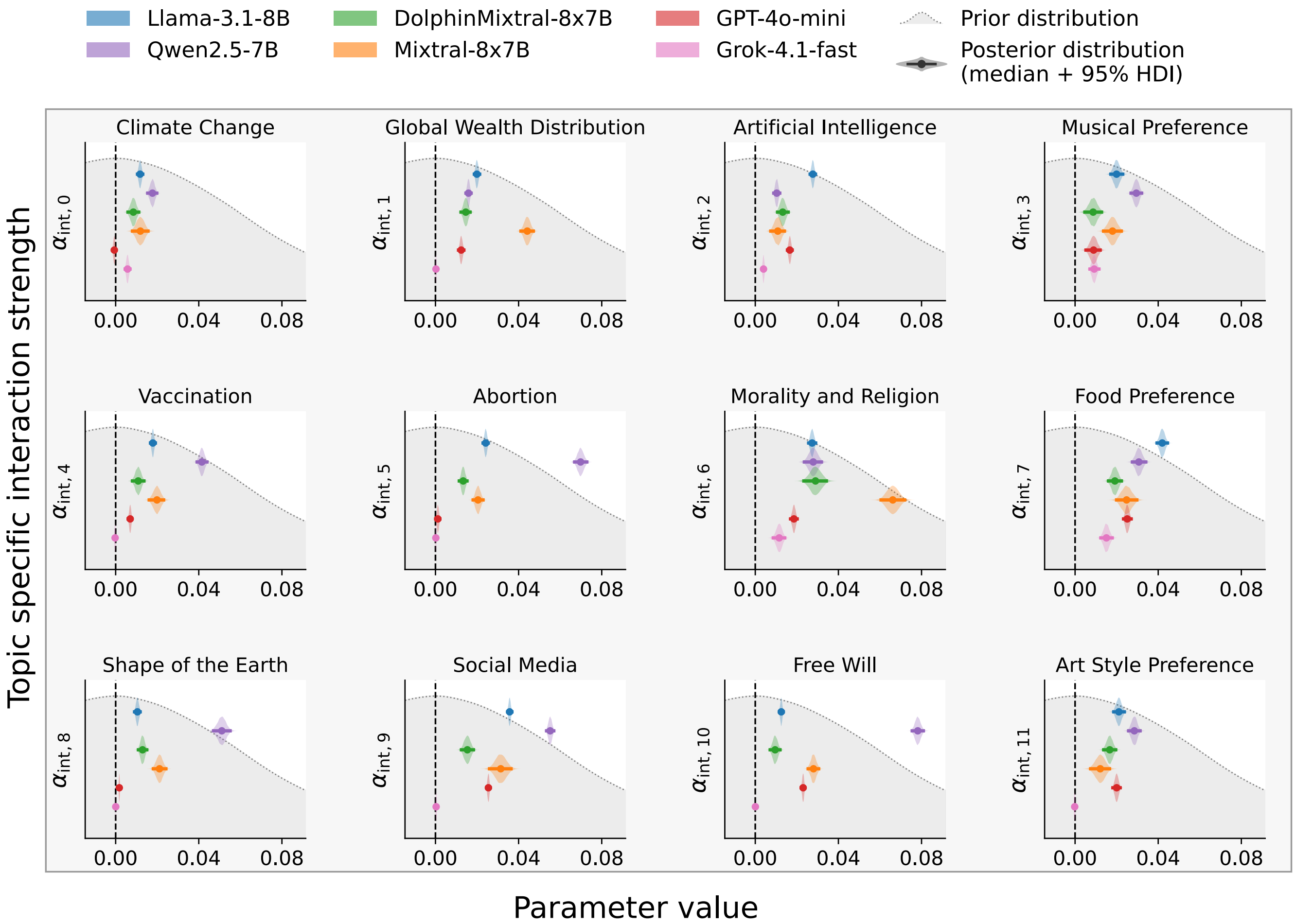

Supplementary Figure 8: **Inferred posterior distributions (median + 95% HDI) of topic-specific interaction strength $\alpha_{\text{int},k}$ for all six LLMs.** For exact prior and posterior values, refer to Supplementary Table 6.

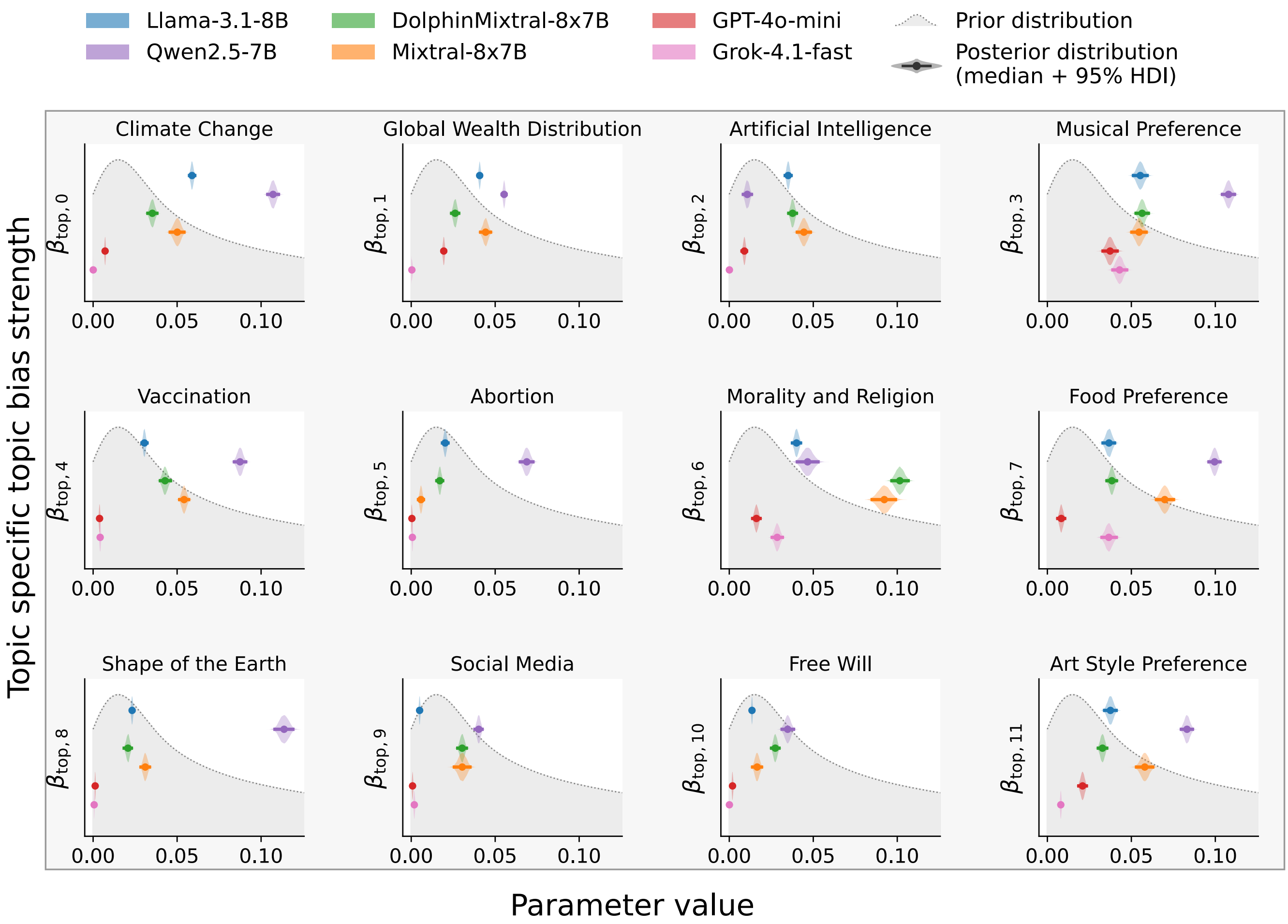

Supplementary Figure 9: **Inferred posterior distributions (median + 95% HDI) of topic-specific topic-bias strength $\beta_{\mathrm{top},k}$ for all six LLMs.** For exact prior and posterior values, refer to Supplementary Table 7.

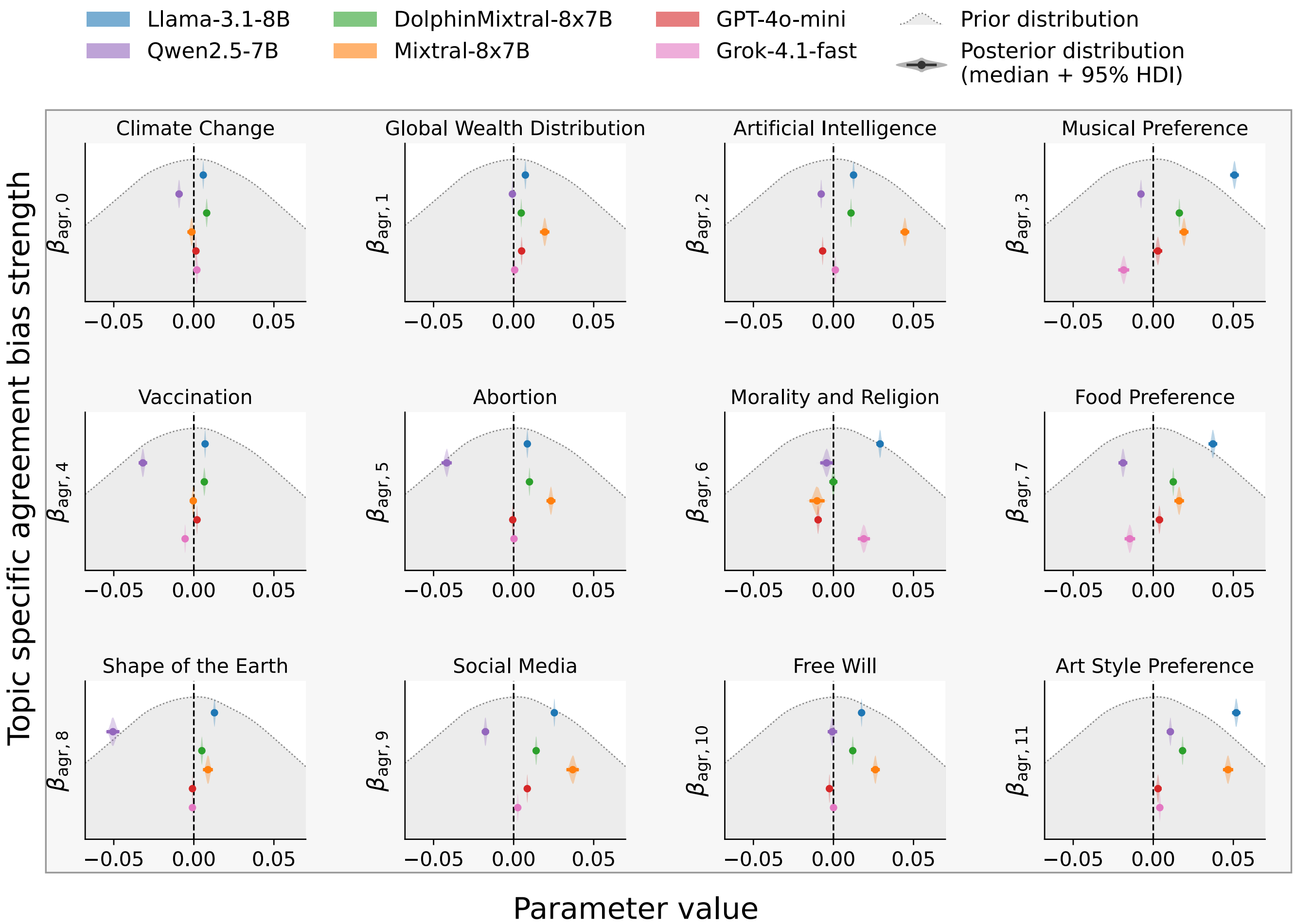

Supplementary Figure 10: **Inferred posterior distributions (median + 95% HDI) of topic-specific agreement-bias strength $\beta_{\mathrm{agr},k}$ for all six LLMs.** For exact prior and posterior values, refer to Supplementary Table 8.

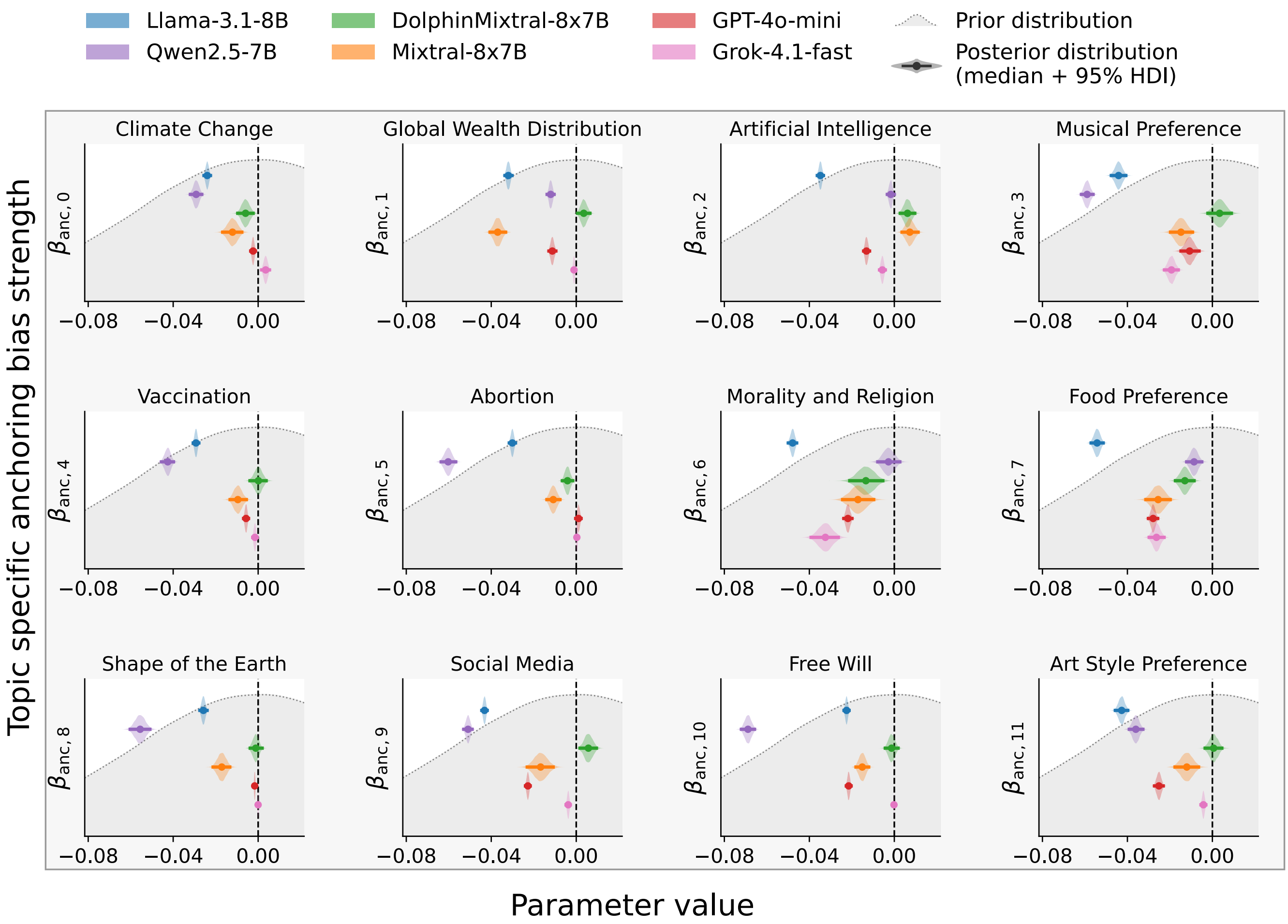

Supplementary Figure 11: **Inferred posterior distributions (median + 95% HDI) of topic-specific anchoring-bias strength $\beta_{\mathrm{anc},k}$ for all six LLMs.** For exact prior and posterior values, refer to Supplementary Table 9.

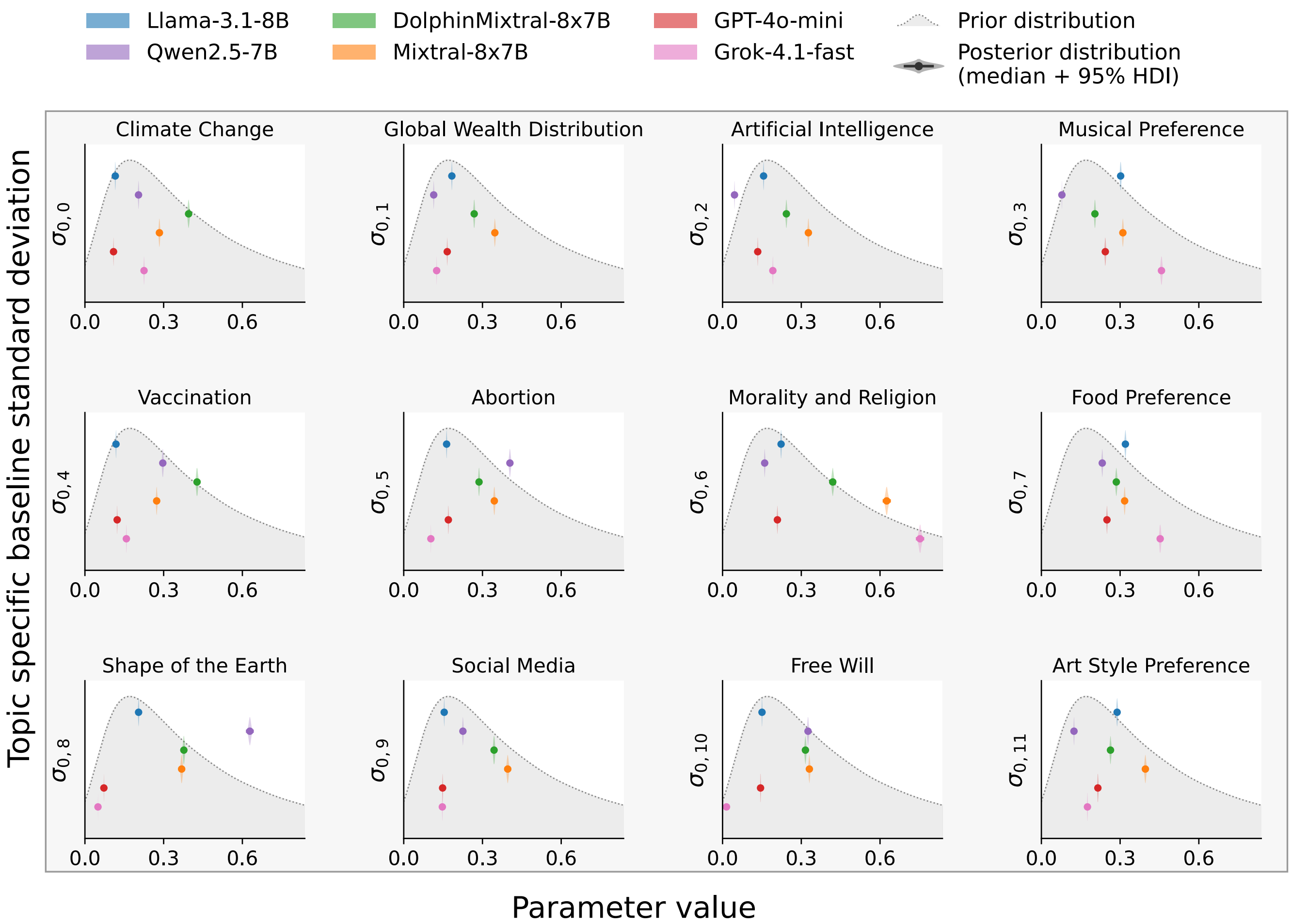

Supplementary Figure 12: **Inferred posterior distributions (median + 95% HDI) of topic-specific baseline standard deviation $\sigma_{0,k}$ for all six LLMs.** For exact prior and posterior values, refer to Supplementary Table 10.

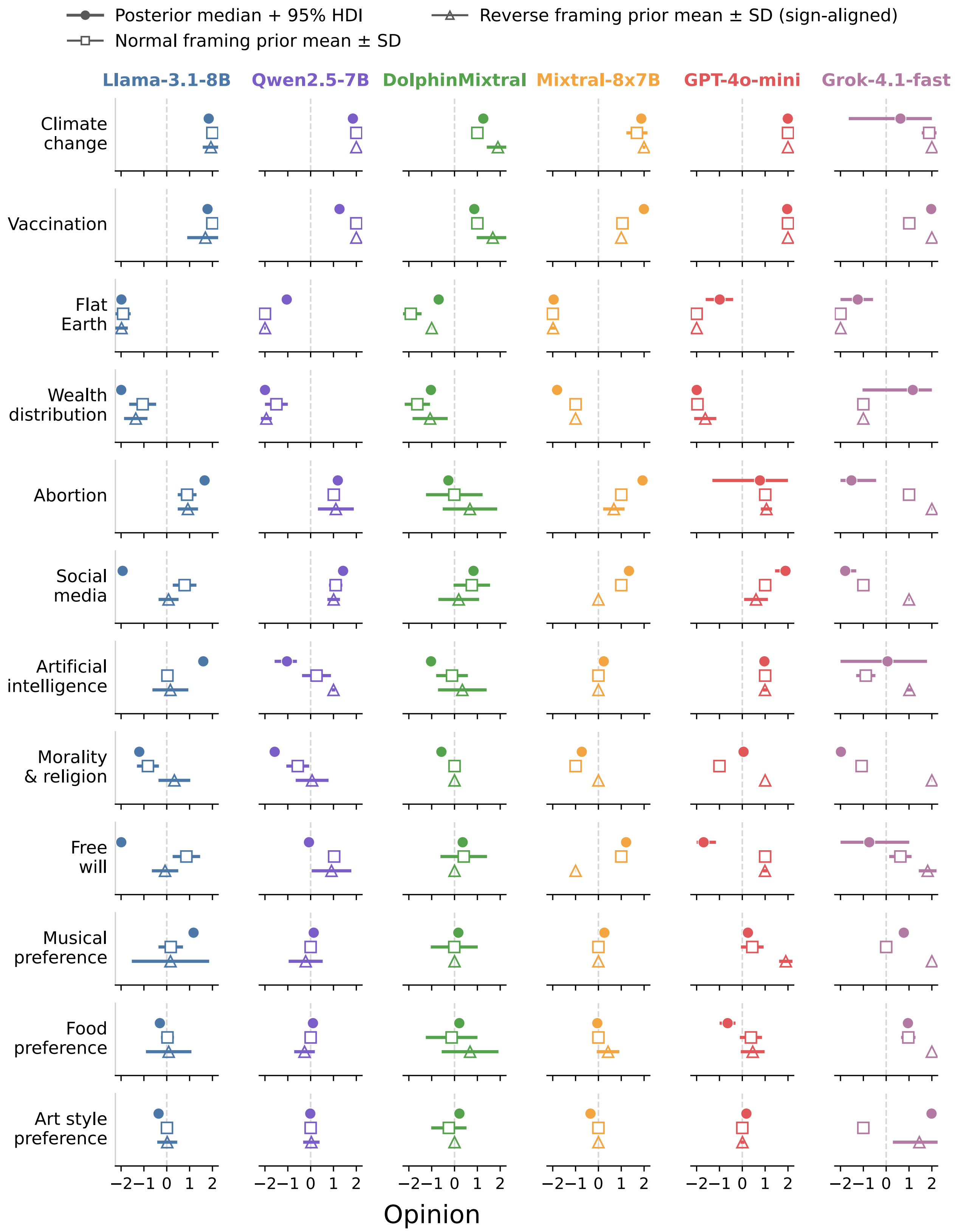

Supplementary Figure 13: **The topic bias attractor and the LLM's prior opinion about a statement without any system prompt or persona initialization, exhibit some overlap. Still, inferring the attractor position, results in a more accurate estimation.**

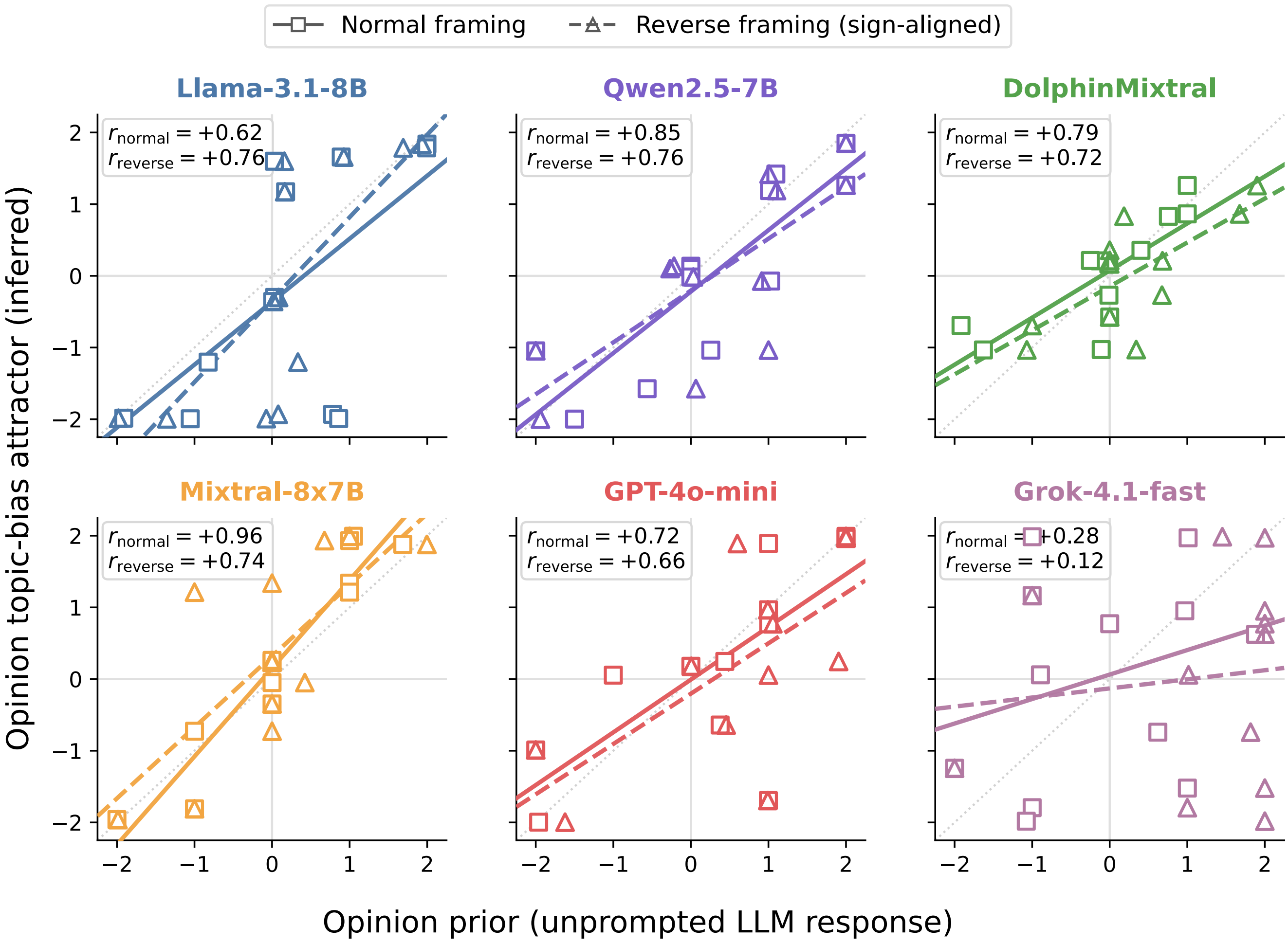

Supplementary Figure 14: **The inferred topic bias attractor and the LLM's prior opinion about a statement without any system prompt or persona initialization are positively correlated.**

Supplementary Table 2: **Topic priors of statements and logically negated statements, with response entropies H.** Each table entry shows the expected opinion for the normal framing and, in parentheses, the expected opinion for the reverse framing. The second line shows the corresponding entropy values in the same order. We obtain these values by asking an uninitialized LLM (having empty context) the opinion probing prompt (see Supplementary Methods: Simulation Details and Prompting).

	**Llama-3.1-8B**	**Qwen2.5-7B**	**Dolphin-Mixtral 8x7B**	**Mixtral-8x7B**	**GPT-4o-mini**	**Grok-4.1-fast**
Climate change is (not) an important problem to address.	2.00 (−1.93)<br>H: 0.03 (0.32)	2.00 (−2.00)<br>H: 0.00 (0.00)	1.00 (−1.90)<br>H: 0.00 (0.40)	1.69 (−2.00)<br>H: 0.90 (0.03)	2.00 (−2.00)<br>H: 0.00 (0.00)	1.88 (−2.00)<br>H: 0.53 (0.00)
Vaccinations are (not) necessary for public health.	1.99 (−1.69)<br>H: 0.05 (0.91)	2.00 (−2.00)<br>H: 0.02 (0.00)	1.00 (−1.67)<br>H: 0.00 (1.01)	1.05 (−1.00)<br>H: 0.29 (0.00)	2.00 (−2.00)<br>H: 0.00 (0.00)	1.01 (−2.00)<br>H: 0.09 (0.00)
The Earth is (not) flat.	−1.91 (1.98)<br>H: 0.43 (0.06)	−2.00 (2.00)<br>H: 0.00 (0.00)	−1.92 (1.00)<br>H: 0.32 (0.00)	−2.00 (1.99)<br>H: 0.00 (0.10)	−2.00 (2.00)<br>H: 0.00 (0.00)	−2.00 (2.00)<br>H: 0.00 (0.00)
Global wealth distribution is (not) fair.	−1.05 (1.36)<br>H: 1.03 (1.06)	−1.50 (1.94)<br>H: 1.00 (0.33)	−1.63 (1.07)<br>H: 1.06 (1.30)	−1.00 (1.00)<br>H: 0.00 (0.00)	−1.96 (1.62)<br>H: 0.23 (0.96)	−1.00 (1.00)<br>H: 0.00 (0.00)
Abortions should (not) be legal.	0.89 (−0.93)<br>H: 0.64 (0.89)	1.01 (−1.10)<br>H: 0.12 (1.43)	−0.01 (−0.67)<br>H: 2.25 (2.08)	1.00 (−0.68)<br>H: 0.00 (0.91)	1.00 (−1.06)<br>H: 0.03 (0.36)	1.00 (−2.00)<br>H: 0.01 (0.00)
Social media does (not) have a positive societal impact.	0.78 (−0.08)<br>H: 1.10 (0.84)	1.10 (−1.01)<br>H: 0.45 (0.40)	0.76 (−0.18)<br>H: 1.54 (1.78)	1.00 (0.00)<br>H: 0.00 (0.00)	1.00 (−0.60)<br>H: 0.00 (1.05)	−1.00 (−1.00)<br>H: 0.00 (0.01)
Artificial intelligence is (not) dangerous.	0.03 (−0.16)<br>H: 0.42 (1.63)	0.26 (−1.00)<br>H: 1.38 (0.06)	−0.11 (−0.34)<br>H: 1.40 (2.08)	0.00 (0.00)<br>H: 0.00 (0.00)	1.00 (−0.99)<br>H: 0.00 (0.07)	−0.89 (−1.02)<br>H: 0.41 (0.12)
One can(not) only be moral if one believes in God.	−0.82 (−0.33)<br>H: 0.89 (1.48)	−0.56 (−0.06)<br>H: 1.01 (1.51)	0.00 (0.00)<br>H: 0.00 (0.00)	−1.00 (0.00)<br>H: 0.00 (0.00)	−1.00 (−1.00)<br>H: 0.00 (0.00)	−1.08 (−2.00)<br>H: 0.39 (0.00)
Humans do (not) possess free will.	0.86 (0.07)<br>H: 1.31 (1.19)	1.03 (−0.91)<br>H: 0.19 (1.72)	0.40 (0.00)<br>H: 2.00 (0.00)	1.00 (1.00)<br>H: 0.00 (0.00)	1.00 (−0.99)<br>H: 0.01 (0.08)	0.62 (−1.82)<br>H: 0.97 (0.69)
Bach is (not) greater than Stravinsky.	0.17 (−0.16)<br>H: 1.04 (2.10)	−0.00 (0.22)<br>H: 0.01 (1.53)	−0.01 (0.00)<br>H: 2.01 (0.00)	0.00 (−0.00)<br>H: 0.00 (0.00)	0.44 (−1.90)<br>H: 0.99 (0.45)	0.00 (−2.00)<br>H: 0.00 (0.00)
Pizza is (not) better than sushi.	0.03 (−0.08)<br>H: 0.29 (1.79)	−0.00 (0.27)<br>H: 0.00 (0.88)	−0.13 (−0.68)<br>H: 2.17 (2.13)	0.00 (−0.42)<br>H: 0.00 (0.98)	0.38 (−0.46)<br>H: 0.96 (1.07)	0.97 (−2.00)<br>H: 0.10 (0.00)
Modern art is (not) more meaningful than classical art.	0.01 (−0.02)<br>H: 0.26 (0.81)	−0.00 (−0.03)<br>H: 0.00 (0.66)	−0.25 (0.00)<br>H: 1.59 (0.00)	0.00 (0.00)<br>H: 0.00 (0.00)	0.00 (−0.01)<br>H: 0.00 (0.07)	−1.00 (−1.45)<br>H: 0.00 (0.69)

Supplementary Table 3: **Linear regression statistics for relating opinion uncertainty to the variance in opinion shift for each LLM.** Regressions are fit to the binned points shown in Fig. 8.

Model	n_{bins}	Slope	Intercept	r	p
Llama-3.1-8B	10	0.0275	0.0636	0.376	0.285
Qwen2.5-7B	10	0.1943	0.0759	0.949	2.78×10^{-5}
DolphinMixtral-8x7B	10	0.0704	0.0553	0.608	0.0623
Mixtral-8x7B	7	0.6522	0.0428	0.868	0.0114
GPT-4o-mini	7	0.3495	0.0305	0.939	0.00174
Grok-4.1-fast	7	0.5223	0.1347	0.924	0.00293

Supplementary Table 4: **Publicly reported metadata, benchmarks, and public arena rankings for the LLMs used in this study.** Publicly reported benchmarks and public arena rankings suggest that GPT-4o mini and Grok LLM lie toward the higher-capability end of our model set, although benchmark coverage is incomplete and some entries correspond to closely related public variants rather than the exact deployed checkpoints. Exact model-specific values are shown where available; proxy values are explicitly marked.

**Benchmark / Metadata**	**Llama-3.1 8B-Instruct**	**Qwen2.5 7B-Instruct**	**dolphin-2.7 mixtral-8x7b AWQ-INT4**	**Mixtral-8x7B Instruct-v0.1 AWQ-INT4**	**gpt-4o-mini**	**grok-4-1-fast non-reasoning**
Release date	2024-07-23	2024-09-19	2024-01-01	2024-10-05	2024-07-18	2025-11-17
Knowledge cutoff	Dec 2023	–	–	–	Oct 2023	Nov 2024[§]
Parameters (total / active)	8B / 8B	7.61B / 7.61B	47B / 13B[†]	47B / 13B	undisclosed	undisclosed
MMLU (or closest)[a]	69.4	56.3 / 75.4	–	70.6[†]	82.0	–
GSM8K / MGSM[b]	84.5	91.6	–	74.4[†]	87.0	–
HumanEval[c]	72.6	84.8	–	40.2[†]	87.2	–
MATH[d]	51.9	75.5	–	28.4[†]	–	–
Text Arena Overall[e]	1186 ± 4	–	–	1131 ± 4[‡]	1286 ± 4	1408 ± 8[⋆]

Sources by model: Llama-3.1-8B-Instruct [9]; Qwen2.5-7B-Instruct [10]; dolphin-2.7-mixtral-8x7b-AWQ-INT4 [11, 12]; Mixtral-8x7B-Instruct-v0.1-AWQ-INT4 [13–15]; GPT-4o mini [16, 17]; Grok-4.1 / grok-4-1-fast-non-reasoning [18–20]. Text Arena values were read from the public Text Arena Overall leaderboard [21].

[a] This row is not fully harmonized across models. Llama-3.1-8B-Instruct reports standard MMLU. Qwen2.5-7B-Instruct reports MMLU-Pro / MMLU-redux rather than a single standard MMLU score. GPT-4o mini reports standard MMLU. No exact public MMLU-style value was found for dolphin-2.7-mixtral-8x7b-AWQ or grok-4-1-fast-non-reasoning.
[b] This row mixes GSM8K and MGSM, so values are only approximately comparable. Llama and Qwen report GSM8K; GPT-4o mini reports MGSM. No exact public value was found for Dolphin or Grok Fast.
[c] HumanEval is directly comparable where available. No exact public HumanEval figure was found for dolphin-2.7-mixtral-8x7b-AWQ-INT4 or grok-4-1-fast-non-reasoning.
[d] GPT-4o mini does not report a MATH value on the cited release page. No exact public MATH value was found for Dolphin or Grok Fast.
[e] Arena scores are dynamic; values shown here were read from the public Text Arena Overall leaderboard accessed on 2026-04-08.
[†] Family proxy from the Mixtral 8x7B base-model evaluation table in Jiang et al. [15], not from the exact deployed instruct/AWQ checkpoint. For dolphin-2.7-mixtral-8x7b-AWQ-INT4, the parameter count is inherited from the underlying Mixtral 8x7B architecture rather than separately benchmarked for the AWQ checkpoint.
[‡] The Arena value corresponds to the public `mixtral-8x7b-instruct-v0.1` entry, not explicitly to the AWQ-INT4 quantized checkpoint.
[⋆] Proxy from the closest public Arena entry, `grok-4-fast-chat`; no exact public Text Arena entry was found for `grok-4-1-fast-non-reasoning`.
[§] xAI publicly states a knowledge cutoff of November 2024 for Grok 3 / Grok 4 generally; the checked public sources do not give a separate cutoff for the exact `grok-4-1-fast-non-reasoning` variant.
For the two quantized checkpoints, the release date denotes the first publicly verifiable checkpoint / commit date for the exact repository used as source.
"–" denotes that no exact public value was found for the specified deployed model variant.

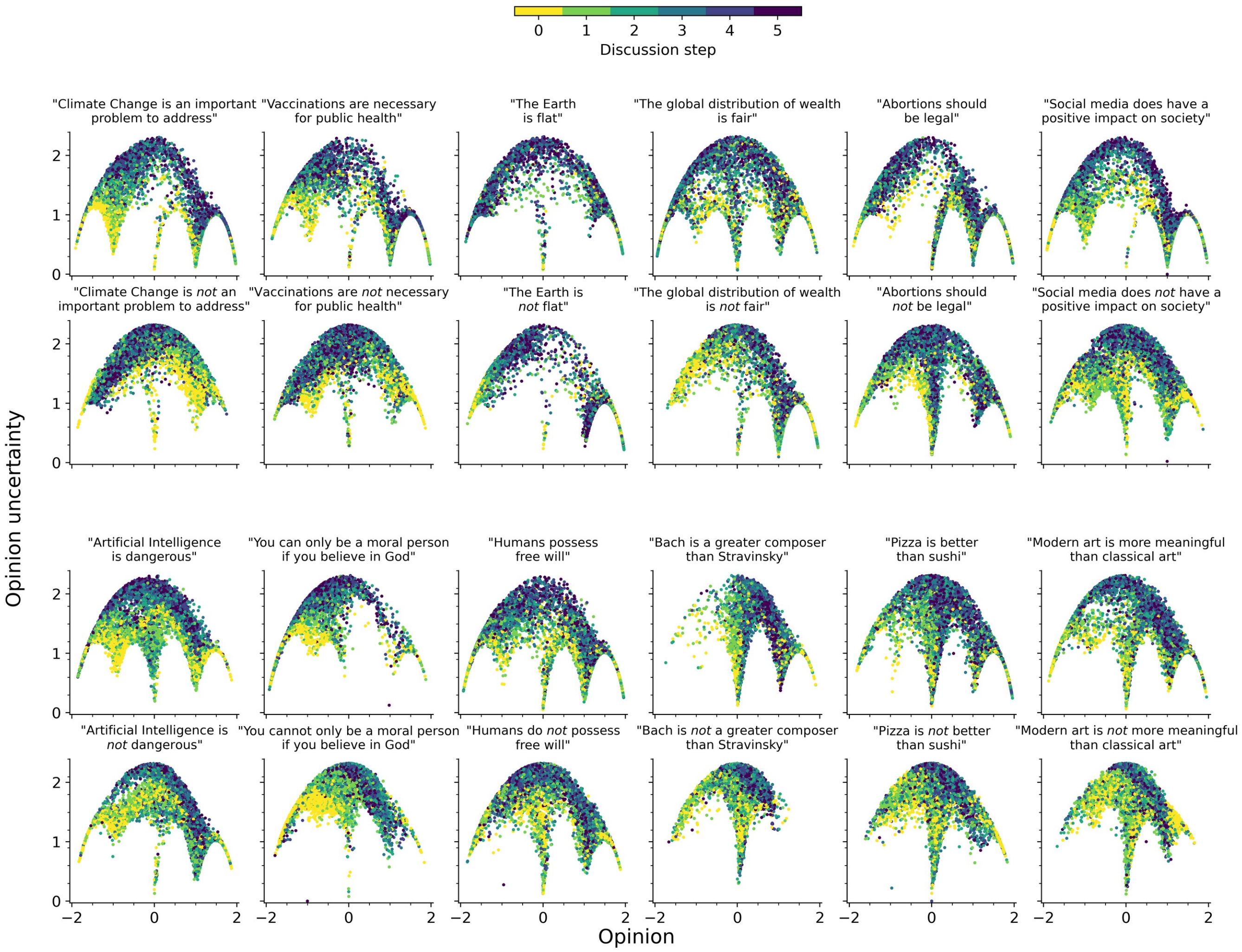

Supplementary Figure 15: Opinion entropy vs. expected opinion for the Dolphin-Mixtral LLM for the different topics and framing options.

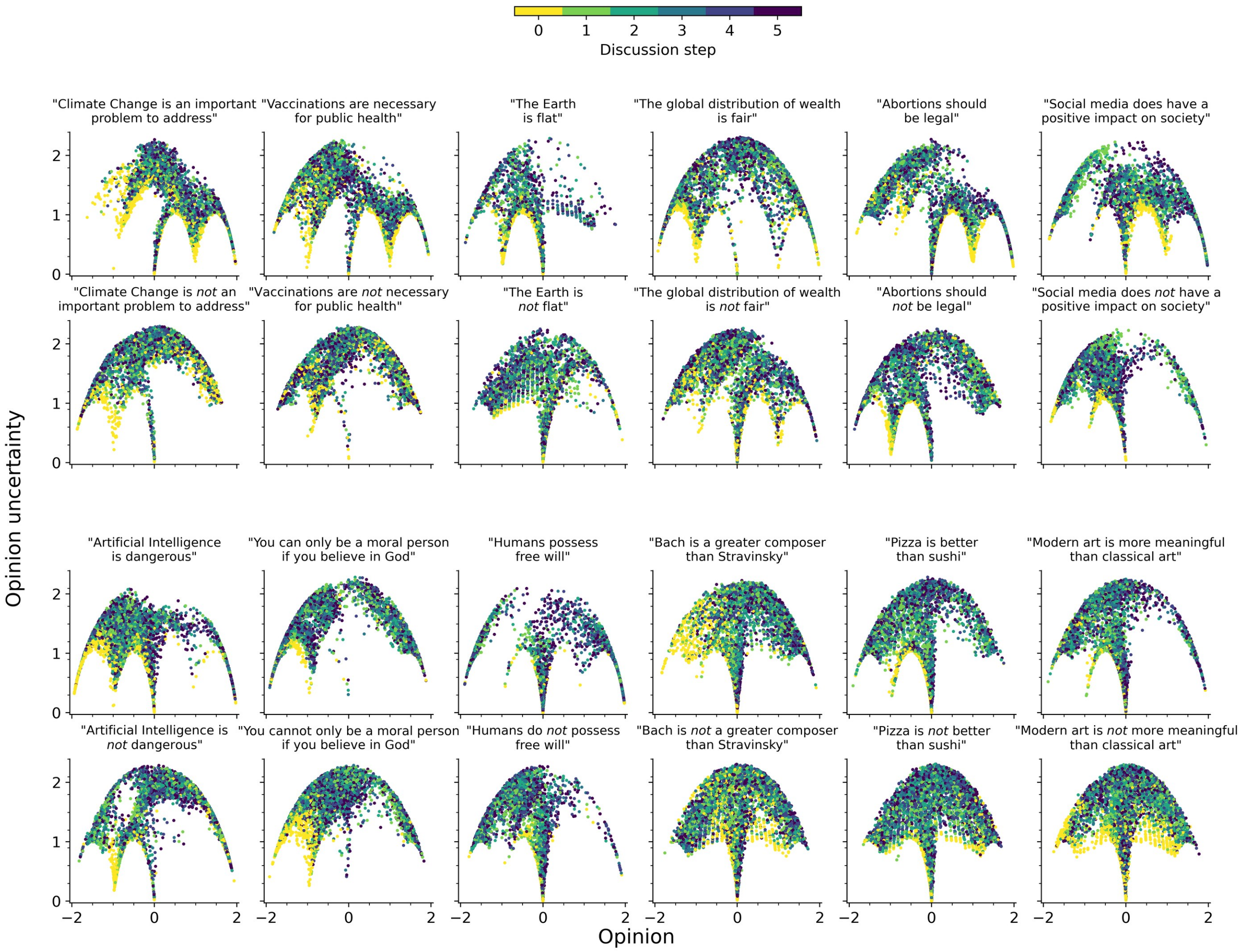

Supplementary Figure 16: Opinion entropy vs. expected opinion for the Llama-3.1-8B LLM for the different topics and framing options.

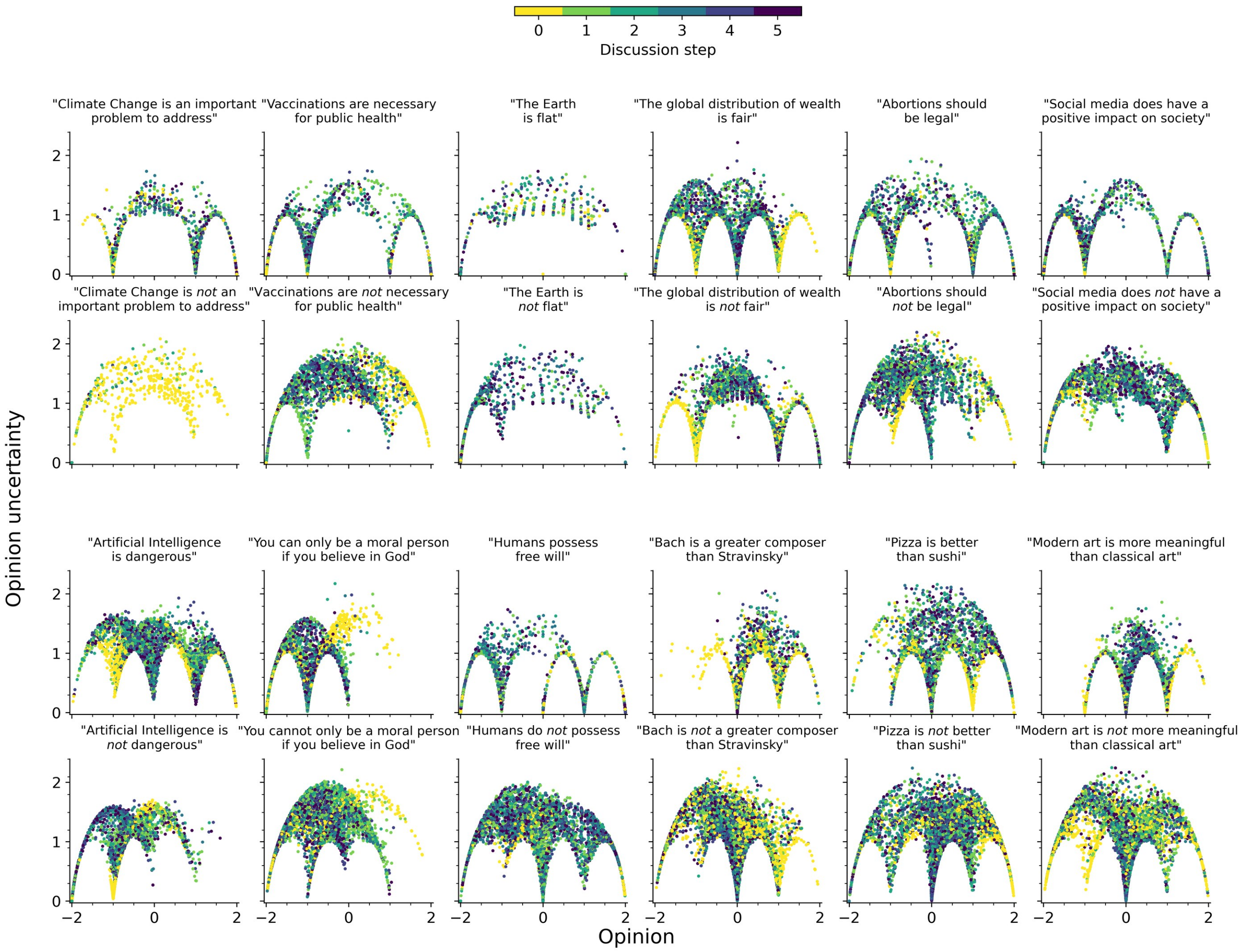

Supplementary Figure 17: Opinion entropy vs. expected opinion for the Qwen2.5-7B LLM for the different topics and framing options.

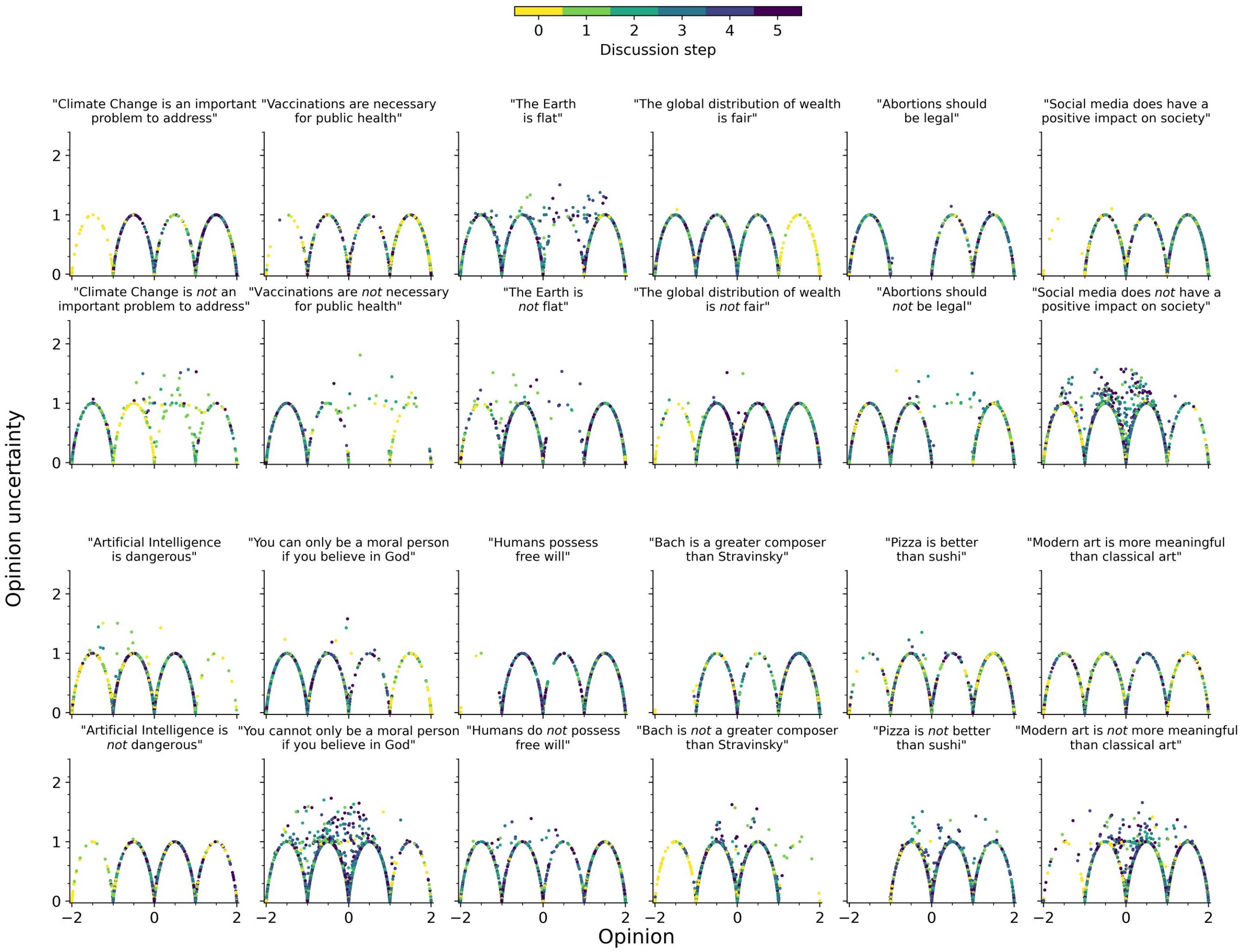

Supplementary Figure 18: Opinion entropy vs. expected opinion for the Mixtral-8x7B LLM for the different topics and framing options.

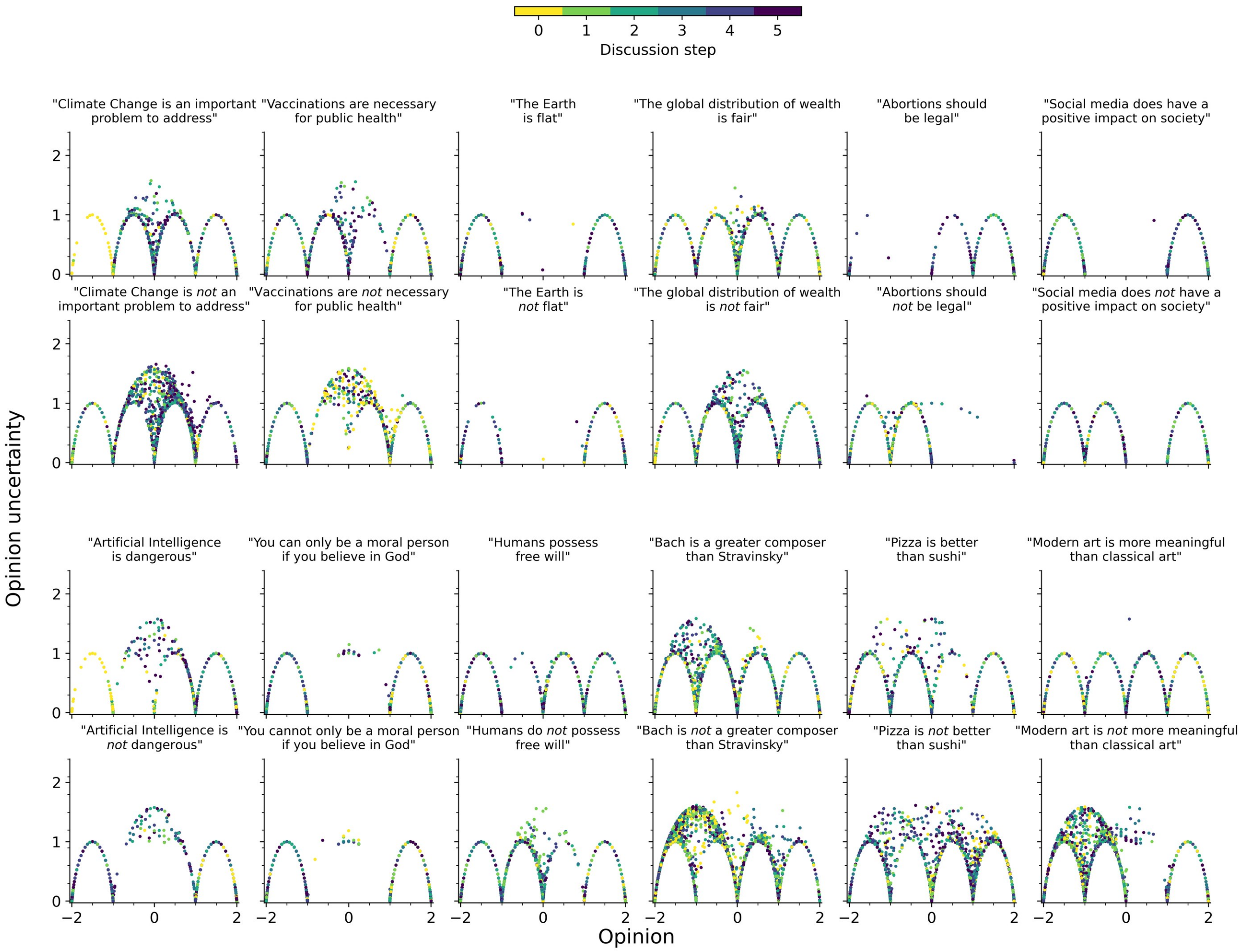

Supplementary Figure 19: Opinion entropy vs. expected opinion for the GPT-4o-mini LLM for the different topics and framing options.

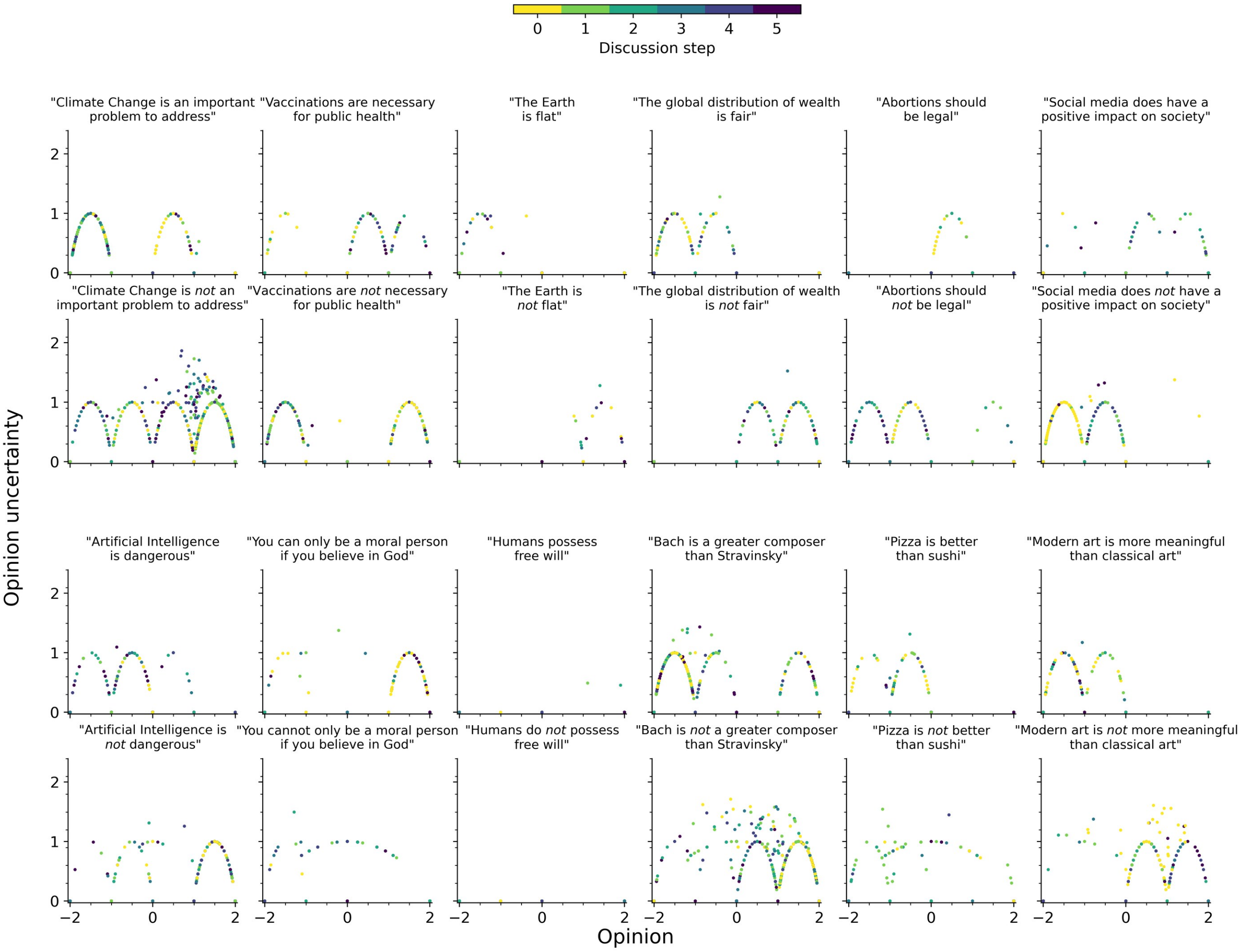

Supplementary Figure 20: Opinion entropy vs. expected opinion for the Grok-4.1-fast LLM for the different topics and framing options.

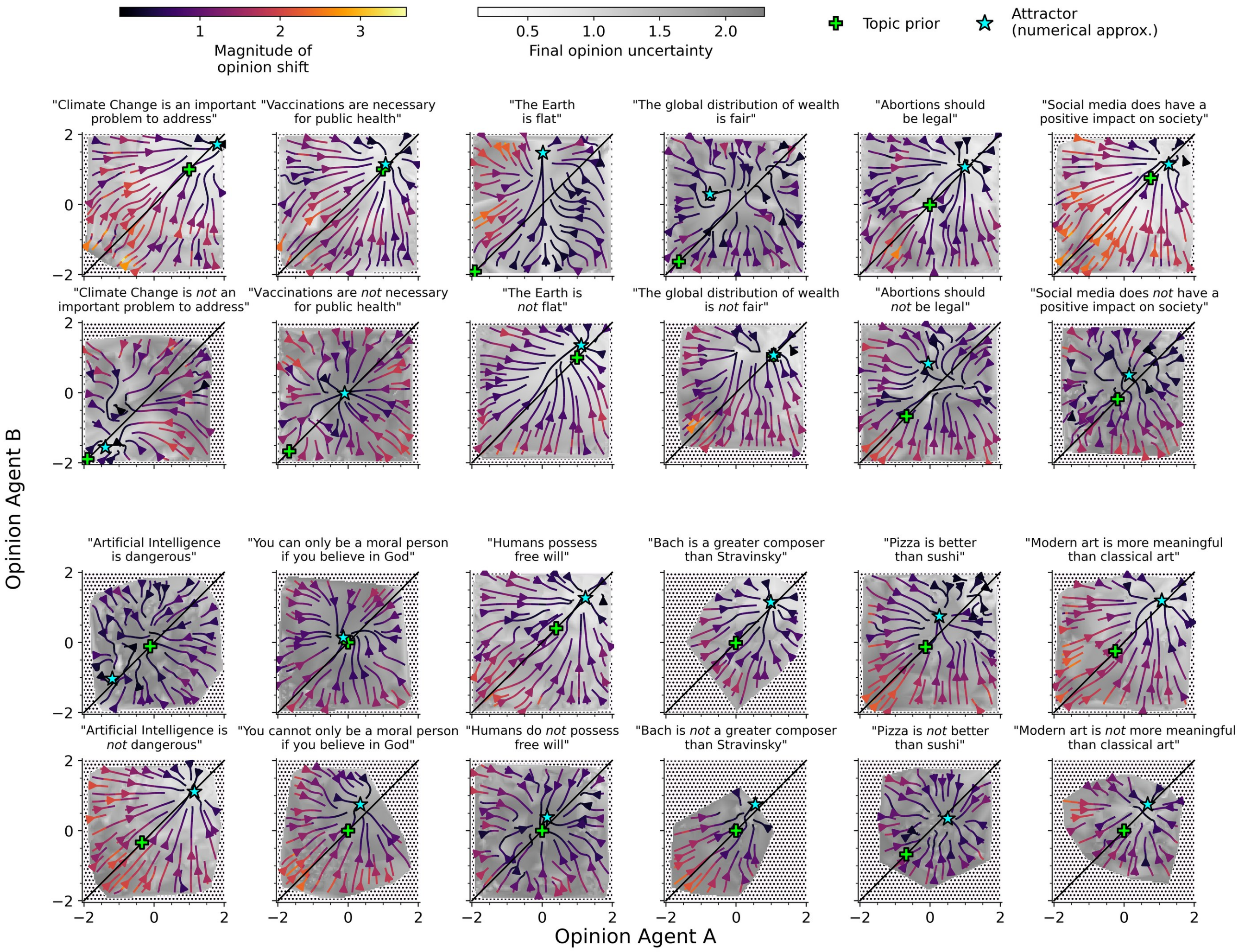

Supplementary Figure 21: Opinion stream plot for the DolphinMixtral-8x7B LLM, analogous to Fig. 3. Note that topic prior and attractor position may appear on top of each other.

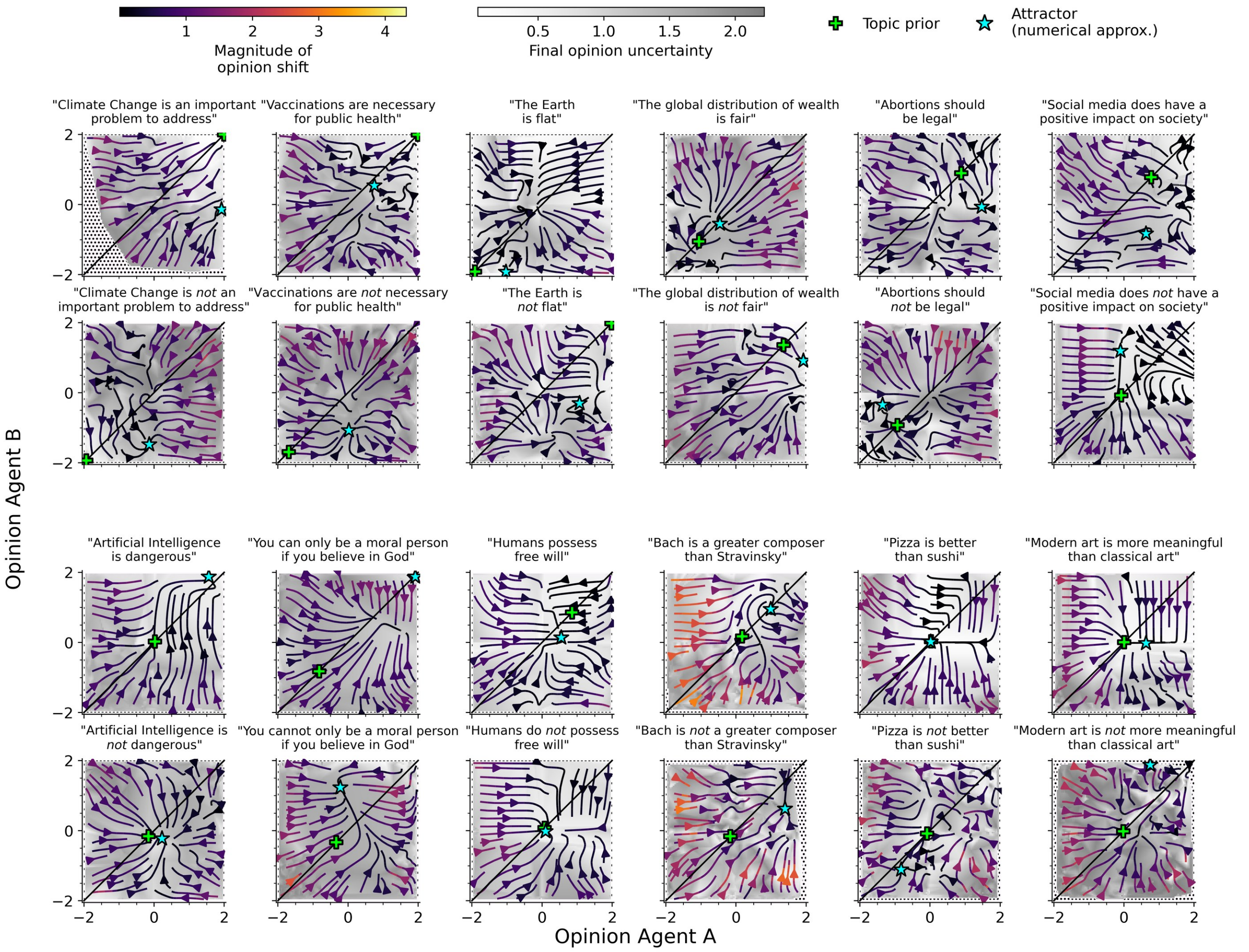

Supplementary Figure 22: Opinion stream plot for the Llama-3.1-8B LLM, analogous to Fig. 3. Note that topic prior and attractor position may appear on top of each other.

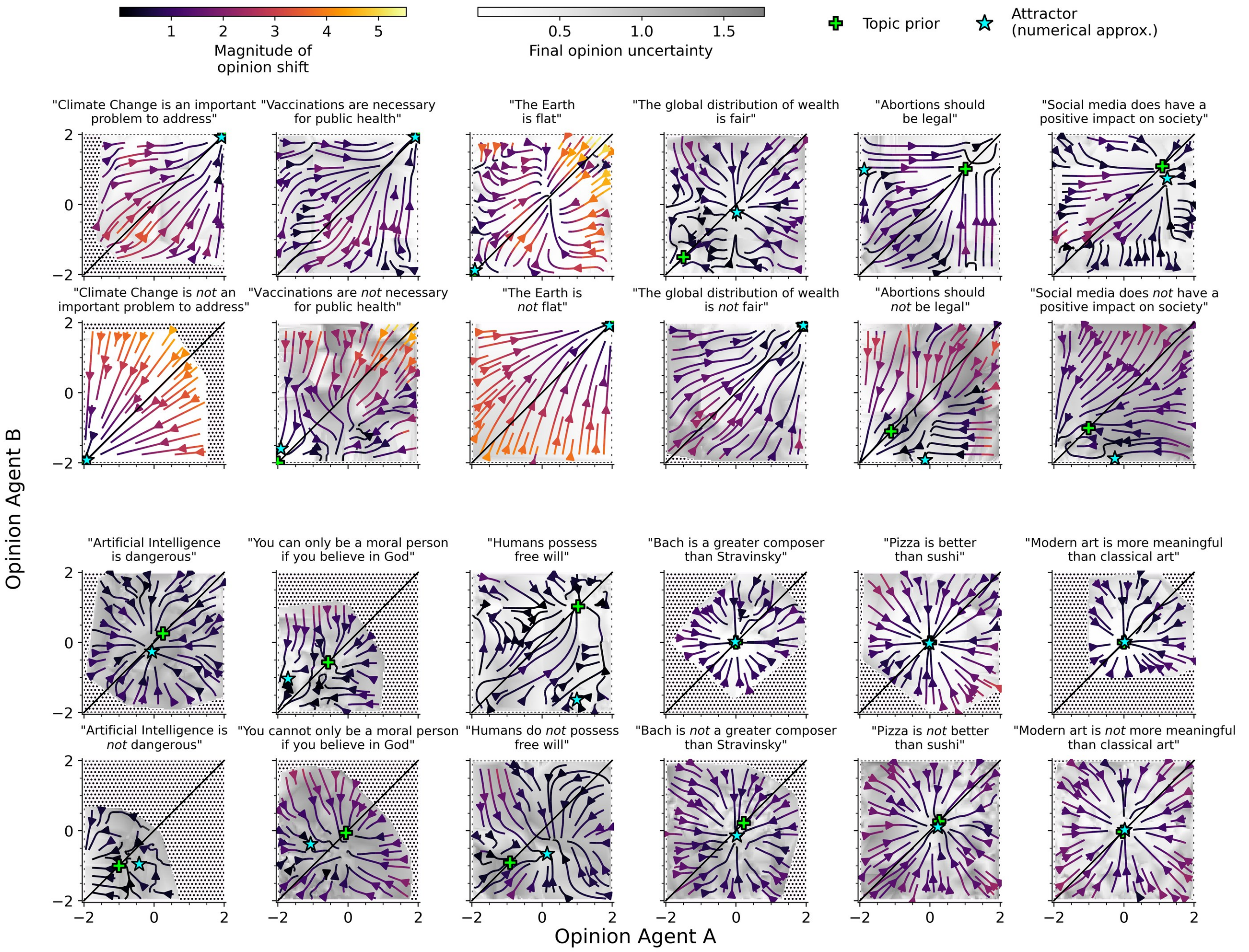

Supplementary Figure 23: Opinion stream plot for the Qwen2.5-7B LLM, analogous to Fig. 3. Note that topic prior and attractor position may appear on top of each other.

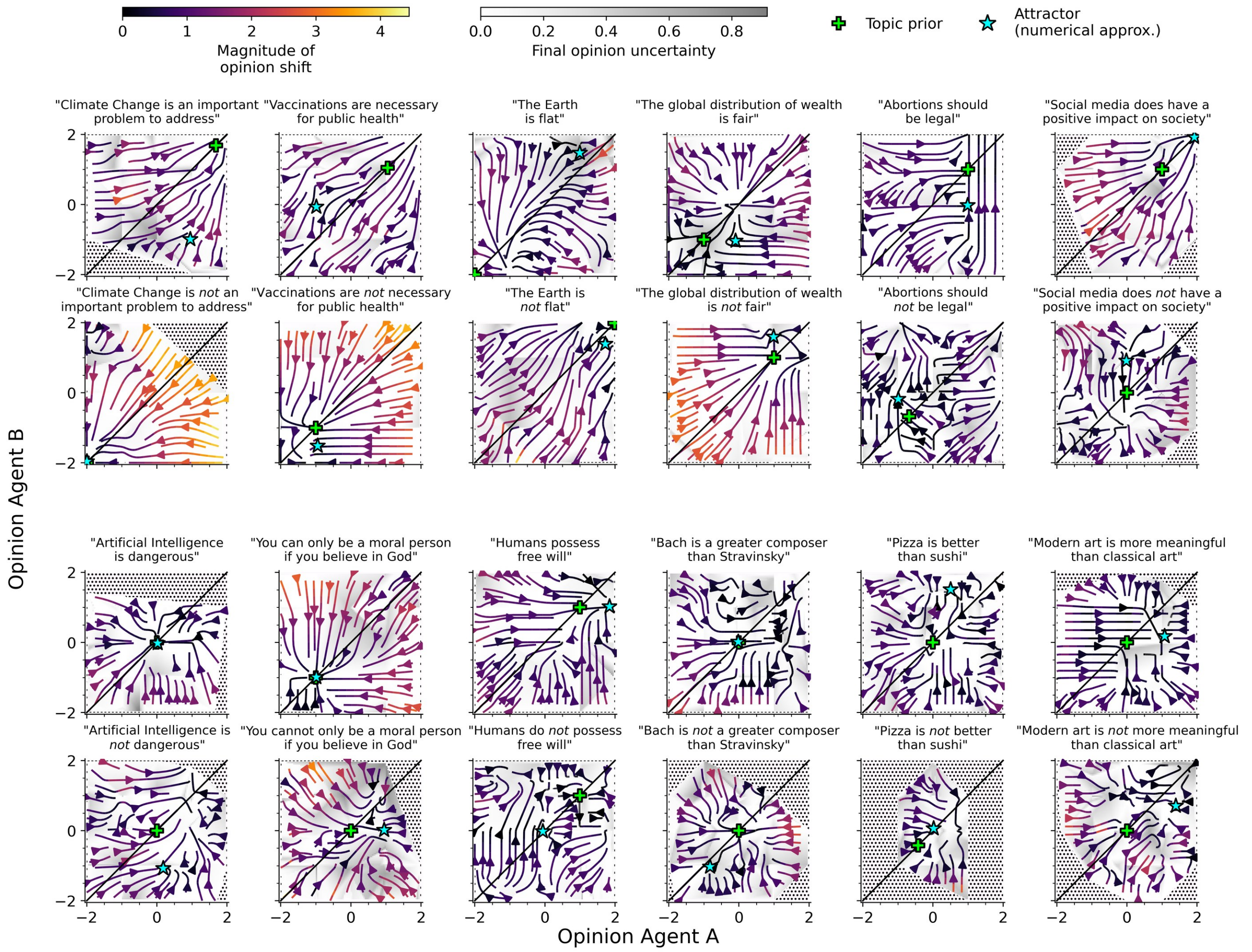

Supplementary Figure 24: Opinion stream plot for the Mixtral-8x7B LLM, analogous to Fig. 3. Note that topic prior and attractor position may appear on top of each other.

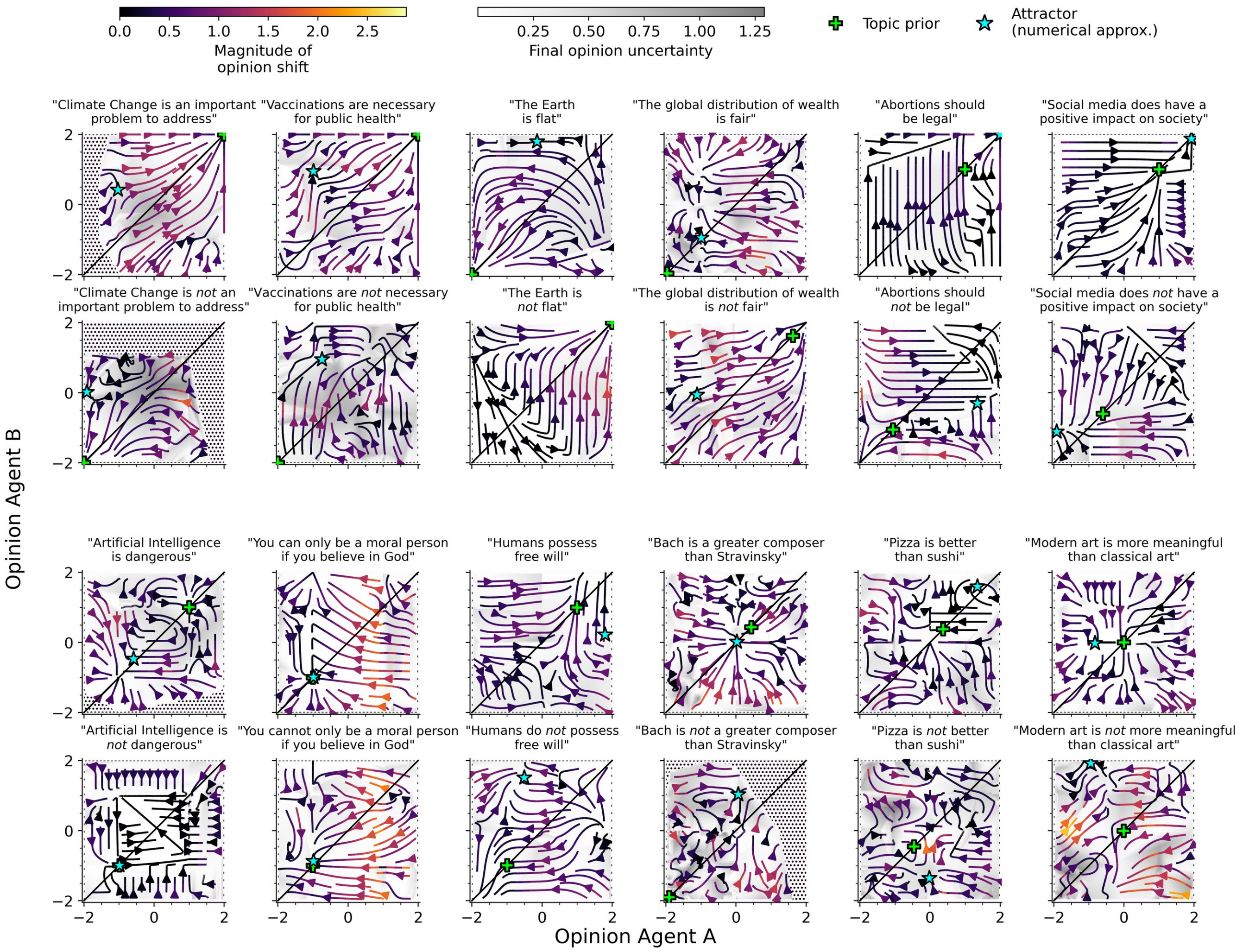

Supplementary Figure 25: Opinion stream plot for the GPT-4o-mini LLM, analogous to Fig. 3. Note that topic prior and attractor position may appear on top of each other.

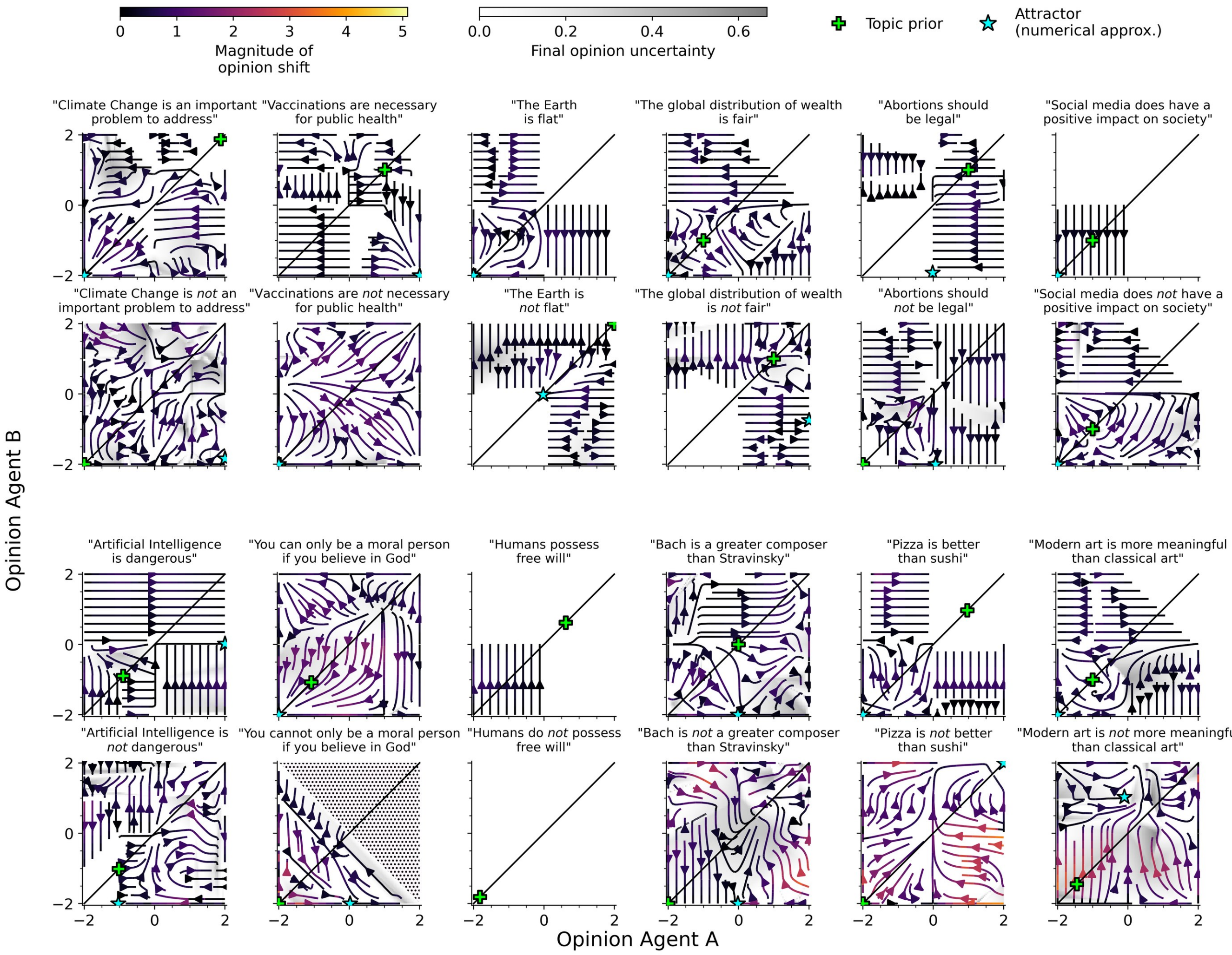

Supplementary Figure 26: Opinion stream plot for the Grok-4.1-fast LLM, analogous to Fig. 3. Note that topic prior and attractor position may appear on top of each other.

Supplementary Table 5: **Detailed posterior summaries for all global model parameters, uncertainty parameters, and topic attractors.** Posterior entries are medians with 95% HDIs.

Parameter	Description	Model	Posterior (median + HDI)	ESS_{bulk}	ESS_{tail}	$\hat{R}$
$\bar{\alpha}_{int}$	Interaction strength	Llama-3.1-8B	$0.023^{+0.006}_{-0.006}$	4854	4594	< 1.01
		Qwen2.5-7B	$0.037^{+0.014}_{-0.014}$	4841	5078	< 1.01
		DolphinMixtral-8x7B	$0.014^{+0.004}_{-0.004}$	6500	4818	< 1.01
		Mixtral-8x7B	$0.025^{+0.011}_{-0.01}$	4533	4835	< 1.01
		GPT-4o-mini	$0.013^{+0.006}_{-0.007}$	3612	3763	< 1.01
		Grok-4.1-fast	$0.004^{+0.004}_{-0.004}$	3434	3872	< 1.01
$\bar{\beta}_{top}$	Topic bias strength	Llama-3.1-8B	$0.029^{+0.013}_{-0.011}$	3502	3605	< 1.01
		Qwen2.5-7B	$0.063^{+0.028}_{-0.024}$	2860	3573	< 1.01
		DolphinMixtral-8x7B	$0.036^{+0.013}_{-0.01}$	3058	3730	< 1.01
		Mixtral-8x7B	$0.04^{+0.02}_{-0.017}$	3101	3580	< 1.01
		GPT-4o-mini	$0.006^{+0.007}_{-0.004}$	3744	3444	< 1.01
		Grok-4.1-fast	$0.002^{+0.005}_{-0.002}$	5776	4485	< 1.01
$\bar{\beta}_{agr}$	Agreement bias strength	Llama-3.1-8B	$0.022^{+0.011}_{-0.011}$	2716	3586	< 1.01
		Qwen2.5-7B	$-0.015^{+0.011}_{-0.013}$	2778	3607	< 1.01
		DolphinMixtral-8x7B	$0.01^{+0.004}_{-0.003}$	3995	3901	< 1.01
		Mixtral-8x7B	$0.019^{+0.012}_{-0.011}$	3524	4012	< 1.01
		GPT-4o-mini	$4.990e-04^{+0.003}_{-0.003}$	5398	4734	< 1.01
		Grok-4.1-fast	$-8.177e-04^{+0.007}_{-0.006}$	2991	3496	< 1.01
$\bar{\beta}_{anc}$	Anchoring bias strength	Llama-3.1-8B	$-0.036^{+0.007}_{-0.007}$	6342	4443	< 1.01
		Qwen2.5-7B	$-0.035^{+0.016}_{-0.015}$	5571	4944	< 1.01
		DolphinMixtral-8x7B	$-0.002^{+0.005}_{-0.005}$	7862	5023	< 1.01
		Mixtral-8x7B	$-0.015^{+0.007}_{-0.007}$	8950	5400	< 1.01
		GPT-4o-mini	$-0.013^{+0.007}_{-0.007}$	4340	4011	< 1.01
		Grok-4.1-fast	$-0.007^{+0.008}_{-0.008}$	3381	4187	< 1.01
τ_{int}	Interaction decay timescale	Llama-3.1-8B	$0.47^{+0.047}_{-0.048}$	7760	6284	< 1.01
		Qwen2.5-7B	$0.132^{+0.058}_{-0.073}$	6375	4152	< 1.01
		DolphinMixtral-8x7B	$2.793^{+0.535}_{-0.455}$	8528	6549	< 1.01
		Mixtral-8x7B	$1.248^{+0.19}_{-0.174}$	9121	6323	< 1.01
		GPT-4o-mini	$0.613^{+0.049}_{-0.051}$	7899	5935	< 1.01
		Grok-4.1-fast	$3.056^{+0.87}_{-0.728}$	7660	5369	< 1.01
τ_{top}	Topic-bias decay timescale	Llama-3.1-8B	$0.547^{+0.026}_{-0.027}$	8582	6758	< 1.01
		Qwen2.5-7B	$0.951^{+0.033}_{-0.03}$	8713	6271	< 1.01
		DolphinMixtral-8x7B	$9.512^{+1.369}_{-1.309}$	8628	6940	< 1.01
		Mixtral-8x7B	$2.374^{+0.156}_{-0.148}$	8881	7046	< 1.01
		GPT-4o-mini	$1.771^{+0.157}_{-0.146}$	8430	7147	< 1.01
		Grok-4.1-fast	$0.382^{+0.079}_{-0.084}$	7947	5989	< 1.01
τ_{agr}	Agreement-bias decay timescale	Llama-3.1-8B	$0.353^{+0.032}_{-0.034}$	8699	5957	< 1.01
		Qwen2.5-7B	$0.54^{+0.04}_{-0.043}$	8431	7007	< 1.01
		DolphinMixtral-8x7B	$426.559^{+488.525}_{-276.393}$	9396	4552	< 1.01
		Mixtral-8x7B	$0.511^{+0.051}_{-0.048}$	8451	6230	< 1.01
		GPT-4o-mini	$0.71^{+0.1}_{-0.093}$	8023	6700	< 1.01
		Grok-4.1-fast	$0.206^{+0.117}_{-0.113}$	7903	6654	< 1.01
τ_{anc}	Anchoring-bias decay timescale	Llama-3.1-8B	$0.236^{+0.058}_{-0.082}$	6012	4625	< 1.01
		Qwen2.5-7B	$0.37^{+0.035}_{-0.037}$	8630	6236	< 1.01
		DolphinMixtral-8x7B	$0.266^{+0.227}_{-0.176}$	8208	6874	< 1.01

Continued on next page

Parameter	Description	Model	Posterior (median + HDI)	ESS_{bulk}	ESS_{tail}	$\hat{R}$
		Mixtral-8x7B	$0.929^{+0.151}_{-0.163}$	8584	7138	< 1.01
		GPT-4o-mini	$0.564^{+0.05}_{-0.055}$	7911	6149	< 1.01
		Grok-4.1-fast	$0.593^{+0.113}_{-0.115}$	8457	6754	< 1.01
$\bar{\sigma}_0$	Baseline SD	Llama-3.1-8B	$0.189^{+0.04}_{-0.038}$	3591	4347	< 1.01
		Qwen2.5-7B	$0.196^{+0.081}_{-0.064}$	2439	3180	< 1.01
		DolphinMixtral-8x7B	$0.312^{+0.049}_{-0.041}$	2362	3208	< 1.01
		Mixtral-8x7B	$0.353^{+0.049}_{-0.049}$	2625	3303	< 1.01
		GPT-4o-mini	$0.158^{+0.034}_{-0.032}$	2672	3327	< 1.01
		Grok-4.1-fast	$0.171^{+0.095}_{-0.065}$	2028	3079	< 1.01
ϵ	Entropy contribution to SD	Llama-3.1-8B	$0.038^{+8.899e-04}_{-8.239e-04}$	8429	5440	< 1.01
		Qwen2.5-7B	$0.146^{+0.002}_{-0.002}$	8448	6282	< 1.01
		DolphinMixtral-8x7B	$0.017^{+0.001}_{-0.001}$	8494	4106	< 1.01
		Mixtral-8x7B	$0.286^{+0.011}_{-0.01}$	8916	5358	< 1.01
		GPT-4o-mini	$0.56^{+0.01}_{-0.011}$	9080	5496	< 1.01
		Grok-4.1-fast	$0.381^{+0.014}_{-0.013}$	8382	4929	< 1.01
b_1	Topic Attractor Climate Change	Llama-3.1-8B	$1.838^{+0.039}_{-0.038}$	8554	5212	< 1.01
		Qwen2.5-7B	$1.85^{+0.029}_{-0.031}$	8819	5025	< 1.01
		DolphinMixtral-8x7B	$1.26^{+0.086}_{-0.082}$	8680	6814	< 1.01
		Mixtral-8x7B	$1.878^{+0.074}_{-0.064}$	7333	4585	< 1.01
		GPT-4o-mini	$1.991^{+0.009}_{-0.028}$	6266	3898	< 1.01
		Grok-4.1-fast	$0.625^{+1.375}_{-2.264}$	8007	5548	< 1.01
b_2	Topic Attractor Vaccination	Llama-3.1-8B	$1.786^{+0.081}_{-0.073}$	7368	4819	< 1.01
		Qwen2.5-7B	$1.264^{+0.045}_{-0.04}$	8419	5154	< 1.01
		DolphinMixtral-8x7B	$0.864^{+0.061}_{-0.064}$	9153	5844	< 1.01
		Mixtral-8x7B	$1.992^{+0.008}_{-0.022}$	6760	5576	< 1.01
		GPT-4o-mini	$1.965^{+0.035}_{-0.11}$	7257	4696	< 1.01
		Grok-4.1-fast	$1.97^{+0.03}_{-0.095}$	6054	3692	< 1.01
b_3	Topic Attractor Earth Shape	Llama-3.1-8B	$-1.986^{+0.045}_{-0.014}$	7285	5296	< 1.01
		Qwen2.5-7B	$-1.046^{+0.058}_{-0.062}$	8702	5544	< 1.01
		DolphinMixtral-8x7B	$-0.694^{+0.107}_{-0.113}$	8616	6356	< 1.01
		Mixtral-8x7B	$-1.961^{+0.088}_{-0.039}$	7179	5724	< 1.01
		GPT-4o-mini	$-0.989^{+0.586}_{-0.614}$	6675	4270	< 1.01
		Grok-4.1-fast	$-1.244^{+0.668}_{-0.755}$	6368	5507	< 1.01
b_4	Topic Attractor Global Wealth	Llama-3.1-8B	$-1.994^{+0.019}_{-0.006}$	7168	5100	< 1.01
		Qwen2.5-7B	$-1.998^{+0.005}_{-0.002}$	6515	3919	< 1.01
		DolphinMixtral-8x7B	$-1.034^{+0.074}_{-0.068}$	9513	6234	< 1.01
		Mixtral-8x7B	$-1.81^{+0.11}_{-0.12}$	6818	4588	< 1.01
		GPT-4o-mini	$-1.996^{+0.012}_{-0.004}$	6548	3939	< 1.01
		Grok-4.1-fast	$1.165^{+0.835}_{-2.201}$	7301	5131	< 1.01
b_5	Topic Attractor Abortion	Llama-3.1-8B	$1.657^{+0.159}_{-0.14}$	7600	4198	< 1.01
		Qwen2.5-7B	$1.19^{+0.078}_{-0.082}$	8435	4806	< 1.01
		DolphinMixtral-8x7B	$-0.27^{+0.103}_{-0.11}$	9290	6013	< 1.01
		Mixtral-8x7B	$1.931^{+0.069}_{-0.192}$	5920	4049	< 1.01
		GPT-4o-mini	$0.771^{+1.228}_{-2.08}$	8214	4678	< 1.01
		Grok-4.1-fast	$-1.521^{+1.08}_{-0.479}$	6935	4636	< 1.01
b_6	Topic Attractor Social Media	Llama-3.1-8B	$-1.932^{+0.181}_{-0.068}$	7267	5751	< 1.01
		Qwen2.5-7B	$1.42^{+0.093}_{-0.078}$	8258	4735	< 1.01
		DolphinMixtral-8x7B	$0.831^{+0.084}_{-0.081}$	8470	6375	< 1.01

Continued on next page

Parameter	Description	Model	Posterior (median + HDI)	ESS_{bulk}	ESS_{tail}	$\hat{R}$
		Mixtral-8x7B	$1.338^{+0.208}_{-0.176}$	7609	5239	< 1.01
		GPT-4o-mini	$1.891^{+0.109}_{-0.455}$	7792	5391	< 1.01
		Grok-4.1-fast	$-1.794^{+0.483}_{-0.206}$	7015	5209	< 1.01
b_7	Topic Attractor AI	Llama-3.1-8B	$1.599^{+0.087}_{-0.084}$	8378	5466	< 1.01
		Qwen2.5-7B	$-1.036^{+0.435}_{-0.541}$	6336	3738	< 1.01
		DolphinMixtral-8x7B	$-1.028^{+0.06}_{-0.062}$	8649	6021	< 1.01
		Mixtral-8x7B	$0.233^{+0.066}_{-0.064}$	8549	5930	< 1.01
		GPT-4o-mini	$0.966^{+0.167}_{-0.147}$	8560	6058	< 1.01
		Grok-4.1-fast	$0.059^{+1.728}_{-2.058}$	8435	4268	< 1.01
b_8	Topic Attractor Morality	Llama-3.1-8B	$-1.203^{+0.088}_{-0.099}$	8454	5397	< 1.01
		Qwen2.5-7B	$-1.574^{+0.194}_{-0.214}$	7463	4279	< 1.01
		DolphinMixtral-8x7B	$-0.575^{+0.03}_{-0.029}$	8938	6315	< 1.01
		Mixtral-8x7B	$-0.728^{+0.075}_{-0.083}$	9105	5390	< 1.01
		GPT-4o-mini	$0.055^{+0.121}_{-0.119}$	9108	5658	< 1.01
		Grok-4.1-fast	$-1.982^{+0.058}_{-0.018}$	5612	3427	< 1.01
b_9	Topic Attractor Free Will	Llama-3.1-8B	$-1.992^{+0.026}_{-0.008}$	6519	4504	< 1.01
		Qwen2.5-7B	$-0.073^{+0.105}_{-0.111}$	8918	5144	< 1.01
		DolphinMixtral-8x7B	$0.357^{+0.074}_{-0.074}$	9649	5096	< 1.01
		Mixtral-8x7B	$1.213^{+0.248}_{-0.225}$	7836	4693	< 1.01
		GPT-4o-mini	$-1.694^{+0.541}_{-0.306}$	7012	5643	< 1.01
		Grok-4.1-fast	$-0.741^{+1.75}_{-1.258}$	7445	4357	< 1.01
b_{10}	Topic Attractor Music Preference	Llama-3.1-8B	$1.173^{+0.081}_{-0.077}$	8758	5939	< 1.01
		Qwen2.5-7B	$0.135^{+0.016}_{-0.015}$	8424	5129	< 1.01
		DolphinMixtral-8x7B	$0.167^{+0.024}_{-0.025}$	9541	5313	< 1.01
		Mixtral-8x7B	$0.261^{+0.05}_{-0.05}$	8427	4835	< 1.01
		GPT-4o-mini	$0.246^{+0.067}_{-0.074}$	8658	5584	< 1.01
		Grok-4.1-fast	$0.772^{+0.152}_{-0.126}$	8421	6870	< 1.01
b_{11}	Topic Attractor Food Preference	Llama-3.1-8B	$-0.301^{+0.098}_{-0.1}$	9114	5751	< 1.01
		Qwen2.5-7B	$0.101^{+0.031}_{-0.032}$	8888	5573	< 1.01
		DolphinMixtral-8x7B	$0.209^{+0.048}_{-0.046}$	8953	4978	< 1.01
		Mixtral-8x7B	$-0.049^{+0.04}_{-0.043}$	8714	5182	< 1.01
		GPT-4o-mini	$-0.641^{+0.343}_{-0.353}$	8236	6289	< 1.01
		Grok-4.1-fast	$0.952^{+0.16}_{-0.148}$	8119	6491	< 1.01
b_{12}	Topic Attractor Art Style	Llama-3.1-8B	$-0.358^{+0.093}_{-0.103}$	8639	6567	< 1.01
		Qwen2.5-7B	$-0.016^{+0.029}_{-0.029}$	9089	5512	< 1.01
		DolphinMixtral-8x7B	$0.214^{+0.052}_{-0.051}$	9597	4373	< 1.01
		Mixtral-8x7B	$-0.347^{+0.066}_{-0.062}$	9249	4719	< 1.01
		GPT-4o-mini	$0.179^{+0.097}_{-0.099}$	8848	5158	< 1.01
		Grok-4.1-fast	$1.985^{+0.015}_{-0.047}$	6621	4467	< 1.01

Supplementary Table 6: **Detailed posterior summaries for topic-specific interaction strength parameter $\alpha_{\text{interact},k}$.** Posterior entries are medians with 95% HDIs.

Parameter	Description	Model	Posterior (median + HDI)	ESS_{bulk}	ESS_{tail}	$\hat{R}$
		Llama-3.1-8B	$0.012^{+0.001}_{-0.001}$	8197	7512	< 1.01
		Qwen2.5-7B	$0.018^{+0.002}_{-0.002}$	8083	7125	< 1.01

Continued on next page

Parameter	Description	Model	Posterior (median + HDI)	ESS_{bulk}	ESS_{tail}	$\hat{R}$
$\alpha_{int,1}$	Interaction strength Climate Change	DolphinMixtral-8x7B	$0.009^{+0.003}_{-0.003}$	8300	6573	< 1.01
		Mixtral-8x7B	$0.012^{+0.004}_{-0.004}$	8598	7262	< 1.01
		GPT-4o-mini	$-6.460e-04^{+8.736e-04}_{-9.017e-04}$	8145	6253	< 1.01
		Grok-4.1-fast	$0.006^{+0.001}_{-0.001}$	7799	6859	< 1.01
$\alpha_{int,2}$	Interaction strength Global Wealth	Llama-3.1-8B	$0.02^{+0.001}_{-0.001}$	8403	6218	< 1.01
		Qwen2.5-7B	$0.016^{+0.001}_{-0.001}$	8154	6225	< 1.01
		DolphinMixtral-8x7B	$0.015^{+0.002}_{-0.002}$	8780	7125	< 1.01
		Mixtral-8x7B	$0.044^{+0.003}_{-0.003}$	8637	7468	< 1.01
		GPT-4o-mini	$0.012^{+0.001}_{-0.001}$	8696	6896	< 1.01
		Grok-4.1-fast	$2.484e-04^{+4.318e-04}_{-4.007e-04}$	7918	6855	< 1.01
$\alpha_{int,3}$	Interaction strength AI	Llama-3.1-8B	$0.028^{+0.001}_{-0.001}$	8410	7196	< 1.01
		Qwen2.5-7B	$0.01^{+0.001}_{-0.001}$	7991	7616	< 1.01
		DolphinMixtral-8x7B	$0.013^{+0.003}_{-0.002}$	8893	6985	< 1.01
		Mixtral-8x7B	$0.011^{+0.003}_{-0.003}$	8734	6996	< 1.01
		GPT-4o-mini	$0.017^{+0.001}_{-0.001}$	8959	6837	< 1.01
		Grok-4.1-fast	$0.004^{+7.425e-04}_{-8.107e-04}$	7592	7247	< 1.01
$\alpha_{int,4}$	Interaction strength Music Preference	Llama-3.1-8B	$0.02^{+0.003}_{-0.003}$	9114	6727	< 1.01
		Qwen2.5-7B	$0.029^{+0.003}_{-0.003}$	8058	6552	< 1.01
		DolphinMixtral-8x7B	$0.009^{+0.004}_{-0.004}$	8206	6375	< 1.01
		Mixtral-8x7B	$0.018^{+0.004}_{-0.004}$	8432	6884	< 1.01
		GPT-4o-mini	$0.009^{+0.003}_{-0.004}$	8720	5952	< 1.01
		Grok-4.1-fast	$0.009^{+0.002}_{-0.002}$	7834	6828	< 1.01
$\alpha_{int,5}$	Interaction strength Vaccination	Llama-3.1-8B	$0.018^{+0.001}_{-0.001}$	7875	7036	< 1.01
		Qwen2.5-7B	$0.041^{+0.002}_{-0.002}$	8184	6737	< 1.01
		DolphinMixtral-8x7B	$0.011^{+0.003}_{-0.003}$	9286	6159	< 1.01
		Mixtral-8x7B	$0.02^{+0.003}_{-0.004}$	8830	6640	< 1.01
		GPT-4o-mini	$0.007^{+8.695e-04}_{-9.788e-04}$	8955	7036	< 1.01
		Grok-4.1-fast	$-2.619e-04^{+5.352e-04}_{-5.358e-04}$	8018	6953	< 1.01
$\alpha_{int,6}$	Interaction strength Abortion	Llama-3.1-8B	$0.024^{+0.001}_{-0.001}$	8161	6893	< 1.01
		Qwen2.5-7B	$0.07^{+0.003}_{-0.003}$	8105	7687	< 1.01
		DolphinMixtral-8x7B	$0.013^{+0.002}_{-0.002}$	8824	7326	< 1.01
		Mixtral-8x7B	$0.02^{+0.002}_{-0.002}$	8798	7522	< 1.01
		GPT-4o-mini	$0.001^{+9.686e-04}_{-0.001}$	7780	6754	< 1.01
		Grok-4.1-fast	$1.809e-04^{+3.718e-04}_{-3.294e-04}$	8078	7295	< 1.01
$\alpha_{int,7}$	Interaction strength Morality	Llama-3.1-8B	$0.027^{+0.002}_{-0.002}$	7710	6827	< 1.01
		Qwen2.5-7B	$0.028^{+0.004}_{-0.004}$	8716	6386	< 1.01
		DolphinMixtral-8x7B	$0.029^{+0.005}_{-0.006}$	6747	6278	< 1.01
		Mixtral-8x7B	$0.066^{+0.006}_{-0.006}$	7861	7184	< 1.01
		GPT-4o-mini	$0.019^{+0.002}_{-0.002}$	9112	7284	< 1.01
		Grok-4.1-fast	$0.012^{+0.003}_{-0.003}$	8244	6186	< 1.01
$\alpha_{int,8}$	Interaction strength Food Preference	Llama-3.1-8B	$0.042^{+0.003}_{-0.002}$	8564	7030	< 1.01
		Qwen2.5-7B	$0.031^{+0.003}_{-0.003}$	8445	5367	< 1.01
		DolphinMixtral-8x7B	$0.019^{+0.003}_{-0.003}$	9166	6177	< 1.01
		Mixtral-8x7B	$0.025^{+0.005}_{-0.005}$	9133	6193	< 1.01
		GPT-4o-mini	$0.025^{+0.002}_{-0.002}$	8450	7303	< 1.01

Continued on next page

Parameter	Description	Model	Posterior (median + HDI)	ESS_{bulk}	ESS_{tail}	$\hat{R}$
		Grok-4.1-fast	$0.015^{+0.003}_{-0.003}$	8038	6596	< 1.01
$\alpha_{int,9}$	Interaction strength Earth Shape	Llama-3.1-8B	$0.01^{+0.001}_{-0.001}$	7830	6076	< 1.01
		Qwen2.5-7B	$0.051^{+0.004}_{-0.004}$	8643	5532	< 1.01
		DolphinMixtral-8x7B	$0.013^{+0.002}_{-0.002}$	8745	6963	< 1.01
		Mixtral-8x7B	$0.021^{+0.003}_{-0.003}$	8860	7017	< 1.01
		GPT-4o-mini	$0.002^{+4.364e-04}_{-4.246e-04}$	8108	7392	< 1.01
		Grok-4.1-fast	$4.363e-05^{+1.671e-04}_{-1.774e-04}$	7555	6515	< 1.01
$\alpha_{int,10}$	Interaction strength Social Media	Llama-3.1-8B	$0.036^{+0.001}_{-9.396e-04}$	8384	7447	< 1.01
		Qwen2.5-7B	$0.055^{+0.002}_{-0.002}$	8047	7461	< 1.01
		DolphinMixtral-8x7B	$0.015^{+0.003}_{-0.003}$	9199	6657	< 1.01
		Mixtral-8x7B	$0.031^{+0.005}_{-0.005}$	8590	7302	< 1.01
		GPT-4o-mini	$0.025^{+8.977e-04}_{-8.877e-04}$	8109	7081	< 1.01
		Grok-4.1-fast	$3.299e-04^{+4.867e-04}_{-5.178e-04}$	7955	6657	< 1.01
$\alpha_{int,11}$	Interaction strength Free Will	Llama-3.1-8B	$0.013^{+9.858e-04}_{-9.026e-04}$	7504	7479	< 1.01
		Qwen2.5-7B	$0.078^{+0.003}_{-0.003}$	8537	7580	< 1.01
		DolphinMixtral-8x7B	$0.01^{+0.002}_{-0.002}$	9114	6915	< 1.01
		Mixtral-8x7B	$0.028^{+0.002}_{-0.003}$	9059	7892	< 1.01
		GPT-4o-mini	$0.023^{+8.580e-04}_{-9.407e-04}$	8284	7194	< 1.01
		Grok-4.1-fast	$5.402e-05^{+5.213e-05}_{-5.348e-05}$	7776	6430	< 1.01
$\alpha_{int,12}$	Interaction strength Art Style	Llama-3.1-8B	$0.021^{+0.003}_{-0.002}$	8064	6780	< 1.01
		Qwen2.5-7B	$0.029^{+0.003}_{-0.003}$	8639	7238	< 1.01
		DolphinMixtral-8x7B	$0.017^{+0.003}_{-0.003}$	8329	6736	< 1.01
		Mixtral-8x7B	$0.012^{+0.004}_{-0.005}$	8533	6518	< 1.01
		GPT-4o-mini	$0.02^{+0.002}_{-0.002}$	8766	6697	< 1.01
		Grok-4.1-fast	$-1.775e-04^{+5.985e-04}_{-6.178e-04}$	8077	6328	< 1.01

Supplementary Table 7: **Detailed posterior summaries for topic-specific topic-bias strength parameter $\beta_{topic,k}$.** Posterior entries are medians with 95% HDIs.

Parameter	Description	Model	Posterior (median + HDI)	ESS_{bulk}	ESS_{tail}	$\hat{R}$
$\beta_{top,1}$	Topic bias strength Climate Change	Llama-3.1-8B	$0.059^{+0.002}_{-0.002}$	8222	6996	< 1.01
		Qwen2.5-7B	$0.107^{+0.003}_{-0.003}$	8506	6361	< 1.01
		DolphinMixtral-8x7B	$0.035^{+0.003}_{-0.003}$	8171	6907	< 1.01
		Mixtral-8x7B	$0.05^{+0.004}_{-0.004}$	8052	6602	< 1.01
		GPT-4o-mini	$0.007^{+6.646e-04}_{-6.566e-04}$	8178	7172	< 1.01
		Grok-4.1-fast	$6.149e-05^{+3.363e-04}_{-6.149e-05}$	7486	6158	< 1.01
$\beta_{top,2}$	Topic bias strength Global Wealth	Llama-3.1-8B	$0.041^{+0.001}_{-9.738e-04}$	8132	7736	< 1.01
		Qwen2.5-7B	$0.055^{+0.001}_{-0.001}$	8125	7542	< 1.01
		DolphinMixtral-8x7B	$0.026^{+0.002}_{-0.002}$	8869	7563	< 1.01
		Mixtral-8x7B	$0.044^{+0.003}_{-0.003}$	6841	6170	< 1.01
		GPT-4o-mini	$0.019^{+0.001}_{-0.001}$	7099	7015	< 1.01
		Grok-4.1-fast	$3.908e-04^{+6.536e-04}_{-3.908e-04}$	5150	5592	< 1.01
$\beta_{top,3}$	Topic bias strength AI	Llama-3.1-8B	$0.035^{+0.002}_{-0.002}$	8593	6853	< 1.01
		Qwen2.5-7B	$0.011^{+0.002}_{-0.002}$	6798	4110	< 1.01
		DolphinMixtral-8x7B	$0.038^{+0.002}_{-0.002}$	8590	6597	< 1.01
		Mixtral-8x7B	$0.044^{+0.004}_{-0.004}$	8596	7422	< 1.01

Continued on next page

Parameter	Description	Model	Posterior (median + HDI)	ESS_{bulk}	ESS_{tail}	$\hat{R}$
		GPT-4o-mini	$0.009^{+0.001}_{-0.001}$	8664	6797	< 1.01
		Grok-4.1-fast	$6.689e-05^{+2.715e-04}_{-6.689e-05}$	7330	5419	< 1.01
$\beta_{top,4}$	Topic bias strength Music Preference	Llama-3.1-8B	$0.055^{+0.004}_{-0.004}$	8668	7240	< 1.01
		Qwen2.5-7B	$0.108^{+0.004}_{-0.004}$	8343	7417	< 1.01
		DolphinMixtral-8x7B	$0.056^{+0.004}_{-0.004}$	8492	7501	< 1.01
		Mixtral-8x7B	$0.055^{+0.004}_{-0.004}$	8110	7308	< 1.01
		GPT-4o-mini	$0.037^{+0.004}_{-0.004}$	8378	7439	< 1.01
		Grok-4.1-fast	$0.043^{+0.004}_{-0.004}$	8086	7084	< 1.01
$\beta_{top,5}$	Topic bias strength Vaccination	Llama-3.1-8B	$0.031^{+0.002}_{-0.001}$	7494	6610	< 1.01
		Qwen2.5-7B	$0.087^{+0.003}_{-0.003}$	8224	7274	< 1.01
		DolphinMixtral-8x7B	$0.043^{+0.003}_{-0.003}$	8977	6902	< 1.01
		Mixtral-8x7B	$0.054^{+0.003}_{-0.003}$	8790	7367	< 1.01
		GPT-4o-mini	$0.004^{+6.802e-04}_{-6.699e-04}$	7798	6750	< 1.01
		Grok-4.1-fast	$0.004^{+7.451e-04}_{-7.496e-04}$	7842	6373	< 1.01
$\beta_{top,6}$	Topic bias strength Abortion	Llama-3.1-8B	$0.02^{+0.002}_{-0.002}$	8350	5658	< 1.01
		Qwen2.5-7B	$0.069^{+0.004}_{-0.004}$	8021	6917	< 1.01
		DolphinMixtral-8x7B	$0.017^{+0.002}_{-0.002}$	8576	7392	< 1.01
		Mixtral-8x7B	$0.006^{+0.001}_{-0.002}$	8004	6605	< 1.01
		GPT-4o-mini	$4.171e-04^{+6.244e-04}_{-4.155e-04}$	6766	5688	< 1.01
		Grok-4.1-fast	$6.993e-04^{+5.132e-04}_{-6.992e-04}$	5807	4454	< 1.01
$\beta_{top,7}$	Topic bias strength Morality	Llama-3.1-8B	$0.04^{+0.002}_{-0.002}$	8016	6432	< 1.01
		Qwen2.5-7B	$0.047^{+0.006}_{-0.006}$	7395	4574	< 1.01
		DolphinMixtral-8x7B	$0.102^{+0.005}_{-0.005}$	8002	7457	< 1.01
		Mixtral-8x7B	$0.092^{+0.007}_{-0.007}$	8253	6719	< 1.01
		GPT-4o-mini	$0.016^{+0.002}_{-0.002}$	8371	7261	< 1.01
		Grok-4.1-fast	$0.029^{+0.003}_{-0.003}$	8036	6540	< 1.01
$\beta_{top,8}$	Topic bias strength Food Preference	Llama-3.1-8B	$0.037^{+0.003}_{-0.004}$	8332	6804	< 1.01
		Qwen2.5-7B	$0.1^{+0.003}_{-0.003}$	8299	7757	< 1.01
		DolphinMixtral-8x7B	$0.038^{+0.003}_{-0.003}$	8924	7889	< 1.01
		Mixtral-8x7B	$0.07^{+0.005}_{-0.005}$	8705	7224	< 1.01
		GPT-4o-mini	$0.008^{+0.002}_{-0.002}$	8603	7079	< 1.01
		Grok-4.1-fast	$0.037^{+0.004}_{-0.004}$	8403	7359	< 1.01
$\beta_{top,9}$	Topic bias strength Earth Shape	Llama-3.1-8B	$0.023^{+0.001}_{-9.832e-04}$	8437	7562	< 1.01
		Qwen2.5-7B	$0.114^{+0.005}_{-0.005}$	7919	6963	< 1.01
		DolphinMixtral-8x7B	$0.021^{+0.002}_{-0.002}$	8242	7372	< 1.01
		Mixtral-8x7B	$0.031^{+0.003}_{-0.003}$	7818	7491	< 1.01
		GPT-4o-mini	$0.001^{+4.512e-04}_{-4.661e-04}$	6779	5575	< 1.01
		Grok-4.1-fast	$6.185e-04^{+3.615e-04}_{-3.239e-04}$	5614	4794	< 1.01
$\beta_{top,10}$	Topic bias strength Social Media	Llama-3.1-8B	$0.005^{+8.226e-04}_{-7.547e-04}$	8282	7449	< 1.01
		Qwen2.5-7B	$0.04^{+0.002}_{-0.002}$	8392	6697	< 1.01
		DolphinMixtral-8x7B	$0.03^{+0.002}_{-0.003}$	8434	7024	< 1.01
		Mixtral-8x7B	$0.03^{+0.005}_{-0.005}$	7725	6196	< 1.01
		GPT-4o-mini	$8.024e-04^{+6.102e-04}_{-6.492e-04}$	7270	5350	< 1.01
		Grok-4.1-fast	$0.002^{+7.221e-04}_{-7.391e-04}$	7812	6429	< 1.01
		Llama-3.1-8B	$0.014^{+6.806e-04}_{-6.679e-04}$	8071	7790	< 1.01
		Qwen2.5-7B	$0.035^{+0.003}_{-0.003}$	8243	6592	< 1.01

Continued on next page

Parameter	Description	Model	Posterior (median + HDI)	$\mathrm{ESS}_{\mathrm{bulk}}$	$\mathrm{ESS}_{\mathrm{tail}}$	$\hat{R}$
$\beta_{\mathrm{top},11}$	Topic bias strength Free Will	DolphinMixtral-8x7B	$0.027^{+0.002}_{-0.002}$	8109	7398	< 1.01
		Mixtral-8x7B	$0.017^{+0.003}_{-0.003}$	7570	6110	< 1.01
		GPT-4o-mini	$0.002^{+7.560e-04}_{-6.574e-04}$	8297	5793	< 1.01
		Grok-4.1-fast	$6.851e-05^{+9.557e-05}_{-6.848e-05}$	3854	3895	< 1.01
$\beta_{\mathrm{top},12}$	Topic bias strength Art Style	Llama-3.1-8B	$0.038^{+0.003}_{-0.003}$	7849	6529	< 1.01
		Qwen2.5-7B	$0.083^{+0.003}_{-0.003}$	8406	7237	< 1.01
		DolphinMixtral-8x7B	$0.033^{+0.003}_{-0.002}$	8643	6998	< 1.01
		Mixtral-8x7B	$0.058^{+0.005}_{-0.005}$	8071	7174	< 1.01
		GPT-4o-mini	$0.021^{+0.002}_{-0.002}$	8271	7687	< 1.01
		Grok-4.1-fast	$0.008^{+7.639e-04}_{-7.449e-04}$	8280	7135	< 1.01

Supplementary Table 8: **Detailed posterior summaries for topic-specific agreement-bias strength parameter $\beta_{\mathrm{agree},k}$.** Posterior entries are medians with 95% HDIs.

Parameter	Description	Model	Posterior (median + HDI)	$\mathrm{ESS}_{\mathrm{bulk}}$	$\mathrm{ESS}_{\mathrm{tail}}$	$\hat{R}$
$\beta_{\mathrm{agr},1}$	Agreement bias strength Climate Change	Llama-3.1-8B	$0.006^{+7.491e-04}_{-6.849e-04}$	7989	7127	< 1.01
		Qwen2.5-7B	$-0.009^{+0.001}_{-0.001}$	8117	6876	< 1.01
		DolphinMixtral-8x7B	$0.008^{+9.588e-04}_{-9.102e-04}$	8744	7380	< 1.01
		Mixtral-8x7B	$-0.001^{+0.001}_{-0.002}$	8210	7765	< 1.01
		GPT-4o-mini	$0.001^{+4.719e-04}_{-4.619e-04}$	8142	6493	< 1.01
		Grok-4.1-fast	$0.002^{+8.871e-04}_{-9.345e-04}$	8391	5771	< 1.01
$\beta_{\mathrm{agr},2}$	Agreement bias strength Global Wealth	Llama-3.1-8B	$0.007^{+8.235e-04}_{-8.514e-04}$	7423	7008	< 1.01
		Qwen2.5-7B	$-7.740e-04^{+6.277e-04}_{-6.097e-04}$	7973	7458	< 1.01
		DolphinMixtral-8x7B	$0.005^{+6.461e-04}_{-6.878e-04}$	8522	7444	< 1.01
		Mixtral-8x7B	$0.019^{+0.002}_{-0.002}$	8110	7173	< 1.01
		GPT-4o-mini	$0.005^{+7.677e-04}_{-6.844e-04}$	7938	6799	< 1.01
		Grok-4.1-fast	$6.655e-04^{+5.483e-04}_{-5.526e-04}$	7725	7162	< 1.01
$\beta_{\mathrm{agr},3}$	Agreement bias strength AI	Llama-3.1-8B	$0.013^{+8.846e-04}_{-8.815e-04}$	7997	7091	< 1.01
		Qwen2.5-7B	$-0.008^{+7.631e-04}_{-8.295e-04}$	7834	7464	< 1.01
		DolphinMixtral-8x7B	$0.011^{+6.771e-04}_{-6.735e-04}$	7789	7201	< 1.01
		Mixtral-8x7B	$0.045^{+0.002}_{-0.002}$	8012	7203	< 1.01
		GPT-4o-mini	$-0.007^{+6.972e-04}_{-6.712e-04}$	8035	7103	< 1.01
		Grok-4.1-fast	$0.001^{+7.323e-04}_{-7.260e-04}$	8197	6739	< 1.01
$\beta_{\mathrm{agr},4}$	Agreement bias strength Music Preference	Llama-3.1-8B	$0.051^{+0.002}_{-0.002}$	7650	7752	< 1.01
		Qwen2.5-7B	$-0.008^{+0.001}_{-0.001}$	8330	6959	< 1.01
		DolphinMixtral-8x7B	$0.016^{+7.099e-04}_{-6.579e-04}$	8242	7245	< 1.01
		Mixtral-8x7B	$0.019^{+0.002}_{-0.002}$	8261	6715	< 1.01
		GPT-4o-mini	$0.003^{+0.002}_{-0.002}$	8586	6809	< 1.01
		Grok-4.1-fast	$-0.018^{+0.002}_{-0.002}$	8089	7068	< 1.01
$\beta_{\mathrm{agr},5}$	Agreement bias strength Vaccination	Llama-3.1-8B	$0.007^{+7.451e-04}_{-6.937e-04}$	7959	7599	< 1.01
		Qwen2.5-7B	$-0.032^{+0.002}_{-0.002}$	8223	7577	< 1.01
		DolphinMixtral-8x7B	$0.007^{+0.001}_{-0.001}$	8481	7239	< 1.01
		Mixtral-8x7B	$-3.524e-04^{+0.001}_{-0.001}$	7535	7516	< 1.01
		GPT-4o-mini	$0.002^{+5.417e-04}_{-5.327e-04}$	7937	7441	< 1.01

Continued on next page

Parameter	Description	Model	Posterior (median + HDI)	ESS_{bulk}	ESS_{tail}	$\hat{R}$
		Grok-4.1-fast	$-0.005^{+6.452e-04}_{-6.323e-04}$	7878	7401	< 1.01
$\beta_{agr,6}$	Agreement bias strength Abortion	Llama-3.1-8B	$0.009^{+8.764e-04}_{-8.579e-04}$	8234	7460	< 1.01
		Qwen2.5-7B	$-0.042^{+0.002}_{-0.002}$	8412	7681	< 1.01
		DolphinMixtral-8x7B	$0.01^{+7.422e-04}_{-7.171e-04}$	7431	6951	< 1.01
		Mixtral-8x7B	$0.023^{+0.002}_{-0.002}$	7964	7101	< 1.01
		GPT-4o-mini	$-5.621e-04^{+7.737e-04}_{-7.754e-04}$	8444	7574	< 1.01
		Grok-4.1-fast	$2.235e-04^{+4.397e-04}_{-4.662e-04}$	7512	6796	< 1.01
$\beta_{agr,7}$	Agreement bias strength Morality	Llama-3.1-8B	$0.029^{+0.001}_{-0.001}$	8281	7730	< 1.01
		Qwen2.5-7B	$-0.004^{+0.003}_{-0.003}$	7368	5026	< 1.01
		DolphinMixtral-8x7B	$-6.838e-05^{+0.002}_{-0.001}$	8193	7891	< 1.01
		Mixtral-8x7B	$-0.01^{+0.004}_{-0.004}$	7910	7131	< 1.01
		GPT-4o-mini	$-0.01^{+0.001}_{-0.001}$	8011	6613	< 1.01
		Grok-4.1-fast	$0.019^{+0.003}_{-0.003}$	8092	5246	< 1.01
$\beta_{agr,8}$	Agreement bias strength Food Preference	Llama-3.1-8B	$0.037^{+0.002}_{-0.002}$	7775	7048	< 1.01
		Qwen2.5-7B	$-0.019^{+0.002}_{-0.002}$	8174	6837	< 1.01
		DolphinMixtral-8x7B	$0.013^{+8.697e-04}_{-8.057e-04}$	8538	7097	< 1.01
		Mixtral-8x7B	$0.016^{+0.002}_{-0.002}$	8304	7138	< 1.01
		GPT-4o-mini	$0.004^{+0.001}_{-0.001}$	8805	7197	< 1.01
		Grok-4.1-fast	$-0.015^{+0.002}_{-0.002}$	8472	7236	< 1.01
$\beta_{agr,9}$	Agreement bias strength Earth Shape	Llama-3.1-8B	$0.013^{+9.044e-04}_{-9.950e-04}$	7751	7141	< 1.01
		Qwen2.5-7B	$-0.051^{+0.003}_{-0.003}$	7854	7311	< 1.01
		DolphinMixtral-8x7B	$0.005^{+9.313e-04}_{-8.509e-04}$	8586	6741	< 1.01
		Mixtral-8x7B	$0.009^{+0.002}_{-0.002}$	8151	6936	< 1.01
		GPT-4o-mini	$-8.532e-04^{+3.484e-04}_{-3.178e-04}$	7499	7612	< 1.01
		Grok-4.1-fast	$-8.833e-04^{+2.263e-04}_{-2.265e-04}$	7699	6644	< 1.01
$\beta_{agr,10}$	Agreement bias strength Social Media	Llama-3.1-8B	$0.025^{+7.532e-04}_{-7.493e-04}$	8165	7834	< 1.01
		Qwen2.5-7B	$-0.018^{+0.001}_{-0.001}$	7858	6981	< 1.01
		DolphinMixtral-8x7B	$0.014^{+8.792e-04}_{-8.913e-04}$	8633	7198	< 1.01
		Mixtral-8x7B	$0.037^{+0.003}_{-0.003}$	7865	6711	< 1.01
		GPT-4o-mini	$0.009^{+6.132e-04}_{-6.258e-04}$	7896	7501	< 1.01
		Grok-4.1-fast	$0.003^{+6.370e-04}_{-6.353e-04}$	8381	7371	< 1.01
$\beta_{agr,11}$	Agreement bias strength Free Will	Llama-3.1-8B	$0.018^{+6.831e-04}_{-6.379e-04}$	7650	7349	< 1.01
		Qwen2.5-7B	$-7.478e-04^{+0.002}_{-0.002}$	7879	7309	< 1.01
		DolphinMixtral-8x7B	$0.012^{+7.978e-04}_{-8.232e-04}$	8527	6799	< 1.01
		Mixtral-8x7B	$0.026^{+0.002}_{-0.002}$	8112	7138	< 1.01
		GPT-4o-mini	$-0.003^{+6.477e-04}_{-6.643e-04}$	8041	7171	< 1.01
		Grok-4.1-fast	$6.145e-05^{+6.629e-05}_{-6.655e-05}$	7508	6818	< 1.01
$\beta_{agr,12}$	Agreement bias strength Art Style	Llama-3.1-8B	$0.052^{+0.002}_{-0.002}$	8158	7305	< 1.01
		Qwen2.5-7B	$0.011^{+0.001}_{-0.001}$	8137	7601	< 1.01
		DolphinMixtral-8x7B	$0.018^{+7.635e-04}_{-8.380e-04}$	8174	6773	< 1.01
		Mixtral-8x7B	$0.047^{+0.002}_{-0.002}$	7918	7049	< 1.01
		GPT-4o-mini	$0.003^{+0.001}_{-0.001}$	8681	6488	< 1.01

Continued on next page

Parameter	Description	Model	Posterior (median + HDI)	ESS_{bulk}	ESS_{tail}	$\hat{R}$
		Grok-4.1-fast	$0.004^{+7.763e-04}_{-7.663e-04}$	7921	6389	< 1.01

Supplementary Table 9: **Detailed posterior summaries for topic-specific anchoring-bias strength parameter $\beta_{\text{anchor},k}$.** Posterior entries are medians with 95% HDIs.

Parameter	Description	Model	Posterior (median + HDI)	ESS_{bulk}	ESS_{tail}	$\hat{R}$
$\beta_{\text{anc},1}$	Anchoring bias strength Climate Change	Llama-3.1-8B	$-0.024^{+0.002}_{-0.002}$	7808	6445	< 1.01
		Qwen2.5-7B	$-0.029^{+0.003}_{-0.003}$	8419	6575	< 1.01
		DolphinMixtral-8x7B	$-0.006^{+0.004}_{-0.004}$	8186	7139	< 1.01
		Mixtral-8x7B	$-0.012^{+0.004}_{-0.005}$	8903	6762	< 1.01
		GPT-4o-mini	$-0.002^{+0.001}_{-0.001}$	8322	7051	< 1.01
		Grok-4.1-fast	$0.004^{+0.002}_{-0.002}$	7994	6783	< 1.01
$\beta_{\text{anc},2}$	Anchoring bias strength Global Wealth	Llama-3.1-8B	$-0.032^{+0.002}_{-0.002}$	8426	5645	< 1.01
		Qwen2.5-7B	$-0.012^{+0.002}_{-0.002}$	7993	7131	< 1.01
		DolphinMixtral-8x7B	$0.004^{+0.003}_{-0.003}$	8416	6897	< 1.01
		Mixtral-8x7B	$-0.037^{+0.004}_{-0.004}$	8323	7930	< 1.01
		GPT-4o-mini	$-0.011^{+0.002}_{-0.002}$	8965	7162	< 1.01
		Grok-4.1-fast	$-0.001^{+8.082e-04}_{-8.066e-04}$	8180	6716	< 1.01
$\beta_{\text{anc},3}$	Anchoring bias strength AI	Llama-3.1-8B	$-0.035^{+0.001}_{-0.001}$	8469	7123	< 1.01
		Qwen2.5-7B	$-0.002^{+0.002}_{-0.002}$	8101	7583	< 1.01
		DolphinMixtral-8x7B	$0.006^{+0.003}_{-0.003}$	9109	6852	< 1.01
		Mixtral-8x7B	$0.007^{+0.004}_{-0.004}$	8253	6692	< 1.01
		GPT-4o-mini	$-0.013^{+0.001}_{-0.001}$	8856	6960	< 1.01
		Grok-4.1-fast	$-0.006^{+0.001}_{-0.001}$	8354	7327	< 1.01
$\beta_{\text{anc},4}$	Anchoring bias strength Music Preference	Llama-3.1-8B	$-0.044^{+0.003}_{-0.003}$	8999	6710	< 1.01
		Qwen2.5-7B	$-0.059^{+0.003}_{-0.003}$	7681	6927	< 1.01
		DolphinMixtral-8x7B	$0.003^{+0.006}_{-0.005}$	8665	7047	< 1.01
		Mixtral-8x7B	$-0.015^{+0.005}_{-0.005}$	8146	5950	< 1.01
		GPT-4o-mini	$-0.011^{+0.004}_{-0.004}$	8076	5641	< 1.01
		Grok-4.1-fast	$-0.019^{+0.003}_{-0.003}$	7941	7053	< 1.01
$\beta_{\text{anc},5}$	Anchoring bias strength Vaccination	Llama-3.1-8B	$-0.029^{+0.001}_{-0.001}$	8006	6783	< 1.01
		Qwen2.5-7B	$-0.043^{+0.003}_{-0.003}$	8394	6902	< 1.01
		DolphinMixtral-8x7B	$1.030e-04^{+0.004}_{-0.004}$	9068	7229	< 1.01
		Mixtral-8x7B	$-0.01^{+0.004}_{-0.004}$	8598	7024	< 1.01
		GPT-4o-mini	$-0.006^{+0.001}_{-0.001}$	8287	7370	< 1.01
		Grok-4.1-fast	$-0.002^{+0.001}_{-0.001}$	8660	6555	< 1.01
$\beta_{\text{anc},6}$	Anchoring bias strength Abortion	Llama-3.1-8B	$-0.03^{+0.001}_{-0.001}$	8409	7106	< 1.01
		Qwen2.5-7B	$-0.06^{+0.004}_{-0.003}$	8395	7036	< 1.01
		DolphinMixtral-8x7B	$-0.004^{+0.002}_{-0.002}$	7980	7652	< 1.01
		Mixtral-8x7B	$-0.011^{+0.003}_{-0.003}$	7773	6756	< 1.01
		GPT-4o-mini	$0.001^{+0.001}_{-0.001}$	7568	7047	< 1.01
		Grok-4.1-fast	$2.948e-04^{+6.661e-04}_{-6.925e-04}$	8449	6970	< 1.01
		Llama-3.1-8B	$-0.048^{+0.002}_{-0.002}$	7883	6659	< 1.01

Continued on next page

Parameter	Description	Model	Posterior (median + HDI)	ESS_{bulk}	ESS_{tail}	$\hat{R}$
$\beta_{anc,7}$	Anchoring bias strength Morality	Qwen2.5-7B	$-0.003^{+0.005}_{-0.005}$	8671	6269	< 1.01
		DolphinMixtral-8x7B	$-0.013^{+0.008}_{-0.008}$	6494	6280	< 1.01
		Mixtral-8x7B	$-0.017^{+0.007}_{-0.007}$	8635	6725	< 1.01
		GPT-4o-mini	$-0.022^{+0.002}_{-0.002}$	8716	6505	< 1.01
		Grok-4.1-fast	$-0.032^{+0.006}_{-0.007}$	7907	7265	< 1.01
$\beta_{anc,8}$	Anchoring bias strength Food Preference	Llama-3.1-8B	$-0.054^{+0.003}_{-0.003}$	8608	7320	< 1.01
		Qwen2.5-7B	$-0.009^{+0.004}_{-0.003}$	8005	6096	< 1.01
		DolphinMixtral-8x7B	$-0.013^{+0.004}_{-0.004}$	7924	7273	< 1.01
		Mixtral-8x7B	$-0.026^{+0.006}_{-0.006}$	9082	6194	< 1.01
		GPT-4o-mini	$-0.028^{+0.002}_{-0.002}$	8548	6906	< 1.01
		Grok-4.1-fast	$-0.026^{+0.004}_{-0.003}$	7802	7908	< 1.01
$\beta_{anc,9}$	Anchoring bias strength Earth Shape	Llama-3.1-8B	$-0.026^{+0.002}_{-0.002}$	7685	5858	< 1.01
		Qwen2.5-7B	$-0.056^{+0.005}_{-0.005}$	8549	6138	< 1.01
		DolphinMixtral-8x7B	$-0.001^{+0.003}_{-0.003}$	8785	6850	< 1.01
		Mixtral-8x7B	$-0.017^{+0.004}_{-0.004}$	8488	6867	< 1.01
		GPT-4o-mini	$-0.002^{+4.934e-04}_{-5.294e-04}$	8245	7122	< 1.01
		Grok-4.1-fast	$-2.447e-05^{+3.321e-04}_{-3.164e-04}$	8036	6514	< 1.01
$\beta_{anc,10}$	Anchoring bias strength Social Media	Llama-3.1-8B	$-0.043^{+0.001}_{-0.001}$	8258	7368	< 1.01
		Qwen2.5-7B	$-0.051^{+0.002}_{-0.002}$	7851	7158	< 1.01
		DolphinMixtral-8x7B	$0.006^{+0.004}_{-0.004}$	8497	6758	< 1.01
		Mixtral-8x7B	$-0.017^{+0.006}_{-0.006}$	8106	6731	< 1.01
		GPT-4o-mini	$-0.023^{+0.001}_{-0.001}$	8440	7710	< 1.01
		Grok-4.1-fast	$-0.004^{+9.375e-04}_{-0.001}$	8133	7301	< 1.01
$\beta_{anc,11}$	Anchoring bias strength Free Will	Llama-3.1-8B	$-0.022^{+0.001}_{-0.001}$	8145	7298	< 1.01
		Qwen2.5-7B	$-0.069^{+0.003}_{-0.003}$	8125	7728	< 1.01
		DolphinMixtral-8x7B	$-0.001^{+0.003}_{-0.003}$	9114	7412	< 1.01
		Mixtral-8x7B	$-0.015^{+0.003}_{-0.003}$	9193	6897	< 1.01
		GPT-4o-mini	$-0.021^{+0.001}_{-0.001}$	8331	7452	< 1.01
		Grok-4.1-fast	$-1.554e-04^{+9.929e-05}_{-1.007e-04}$	7742	6263	< 1.01
$\beta_{anc,12}$	Anchoring bias strength Art Style	Llama-3.1-8B	$-0.043^{+0.003}_{-0.003}$	8078	6878	< 1.01
		Qwen2.5-7B	$-0.036^{+0.003}_{-0.003}$	8184	6469	< 1.01
		DolphinMixtral-8x7B	$5.932e-04^{+0.004}_{-0.004}$	8149	6815	< 1.01
		Mixtral-8x7B	$-0.012^{+0.006}_{-0.006}$	8980	6268	< 1.01
		GPT-4o-mini	$-0.025^{+0.002}_{-0.002}$	8676	7395	< 1.01
		Grok-4.1-fast	$-0.004^{+0.001}_{-0.001}$	8297	6830	< 1.01

Supplementary Table 10: **Detailed posterior summaries for topic-specific baseline standard deviation parameter $\sigma_{0,k}$.** Posterior entries are medians with 95% HDIs.

Parameter	Description	Model	Posterior (median + HDI)	ESS_{bulk}	ESS_{tail}	$\hat{R}$
$\sigma_{0,1}$	Baseline SD Climate Change	Llama-3.1-8B	$0.116^{+0.002}_{-0.002}$	7690	6721	< 1.01
		Qwen2.5-7B	$0.204^{+0.003}_{-0.003}$	8057	6922	< 1.01
		DolphinMixtral-8x7B	$0.395^{+0.006}_{-0.006}$	8208	6179	< 1.01

Continued on next page

Parameter	Description	Model	Posterior (median + HDI)	ESS_{bulk}	ESS_{tail}	$\hat{R}$
		Mixtral-8x7B	$0.284^{+0.004}_{-0.004}$	7663	5873	< 1.01
		GPT-4o-mini	$0.109^{+0.002}_{-0.002}$	8255	7433	< 1.01
		Grok-4.1-fast	$0.225^{+0.003}_{-0.003}$	7432	5168	< 1.01
$\sigma_{0,2}$	Baseline SD Global Wealth	Llama-3.1-8B	$0.183^{+0.003}_{-0.003}$	8212	5990	< 1.01
		Qwen2.5-7B	$0.114^{+0.003}_{-0.003}$	8275	7340	< 1.01
		DolphinMixtral-8x7B	$0.268^{+0.005}_{-0.005}$	8153	6588	< 1.01
		Mixtral-8x7B	$0.347^{+0.005}_{-0.005}$	7575	5689	< 1.01
		GPT-4o-mini	$0.166^{+0.002}_{-0.002}$	8028	6173	< 1.01
		Grok-4.1-fast	$0.125^{+0.002}_{-0.002}$	8141	6400	< 1.01
$\sigma_{0,3}$	Baseline SD AI	Llama-3.1-8B	$0.156^{+0.002}_{-0.003}$	8076	6039	< 1.01
		Qwen2.5-7B	$0.045^{+0.002}_{-0.002}$	7784	6674	< 1.01
		DolphinMixtral-8x7B	$0.243^{+0.005}_{-0.005}$	8428	6474	< 1.01
		Mixtral-8x7B	$0.327^{+0.004}_{-0.005}$	7806	5686	< 1.01
		GPT-4o-mini	$0.134^{+0.002}_{-0.002}$	8496	6519	< 1.01
		Grok-4.1-fast	$0.192^{+0.003}_{-0.002}$	7990	5981	< 1.01
$\sigma_{0,4}$	Baseline SD Music Preference	Llama-3.1-8B	$0.302^{+0.005}_{-0.005}$	7771	6553	< 1.01
		Qwen2.5-7B	$0.078^{+0.001}_{-0.001}$	7915	7199	< 1.01
		DolphinMixtral-8x7B	$0.204^{+0.005}_{-0.005}$	8363	6682	< 1.01
		Mixtral-8x7B	$0.31^{+0.005}_{-0.005}$	7911	5483	< 1.01
		GPT-4o-mini	$0.244^{+0.004}_{-0.004}$	7732	6561	< 1.01
		Grok-4.1-fast	$0.458^{+0.006}_{-0.006}$	7947	6607	< 1.01
$\sigma_{0,5}$	Baseline SD Vaccination	Llama-3.1-8B	$0.118^{+0.002}_{-0.002}$	7378	6636	< 1.01
		Qwen2.5-7B	$0.297^{+0.004}_{-0.005}$	7489	6644	< 1.01
		DolphinMixtral-8x7B	$0.427^{+0.006}_{-0.006}$	8302	6045	< 1.01
		Mixtral-8x7B	$0.273^{+0.004}_{-0.004}$	8110	6056	< 1.01
		GPT-4o-mini	$0.123^{+0.002}_{-0.002}$	8304	6272	< 1.01
		Grok-4.1-fast	$0.158^{+0.002}_{-0.002}$	7330	5361	< 1.01
$\sigma_{0,6}$	Baseline SD Abortion	Llama-3.1-8B	$0.163^{+0.003}_{-0.003}$	8132	6435	< 1.01
		Qwen2.5-7B	$0.405^{+0.006}_{-0.006}$	8508	6818	< 1.01
		DolphinMixtral-8x7B	$0.287^{+0.005}_{-0.004}$	8420	6911	< 1.01
		Mixtral-8x7B	$0.345^{+0.006}_{-0.005}$	7552	6024	< 1.01
		GPT-4o-mini	$0.17^{+0.002}_{-0.002}$	8114	6222	< 1.01
		Grok-4.1-fast	$0.103^{+0.001}_{-0.001}$	7886	6965	< 1.01
$\sigma_{0,7}$	Baseline SD Morality	Llama-3.1-8B	$0.223^{+0.003}_{-0.004}$	8003	5978	< 1.01
		Qwen2.5-7B	$0.16^{+0.004}_{-0.003}$	8231	6740	< 1.01
		DolphinMixtral-8x7B	$0.42^{+0.007}_{-0.007}$	8280	6579	< 1.01
		Mixtral-8x7B	$0.625^{+0.009}_{-0.009}$	8194	6173	< 1.01
		GPT-4o-mini	$0.209^{+0.003}_{-0.003}$	7914	6517	< 1.01
		Grok-4.1-fast	$0.753^{+0.01}_{-0.01}$	7235	6568	< 1.01
$\sigma_{0,8}$	Baseline SD Food Preference	Llama-3.1-8B	$0.32^{+0.005}_{-0.005}$	8142	6475	< 1.01
		Qwen2.5-7B	$0.232^{+0.004}_{-0.004}$	7877	6332	< 1.01
		DolphinMixtral-8x7B	$0.285^{+0.005}_{-0.005}$	7949	6564	< 1.01
		Mixtral-8x7B	$0.317^{+0.005}_{-0.005}$	7708	5400	< 1.01
		GPT-4o-mini	$0.25^{+0.004}_{-0.004}$	7658	6929	< 1.01
		Grok-4.1-fast	$0.453^{+0.006}_{-0.006}$	7911	6326	< 1.01
$\sigma_{0,9}$	Baseline SD Earth Shape	Llama-3.1-8B	$0.204^{+0.003}_{-0.003}$	7998	6376	< 1.01
		Qwen2.5-7B	$0.628^{+0.009}_{-0.008}$	8072	7537	< 1.01
		DolphinMixtral-8x7B	$0.377^{+0.006}_{-0.005}$	7810	5780	< 1.01

Continued on next page

Parameter	Description	Model	Posterior (median + HDI)	ESS_{bulk}	ESS_{tail}	$\hat{R}$
		Mixtral-8x7B	$0.369^{+0.006}_{-0.007}$	7941	5644	< 1.01
		GPT-4o-mini	$0.072^{+0.001}_{-9.363e-04}$	7370	7186	< 1.01
		Grok-4.1-fast	$0.05^{+6.052e-04}_{-6.216e-04}$	8503	6351	< 1.01
$\sigma_{0,10}$	Baseline SD Social Media	Llama-3.1-8B	$0.154^{+0.002}_{-0.003}$	8105	5494	< 1.01
		Qwen2.5-7B	$0.225^{+0.004}_{-0.003}$	8250	6285	< 1.01
		DolphinMixtral-8x7B	$0.344^{+0.006}_{-0.006}$	8154	6431	< 1.01
		Mixtral-8x7B	$0.396^{+0.007}_{-0.007}$	8047	5755	< 1.01
		GPT-4o-mini	$0.148^{+0.002}_{-0.002}$	7384	6312	< 1.01
		Grok-4.1-fast	$0.147^{+0.002}_{-0.002}$	8129	6240	< 1.01
$\sigma_{0,11}$	Baseline SD Free Will	Llama-3.1-8B	$0.15^{+0.002}_{-0.002}$	7681	6339	< 1.01
		Qwen2.5-7B	$0.326^{+0.005}_{-0.005}$	8173	6611	< 1.01
		DolphinMixtral-8x7B	$0.316^{+0.005}_{-0.005}$	8444	6397	< 1.01
		Mixtral-8x7B	$0.331^{+0.005}_{-0.005}$	7089	5670	< 1.01
		GPT-4o-mini	$0.145^{+0.002}_{-0.002}$	7510	4986	< 1.01
		Grok-4.1-fast	$0.015^{+1.903e-04}_{-1.816e-04}$	8036	6683	< 1.01
$\sigma_{0,12}$	Baseline SD Art Style	Llama-3.1-8B	$0.289^{+0.005}_{-0.004}$	8023	6634	< 1.01
		Qwen2.5-7B	$0.124^{+0.002}_{-0.003}$	8057	6972	< 1.01
		DolphinMixtral-8x7B	$0.263^{+0.005}_{-0.005}$	8321	6990	< 1.01
		Mixtral-8x7B	$0.396^{+0.006}_{-0.006}$	7129	6080	< 1.01
		GPT-4o-mini	$0.215^{+0.003}_{-0.003}$	8210	6685	< 1.01
		Grok-4.1-fast	$0.176^{+0.002}_{-0.002}$	7896	6250	< 1.01

Supplementary Table 11: **Detailed posterior summaries for the climate-only model with initial-condition-specific topic attractors.** Posterior entries are medians with 95% HDIs.

Parameter	Description	Model	Posterior (median + HDI)	ESS_{bulk}	ESS_{tail}	$\hat{R}$
α_{int}	Interaction strength	Llama-3.1-8B	$0.026^{+0.004}_{-0.004}$	5206	5368	< 1.01
		Qwen2.5-7B	$0.063^{+0.005}_{-0.006}$	6101	5071	< 1.01
		DolphinMixtral-8x7B	$0.036^{+0.008}_{-0.009}$	2986	2844	< 1.01
		Mixtral-8x7B	$0.045^{+0.012}_{-0.012}$	5540	5819	< 1.01
		CustomMixtral-8x7B	$0.119^{+0.011}_{-0.011}$	6080	5950	< 1.01
		GPT-4o-mini	$-0.002^{+0.003}_{-0.003}$	4158	4751	< 1.01
τ_{int}	Interaction decay timescale	Llama-3.1-8B	$0.265^{+0.185}_{-0.177}$	8692	4741	< 1.01
		Qwen2.5-7B	$0.32^{+0.221}_{-0.206}$	6041	4116	< 1.01
		DolphinMixtral-8x7B	$36.315^{+66.364}_{-26.994}$	8918	6159	< 1.01
		Mixtral-8x7B	$2.555^{+1.809}_{-1.132}$	7418	4383	< 1.01
		CustomMixtral-8x7B	$0.689^{+0.199}_{-0.174}$	8187	6267	< 1.01
		GPT-4o-mini	$2.055^{+10.726}_{-1.965}$	6383	5681	< 1.01
β_{top}	Topic bias strength	Llama-3.1-8B	$0.24^{+0.005}_{-0.005}$	5585	5867	< 1.01
		Qwen2.5-7B	$0.279^{+0.013}_{-0.012}$	2550	4622	< 1.01
		DolphinMixtral-8x7B	$0.139^{+0.012}_{-0.012}$	5176	5162	< 1.01
		Mixtral-8x7B	$0.204^{+0.015}_{-0.014}$	5082	4679	< 1.01
		CustomMixtral-8x7B	$0.39^{+0.02}_{-0.021}$	4747	5184	< 1.01
		GPT-4o-mini	$0.029^{+0.004}_{-0.004}$	4709	5452	< 1.01
		Llama-3.1-8B	$0.399^{+0.062}_{-0.07}$	9811	4286	< 1.01
		Qwen2.5-7B	$0.486^{+0.14}_{-0.15}$	8008	4283	< 1.01
		DolphinMixtral-8x7B	$7.527^{+4.416}_{-2.644}$	3210	3825	< 1.01

Continued on next page

Parameter	Description	Model	Posterior (median + HDI)	ESS_{bulk}	ESS_{tail}	$\hat{R}$
τ_{top}	Topic-bias decay timescale	Mixtral-8x7B	$15.147^{+10.321}_{-5.932}$	5916	4214	< 1.01
		CustomMixtral-8x7B	$2.846^{+0.317}_{-0.294}$	6228	5003	< 1.01
		GPT-4o-mini	$3.158^{+1.051}_{-0.779}$	6912	5511	< 1.01
$b^{(-2)}_{climate}$	Climate topic attractor for initial opinion -2	Llama-3.1-8B	$1.961^{+0.039}_{-0.047}$	2876	2454	< 1.01
		Qwen2.5-7B	$1.898^{+0.065}_{-0.069}$	2701	4247	< 1.01
		DolphinMixtral-8x7B	$0.894^{+0.158}_{-0.165}$	8250	6092	< 1.01
		Mixtral-8x7B	$1.292^{+0.072}_{-0.067}$	1e+04	6422	< 1.01
		CustomMixtral-8x7B	$-0.282^{+0.09}_{-0.083}$	6748	6190	< 1.01
		GPT-4o-mini	$1.41^{+0.25}_{-0.246}$	7371	4103	< 1.01
$b^{(-1)}_{climate}$	Climate topic attractor for initial opinion -1	Llama-3.1-8B	$1.95^{+0.047}_{-0.049}$	3405	3190	< 1.01
		Qwen2.5-7B	$1.956^{+0.044}_{-0.057}$	2133	2181	< 1.01
		DolphinMixtral-8x7B	$0.932^{+0.164}_{-0.155}$	8277	5906	< 1.01
		Mixtral-8x7B	$1.466^{+0.07}_{-0.068}$	1e+04	6506	< 1.01
		CustomMixtral-8x7B	$1.095^{+0.071}_{-0.075}$	8186	6404	< 1.01
		GPT-4o-mini	$1.949^{+0.051}_{-0.129}$	6232	3859	< 1.01
$b^{(0)}_{climate}$	Climate topic attractor for initial opinion 0	Llama-3.1-8B	$1.09^{+0.054}_{-0.051}$	1e+04	5749	< 1.01
		Qwen2.5-7B	$1.931^{+0.046}_{-0.05}$	2863	2238	< 1.01
		DolphinMixtral-8x7B	$1.711^{+0.212}_{-0.191}$	3225	1912	< 1.01
		Mixtral-8x7B	$1.721^{+0.074}_{-0.075}$	9343	5886	< 1.01
		CustomMixtral-8x7B	$1.243^{+0.068}_{-0.062}$	8139	6632	< 1.01
		GPT-4o-mini	$1.995^{+0.005}_{-0.017}$	6507	3470	< 1.01
$b^{(+1)}_{climate}$	Climate topic attractor for initial opinion +1	Llama-3.1-8B	$1.98^{+0.02}_{-0.028}$	4146	2741	< 1.01
		Qwen2.5-7B	$1.986^{+0.014}_{-0.029}$	4743	2928	< 1.01
		DolphinMixtral-8x7B	$1.55^{+0.208}_{-0.177}$	4459	4504	< 1.01
		Mixtral-8x7B	$1.947^{+0.053}_{-0.059}$	4540	2467	< 1.01
		CustomMixtral-8x7B	$1.444^{+0.059}_{-0.06}$	8643	6649	< 1.01
		GPT-4o-mini	$1.726^{+0.253}_{-0.234}$	4681	2054	< 1.01
$b^{(+2)}_{climate}$	Climate topic attractor for initial opinion +2	Llama-3.1-8B	$1.973^{+0.027}_{-0.03}$	4049	2414	< 1.01
		Qwen2.5-7B	$1.874^{+0.051}_{-0.05}$	8755	4744	< 1.01
		DolphinMixtral-8x7B	$1.409^{+0.179}_{-0.175}$	5580	5154	< 1.01
		Mixtral-8x7B	$1.981^{+0.019}_{-0.043}$	5894	3524	< 1.01
		CustomMixtral-8x7B	$1.814^{+0.059}_{-0.059}$	7994	5466	< 1.01
		GPT-4o-mini	$1.062^{+0.243}_{-0.251}$	1e+04	5037	< 1.01
β_{agr}	Agreement bias strength	Llama-3.1-8B	$0.021^{+0.003}_{-0.003}$	3590	5107	< 1.01
		Qwen2.5-7B	$-0.007^{+0.004}_{-0.004}$	2340	4361	< 1.01
		DolphinMixtral-8x7B	$0.032^{+0.004}_{-0.004}$	5259	6137	< 1.01
		Mixtral-8x7B	$-0.024^{+0.003}_{-0.004}$	6286	4976	< 1.01
		CustomMixtral-8x7B	$-0.063^{+0.006}_{-0.006}$	6624	6164	< 1.01
		GPT-4o-mini	$0.003^{+0.002}_{-0.002}$	6264	5779	< 1.01
τ_{agr}	Agreement-bias decay timescale	Llama-3.1-8B	$0.186^{+0.108}_{-0.104}$	8477	4625	< 1.01
		Qwen2.5-7B	$0.475^{+0.423}_{-0.355}$	6575	5667	< 1.01
		DolphinMixtral-8x7B	$101.973^{+160.537}_{-72.191}$	9522	5110	< 1.01
		Mixtral-8x7B	$22.421^{+37.543}_{-15.418}$	7268	5089	< 1.01
		CustomMixtral-8x7B	$2.717^{+0.541}_{-0.453}$	6200	5155	< 1.01

Continued on next page

Parameter	Description	Model	Posterior (median + HDI)	ESS_{bulk}	ESS_{tail}	$\hat{R}$
		GPT-4o-mini	$2.258^{+6.802}_{-2.063}$	6665	5271	< 1.01
β_{anc}	Anchoring bias strength	Llama-3.1-8B	$-0.061^{+0.005}_{-0.005}$	5697	5964	< 1.01
		Qwen2.5-7B	$-0.098^{+0.007}_{-0.007}$	5966	6304	< 1.01
		DolphinMixtral-8x7B	$-0.024^{+0.024}_{-0.017}$	2526	985	< 1.01
		Mixtral-8x7B	$-0.034^{+0.013}_{-0.015}$	5893	5509	< 1.01
		CustomMixtral-8x7B	$-0.121^{+0.013}_{-0.013}$	6343	5684	< 1.01
		GPT-4o-mini	$-0.009^{+0.004}_{-0.004}$	4801	5156	< 1.01
τ_{anc}	Anchoring-bias decay timescale	Llama-3.1-8B	$0.24^{+0.116}_{-0.136}$	6915	3745	< 1.01
		Qwen2.5-7B	$0.298^{+0.129}_{-0.155}$	5391	3465	< 1.01
		DolphinMixtral-8x7B	$0.307^{+1.272}_{-0.281}$	3036	870	< 1.01
		Mixtral-8x7B	$5.815^{+13.616}_{-4.318}$	6065	5029	< 1.01
		CustomMixtral-8x7B	$0.26^{+0.159}_{-0.16}$	7322	4914	< 1.01
		GPT-4o-mini	$2.166^{+2.565}_{-1.316}$	5488	4181	< 1.01
σ_0	Baseline SD	Llama-3.1-8B	$0.069^{+0.002}_{-0.002}$	5505	6257	< 1.01
		Qwen2.5-7B	$0.111^{+0.004}_{-0.004}$	4037	5154	< 1.01
		DolphinMixtral-8x7B	$0.383^{+0.01}_{-0.009}$	4952	4898	< 1.01
		Mixtral-8x7B	$0.263^{+0.004}_{-0.004}$	1e+04	5851	< 1.01
		CustomMixtral-8x7B	$0.303^{+0.005}_{-0.005}$	8995	6457	< 1.01
		GPT-4o-mini	$0.1^{+0.002}_{-0.002}$	7770	6170	< 1.01
ϵ	Entropy contribution to SD	Llama-3.1-8B	$0.081^{+0.003}_{-0.003}$	5363	5805	< 1.01
		Qwen2.5-7B	$1.776^{+0.198}_{-0.185}$	4066	5034	< 1.01
		DolphinMixtral-8x7B	$0.023^{+0.004}_{-0.004}$	4930	4861	< 1.01
		Mixtral-8x7B	$0.788^{+0.106}_{-0.102}$	1e+04	6348	< 1.01
		CustomMixtral-8x7B	$0.156^{+0.009}_{-0.009}$	8602	4922	< 1.01
		GPT-4o-mini	$0.807^{+0.05}_{-0.053}$	7518	5919	< 1.01

Supplementary References

1. Abril-Pla, O. *et al.* PyMC: a modern, and comprehensive probabilistic programming framework in Python. *PeerJ Computer Science* **9,** e1516 (2023).
2. Hoffman, M. D., Gelman, A., *et al.* The No-U-Turn sampler: adaptively setting path lengths in Hamiltonian Monte Carlo. *J. Mach. Learn. Res.* **15,** 1593–1623 (2014).
3. Martin, O. A. *et al.* ArviZ: a modular and flexible library for exploratory analysis of Bayesian models. *Journal of Open Source Software* (2026).
4. Vehtari, A., Gelman, A., Goodrich, B. & Gabry, J. *Bayesian R2 and LOO-R2* 2019. https://avehtari.github.io/bayes_R2/bayes_R2.html.
5. Schoppe, O., Harper, N. S., Willmore, B. D., King, A. J. & Schnupp, J. W. Measuring the performance of neural models. *Frontiers in computational neuroscience* **10,** 10 (2016).
6. Nili, H. *et al.* A toolbox for representational similarity analysis. *PLoS computational biology* **10,** e1003553 (2014).
7. Cover, T. M. *Elements of information theory* (John Wiley & Sons, 1999).
8. Hu, E. J. *et al.* Lora: Low-rank adaptation of large language models. *ICLR* **1,** 3 (2022).
9. Meta. *Llama-3.1-8B-Instruct* Hugging Face model card. Model release date July 23, 2024; accessed 2026-04-08. 2024. https://huggingface.co/meta-llama/Llama-3.1-8B-Instruct.
10. Qwen Team. *Qwen2.5-LLM: Extending the boundary of LLMs* Qwen blog. Published 2024-09-19; accessed 2026-04-08. Sept. 2024. https://qwenlm.github.io/blog/qwen2.5-llm/.
11. TheBloke. *dolphin-2.7-mixtral-8x7b-AWQ* Hugging Face model card. Accessed 2026-04-08. 2024. https://huggingface.co/TheBloke/dolphin-2.7-mixtral-8x7b-AWQ.
12. TheBloke. *Upload README.md: dolphin-2.7-mixtral-8x7b-AWQ* Hugging Face commit page. Public commit dated 2024-01-01; accessed 2026-04-08. Jan. 2024. https://huggingface.co/TheBloke/dolphin-2.7-mixtral-8x7b-AWQ/commit/4a05261e95d92ef879bf6472e329f010adb5af75.
13. Hugging Quants. *Mixtral-8x7B-Instruct-v0.1-AWQ-INT4* Hugging Face model card. Accessed 2026-04-08. 2024. https://huggingface.co/hugging-quants/Mixtral-8x7B-Instruct-v0.1-AWQ-INT4.
14. Hugging Quants. *Initial commit: Mixtral-8x7B-Instruct-v0.1-AWQ-INT4* Hugging Face commit page. Public commit dated 2024-10-05; accessed 2026-04-08. Oct. 2024. https://huggingface.co/hugging-quants/Mixtral-8x7B-Instruct-v0.1-AWQ-INT4/commit/48770cbab1866c34a0e11a57557f2ff9dc77d360.
15. Jiang, A. Q. *et al.* Mixtral of experts. *arXiv preprint arXiv:2401.04088* (2024).
16. OpenAI. *GPT-4o mini: advancing cost-efficient intelligence* OpenAI. Published 2024-07-18; accessed 2026-04-08. July 2024. https://openai.com/index/gpt-4o-mini-advancing-cost-efficient-intelligence/.
17. OpenAI. *GPT-4o mini model documentation* OpenAI developer documentation. Accessed 2026-04-08. 2026. https://developers.openai.com/api/docs/models/gpt-4o-mini.
18. xAI. *Grok 4.1 Model Card* xAI model card. Published 2025-11-17; accessed 2026-04-08. Nov. 2025. https://data.x.ai/2025-11-17-grok-4-1-model-card.pdf.
19. xAI. *grok-4-1-fast-non-reasoning* xAI developer documentation. Accessed 2026-04-08. 2026. https://docs.x.ai/developers/models/grok-4-1-fast-non-reasoning.
20. xAI. *Models and Pricing* xAI developer documentation. Accessed 2026-04-08. 2026. https://docs.x.ai/developers/models.
21. Arena Intelligence. *Text Arena Overall leaderboard* Public leaderboard. Accessed 2026-04-08; scores are dynamic and may change over time. 2026. https://arena.ai/leaderboard/text/overall-no-style-control.
