# Supplementary material for: Disentangling interaction and bias effects in opinion dynamics of large language models
Source: Nat Commun. 2026 Sep 22;17:10077. doi: 10.1038/s41467-026-77340-3 (PMC13597526; doi:10.1038/s41467-026-77340-3)
Supplement: Supplementary file 2 [file 41467_2026_77340_MOESM2_ESM.pdf]

nature portfolio

Peer Review File

Disentangling Interaction and Bias Effects in Opinion Dynamics of Large Language Models

Corresponding Author: Mr Vincent Brockers

This file contains all reviewer reports in order by version, followed by all author rebuttals in order by version.

Version 0:

Reviewer comments:

Reviewer #1

(Remarks to the Author)
This manuscript introduces a Bayesian framework to decompose opinion change in dyadic LLM debates into interaction and three biases (topic, agreement, anchoring), reporting rapid convergence to topic-dependent attractors, small sustained interaction effects, and an entropy–susceptibility link, plus a narrow fine-tuning case study.

Major issue: The authors provide no validation that their method can recover known parameters from synthetic data. This is particularly concerning given evidence of identifiability problems in their results: GPT-4o-mini's agreement bias parameters are either zero-constrained ($\beta\_agree = 0.0 \pm 0.0$) or completely unconstrained ($b\_agree = 0.0 \pm 2.0$), some parameters show poor convergence, and the model structure creates severe collinearity when multiple bias terms pull toward similar attractors. Without demonstrating that the framework can correctly decompose effects when ground truth is known, the reader lacks sufficient evidence that could warrant their adoption of the proposed approach.

Standard practice for methods papers requires simulation studies showing parameter recovery across diverse scenarios (in this particular case: interaction-dominated, single-bias-dominated, competing biases). The authors should also test whether the framework incorrectly infers biases when applied to data generated with interaction alone, and explicitly characterize conditions under which decomposition is/isn't reliable.

Minor issues:
1. the modeling choices (one-sided prior ruling out negative agreement; fixed exponential decay with restrictive prior on the time constant; asymmetric anchoring applied only to the responder) narrow the hypothesis space in ways that can predetermine qualitative conclusions (e.g., "fast decisions"), yet sensitivity checks to alternative links/priors and order-balancing are limited.
2. While the paper positions this work as relevant to "using LLMs as proxies for human behavior" (abstract), no comparison to human opinion dynamics data is provided. Without demonstrating that identified biases, effect sizes, and interaction timescales align with human studies, claims about human relevance remain unvalidated.

Independent of the decomposition, several observations (biases dominating over interaction; quick convergence; entropy predicting susceptibility) are interesting but do not require the full framework and are already suggested by recent literature. The topical/model scope is narrow, robustness to decoding/prompt initialization and seeds is under-reported, and there is no human baseline to contextualize effect sizes or a downstream demonstration that the decomposition improves debate truthfulness or LLM-as-judge reliability.

The work addresses an important question and the framework concept has merit for the emerging field of LLM agent-based modeling, but the provided evidence doesn't support the quantitative decomposition claims that constitute the paper's primary novelty.

(Remarks on code availability)

Reviewer #2

(Remarks to the Author)
This manuscript presents an original and carefully motivated contribution to the study of LLMs in interactive, multi-agent settings. By introducing a Bayesian framework to quantify and distinguish interaction effects from model-internal biases, the authors address an important and timely question at the intersection of AI and social behavior simulation. The approach offers a structured way to analyze how LLMs' opinions evolve in dialogue and to separate genuine influence from systematic biases such as topic alignment, agreement tendency, and anchoring. This focus makes the paper conceptually strong and relevant to ongoing discussions about the interpretability and behavior of generative language models.

The paper's overall structure and narrative are clear and well-motivated. The introduction provides a convincing rationale for why interpretability and decomposition of LLM-driven opinion dynamics are necessary, given the widespread use of such models as proxies for human-like discussion and debate. The integration of Bayesian methods adds coherence and interpretability to the analysis, offering a principled framework for comparing models and topics. The decision to evaluate several distinct LLMs and to demonstrate how each shows a characteristic bias profile strengthens the empirical contribution and enhances the general interest of the work.

The work is both timely and relevant. As LLMs are increasingly used in simulations of dialogue, persuasion, and social interaction, developing rigorous ways to characterize their internal biases and influence patterns is an important step forward. The proposed framework provides a foundation for more systematic investigations of such phenomena and may inform both applied and theoretical research in AI and related disciplines. Overall, this paper represents a valuable and well-executed contribution to an emerging area of study.

A minor point that could be clarified concerns the role of the short "chain-of-thought" texts used to initialize each agent. These texts appear to establish the initial reasoning and stance from which the agents begin their discussions, yet the manuscript does not present or analyze them in detail. Because these texts effectively shape how each agent interprets and frames the topic, variation in their content or tone could influence the agents' subsequent responses and the overall opinion shifts observed. Providing a few illustrative examples or a brief examination of their consistency would help readers better understand how the initialization process might contribute to the reported effects and strengthen the transparency of the study's design.

(Remarks on code availability)

Reviewer #3

(Remarks to the Author)
This manuscript examines opinion dynamics among LLM agents through a Bayesian modeling framework, and disentangle the relative contribution of the effect of interaction between agents and three intrinsic biases – topic, agreement and anchoring – during simulated discussions. Overall, the research is well conducted, with clearly documented procedure and analysis. It contributes to the literature by quantifying the effects through a mathematical framework. Below are some comments for authors to consider when revising this manuscript.

Understand that the interaction effect represents an ongoing social influence between two agents who are currently disagreeing, while the anchoring bias applies to the second agent when exposed to the first agent's initial opinion. However, the anchoring bias could be seen as a special case (first round) of an interaction effect when the second agent first joins the conversation, as both lie on the continuum of social influence mechanisms, differing primarily in timing and symmetry. Re-consider these two concepts or a brief clarification of this relationship would help strengthen theoretical coherence.

The results differ across topics, but the implications of these variations are not fully discussed. In addition, since only one topic (climate change) was used for fine-tuning, the authors may want to clarify whether the observed changes are topic-specific or reflect generalizable tendencies.

The description of the simulation procedure could specify how stochasticity is introduced in the Chain-of-Thought prompts and whether each run is seeded differently.

The term "topic bias shift" may be a bit misleading in conceptual terms. It intends to reflect a pulling force from an assigned role back toward the model's default stance, regardless of whether that default arises from data-driven bias. In other words, as long as the assigned role is different from the default opinion, the pulling effect will occur. Thus, the focus here is not the topic bias, but rather the influence of the prior stance. Given this, the authors could consider using a more neutral term such as "default effect" or "topic prior effect", or add a note justifying the choice of "Topic Bias" over a neutral term.

The use of an exponential decay function for interaction influence is standard in opinion dynamics models. However, the paper itself notes that the inferred decay is "unrealistically fast" and "unrealistically low" compared to humans (p6), and that fine-tuning yielded only modest improvement. It would be valuable to discuss the implications of this rapid decay. For instance, whether it limits the model's comparability to human data, or whether the effect is intrinsic to LLM interaction patterns rather than a methodological artifact.

Since Mixtral-8x7b and GPT-4o-mini showed a negative anchoring effect, the discussion could briefly interpret this counter-

intuitive result. Does it suggest a rejection bias against the first statement heard?

Mixtral-8x7b and GPT-4o-mini exhibit a small negative anchoring effect, which is counter-intuitive. A short discussion of this finding would be helpful. Does it indicate a contrastive or rejection bias against the first-stated opinion, or might it result from constraints in the model fitting?

A few minor comments:
Figure 5 (p. 7): Remove the duplicated "the" in the third-to-last line of the caption.
Tables: Ensure consistent use of decimal places across parameter estimates.

(Remarks on code availability)

Reviewer #4

(Remarks to the Author)
This is a thoughtful, well-structured study with a clear methodological contribution and commendable transparency. Addressing the points below, especially scope, measurement, and posterior diagnostics, would materially strengthen the work.

Five main disadvantages
1. Limited external validity: Findings are drawn from a small set of models, constraining how broad conclusions about LLM opinion dynamics can be generalized.
2. Narrow topical/measurement scope: A few topics on a five-point scale risk topic-specific effects and discretization artifacts.
3. Constrained interaction with potential reactivity: Short, dyadic dialogues (~5 rounds) limit extrapolation to longer horizons or multi-agent settings; per-round opinion probes (even if not persisted) may influence behavior.
4. Replication and validation gaps: ~25 runs per topic/initialization may be underpowered for stable estimation, and no human-grounded validation is presented.
5. Modeling, data, and reproducibility gaps: The linear additive specification may be restrictive with identifiability not fully demonstrated; missing extreme initializations reduce coverage; fine-tuning raises provenance/label-noise/ethics concerns; and runtime details (seeds, hardware, decoding) are insufficient for full replication.

(Remarks on code availability)

Reviewer #5

(Remarks to the Author)
I have now reviewed "Disentangling interaction and bias effects in opinion dynamics of large language models". This manuscript discusses different sources of bias in the behavior of LLMs when they are asked to simulate conversations between people who disagree. The authors decompose bias into three types, which they call topic, agreement, and anchoring biases. Looking at three different LLMs – DolphinMixtral, Mixtral-8X7B, and GPT-4o-mini – the authors use various measures to illustrate the role of these biases when using LLM as simulations of human conversations.

I found much to like about this manuscript – I liked the justification for the two different Mixtral models, the clarity of conceptualization around the different types of biases, and the work to understand how LLMs behave in these contexts. I think the technical results alone make the article have value for researchers working in this space.

At the same time, I had a set of questions that I thought the authors should address. These were the following:
(1) What is the target of inference for this study? In other words, I was hoping the authors would clarify a bit more what they would like to make scientific inferences about. Are they trying to determine how a class of LLMs performs this specific task? Do they want to know how faithful to human conversations a simulated conversation between two LLMs is? Something else? I wanted this element of the manuscript to be clearer from the start, especially because it has implications for how we should think of the results and what, if anything, is required to support the inferences the authors are pursuing.

(2) Related to this inference point, how stable do the authors feel that this type of behavior is? That is, does it seem to port across different domains (something political, something religious, etc.) or does the pattern rely on a specific context?

(3) One of the paper's main ideas is to document sources of bias in the behavior of LLMs. I wanted a bit more contextualization of these results and this bias – bias in comparison to what? Knowing the explicit or implied comparison point would help to determine the magnitude of the bias and the degree to which it should correctly be called bias.

(4) To what extent do the authors think that these kinds of dynamics in LLM behavior would carry through when the LLM discusses/interacts with a human? This is something to take up or think about in the discussion, rather than the main text.

(5) There were some technical details that I thought could have used a bit more explanation. For example, on line 112 on p. 3, the authors state that they simulated 25 discussions on three topics but also used five initial opinions per agent. I wanted

to know more about these decisions and to know what this implies about the full set of data that the authors are drawing from throughout the paper. Without more information on the data set, I have a hard time visualizing the size and composition of the analyses that go along with the figures and tables presented in the main text. Similarly, I thought the manuscript could provide some additional discussion of why they chose the three specific LLMs that they are working with in these analyses.

All in all, I found much to like and think that the author can make revisions that would satisfy my concerns.

(Remarks on code availability)
I briefly looked through the link provided. It seems to contain all of the necessary and required documents.

Version 1:

Reviewer comments:

Reviewer #2

(Remarks to the Author)
I thank the authors for their thorough revision. My point regarding the chain-of-thought initialization texts has been fully addressed. This addition improves the transparency of the study design, and I am happy to recommend acceptance.

(Remarks on code availability)

Reviewer #3

(Remarks to the Author)
I am happy with the revisions of the manuscript and would recommend its publication.

(Remarks on code availability)
I checked the code but am not able to make full assessment of the code.

Reviewer #5

(Remarks to the Author)
I have reviewed the revisions to this manuscript in response to my (and the other reviewers') comments on the initial submission.

In responding to those questions and comments, the authors have significantly expanded both the scope of the simulations (including more topics, for example) and the LLMs they are considering (adding three additional models of different types). This is an improvement to the project (even though I did not specifically ask for it or think it was required in all respects). I appreciated the efforts by the authors in this regard.

I found the responses to my questions and comments to be thoughtful and engaging in my core concerns. I would have appreciated more specific reference to places in the manuscript that were modified or revised in response to my comments; nonetheless, with only some effort, I was able to identify the parts of the text (and changes) to which the responses referred.

I especially appreciated the responses from the authors about their target of inference, and how the focus here is on "comparing biases and interaction effects in discussions between LLM agents", with only secondary future applications to human-human or human-LLM interactions (this is taken from their responses in the reviewer response memo). I also thought the way the authors removed any implicit comparisons or references to human interactions was an improvement to emphasize this focus.

My only lingering question in this regard - which I leave to the discretion of the editorial team - is if the authors can give some examples of places where these LLM to LLM interactions exist or are being used in some current setting. That would help to emphasize what, in the real world, the paper is speaking to.

Given other things the manuscript says about simulation and some future applications to human interactions, I do not think that this point should prevent the article from being published (although I do think it would be an improvement). As such, I am supportive of the acceptance of this manuscript.

(Remarks on code availability)
The code repository seems well-documented and constructed in a way that would be useable and understandable to others. I did not install and run the code myself.

Open Access This Peer Review File is licensed under a Creative Commons Attribution 4.0 International License, which permits use, sharing, adaptation, distribution and reproduction in any medium or format, as long as you give appropriate credit to the original author(s) and the source, provide a link to the Creative Commons license, and indicate if changes were made.
In cases where reviewers are anonymous, credit should be given to 'Anonymous Referee' and the source.
The images or other third party material in this Peer Review File are included in the article's Creative Commons license, unless indicated otherwise in a credit line to the material. If material is not included in the article's Creative Commons license and your intended use is not permitted by statutory regulation or exceeds the permitted use, you will need to obtain permission directly from the copyright holder.
To view a copy of this license, visit https://creativecommons.org/licenses/by/4.0/

Revision of our manuscript to *Nature Communications*

Dear Reviewers,

We are pleased to find that all *five* reviewers have found interest in our work and have provided constructive and overall positive feedback.

Following the suggestions, we have significantly expanded the scope of our work by increasing the number of discussion topics and LLMs, allowing us to extend our Bayesian model to include a hierarchical structure that accounts for topic-dependent differences. We furthermore provide extensive validation and robustness checks for the revised model. Finally, we added additional analyses and clarifications to the manuscript and the supplementary information.

Since some points have been raised by multiple reviewers, we first address these general points and additional major changes we made to our work in a general reply section, before responding to the individual items of the reviewers one by one.

In the revised manuscript, we marked changes in blue for your convenience.

We once again thank you for your valuable input and look forward to your reply,

Vincent Brockers, David Ehrlich and Viola Priesemann

General Replies

1) Topic and LLM scope

Following the suggestions from multiple reviewers, we have significantly expanded the empirical evidence for our claims by introducing both additional debate topics and LLMs.

For this revision, we increased the number of debate topics from three to twelve, grouped into four distinct topic categories: societal issues with scientific consensus, controversial societal issues, philosophical questions and matters of personal preference. We find that across LLMs, the topic bias attractors for the scientifically grounded questions cluster toward the extreme end of the scale matching the consensus opinion, while the personal preference topic bias attractors remain close to zero, with the other categories in between (see Fig. 6).

Additionally, we introduced three new LLMs: the locally hosted open-weight models Llama-3.1-8B and Qwen-2.5-7B have been added to investigate differences between smaller models of different architecture, training data and reinforcement learning, while Grok-4.1-fast has been included through API access to compare to another potentially more capable cloud-hosted LLM. Analysis of these models substantiates our previous finding of the topic bias dominating for the self-hosted models, with the interaction being more relevant for the cloud-hosted models (i.e., GPT-4o-mini and Grok-4.1-fast).

We furthermore added a new main text figure showing the degree of opinion convergence and the decay in opinion shifts after the first time step (see Fig. 3).

2) Bayesian model refinement

In the original formulation of our model, we noticed a collinearity between parameters that potentially precluded proper effect attribution between the agreement and anchoring biases in certain parameter ranges. To address this issue, our revised model uses a fixed agreement attractor position at the +2 (i.e., "strongly agree") value with a fitted effect strength only, instead of fitting the attractor position from the data as well. This resolves the collinearity problems, as demonstrated in the parameter recovery in Apx. F. See Fig. 2 for the full model.

To account for differences introduced by the distinct topics, we have refined our Bayesian model by adding a hierarchical formulation to the effect strengths, fitting a hyperparameter and topic-dependent variations for each (Fig. 2). Additionally, to analyze how the trade-offs between different biases and the interaction effect change over time, we added time decays to each of the bias terms. Following this extension, we have furthermore removed the contribution to the standard deviation proportional to the opinion shift, as we found it to no longer improve the model.

Two additional changes have been made to the model to improve numerical stability and convergence: the time variable t has been shifted so that $t = 0$ corresponds to the first time step, which reduces the correlation of the effect strengths with the decay. Furthermore, for fitting the model, the time decay τ has been replaced by a time decay constant $\lambda = 1/\tau$ to allow for a smooth transition from decay to increase. Nevertheless, we continue to discuss $\tau = 1/\lambda$ in the manuscript for its superior interpretability. These model choices are explained in detail in Apx. D.

3) Model validations

To improve the rigor of our findings, we included additional model validation and robustness checks, as outlined in the following:

To demonstrate the effectiveness of our models in capturing the explainable opinion shifts, we introduced a measure for the explained variance and compared it to the noise ceiling given by the inherent and non-explainable stochasticity in LLM discussion dynamics (see Fig. 7 and Apx. D). We observe that our full model captures between 32% and 77% of the variance depending on the LLM, with the cloud-hosted models being the most difficult to capture.

To validate the identifiability of parameters of our revised model, we added extensive parameter recovery studies to the appendix, showing how parameters in different realistic regimes of dominating effects can be faithfully recovered by our model (see Apx. F). Furthermore, we demonstrate that changes to the prompting and priors can affect numerical results, yet the major qualitative results remain stable (see Fig. 13).

Finally, we added two new figures showing the relation between the inferred topic bias attractor and the LLM's prior opinion about a statement without any system prompt, revealing high correlation of r between 0.62 and 0.96, with the exception of Grok with very low correlation (see Figs. 22 and 23).

4) Implementation changes Finally, we improved upon the implementation of our code, greatly improving runtimes as a required step to make the extended analyses possible. We changed from HuggingFace Transformers to a faster vLLM simulation pipeline, which required re-running the simulations for DolphinMixtral and Mixtral with AWQ quantization instead of bitsandbytes.

Reviewer 1

> This manuscript introduces a Bayesian framework to decompose opinion change in dyadic LLM debates into interaction and three biases (topic, agreement, anchoring), reporting rapid convergence to topic-dependent attractors, small sustained interaction effects, and an entropy–susceptibility link, plus a narrow fine-tuning case study.

We thank you for your helpful comments and suggestions, which led us to expand our manuscript, especially the controls and consistency checks. Below we address them point by point.

> **1.1** Major issue: The authors provide no validation that their method can recover known parameters from synthetic data. This is particularly concerning given evidence of identifiability problems in their results: GPT-4o-mini's agreement bias parameters are either zero-constrained ($\beta_{\text{agree}} = 0.0 \pm 0.0$) or completely unconstrained ($b_{\text{agree}} = 0.0 \pm 2.0$), some parameters show poor convergence, and the model structure creates severe collinearity when multiple bias terms pull toward similar attractors. Without demonstrating that the framework can correctly decompose effects when ground truth is known, the reader lacks sufficient evidence that could warrant their adoption of the proposed approach.

The agreement bias parameters for GPT-4o-mini were not at odds with each other: When no agreement bias is present in the data, the effect size parameter β_{agree} should be constrained to zero, as observed. In this case, the bias attractor parameter b_{agree} loses its influence on the prediction, correctly resulting in an unconstrained posterior.

Regarding the collinearity of bias terms, please note that the bias terms are generally applied differently to different data points (i.e., observed opinion shifts): While the topic bias is modeled proportional to the distance to the framing-sensitive topic bias location, the agreement bias relates to the same bias location irrespective of framing inversion. Finally, the anchoring bias is only applied to the second LLM agent with respect to $x_j(t = 0)$ Nevertheless, in the original formulation, effects from the agreement bias could be partially absorbed into the anchoring term and vice versa. We have solved this problem by fixing the agreement attractor position in our revised model (see Section 2 in the paper).

1.2 Standard practice for methods papers requires simulation studies showing parameter recovery across diverse scenarios (in this particular case: interaction-dominated, single-bias-dominated, competing biases). The authors should also test whether the framework incorrectly infers biases when applied to data generated with interaction alone, and explicitly characterize conditions under which decomposition is/isn't reliable.

We thank you for pointing out this clear path to making our analysis more robust and convincing. We have added a new appendix chapter with a comprehensive parameter recovery study, demonstrating that the revised Bayesian model successfully recovers parameters from different parameter regimes, including when effects are not present (see Apx. F).

1.3 Minor issues: 1. the modeling choices (one-sided prior ruling out negative agreement; fixed exponential decay with restrictive prior on the time constant; asymmetric anchoring applied only to the responder) narrow the hypothesis space in ways that can predetermine qualitative conclusions (e.g., "fast decisions"), yet sensitivity checks to alternative links/priors and order-balancing are limited.

While the agreement-bias effect strength β_{agree} was indeed chosen non-negatively, the original model could still capture negative agreement effects through the variable agreement-bias attractor position b_{agree}. In the revised model with fixed agreement-bias attractor position, we instead allow the effect strength to take both positive and negative values (see Section 2). Likewise, the anchoring bias is only applied to the responder, but can also take on negative values indicating a rejection of the initially stated opinion. Nevertheless, adding an additional term to model attraction or rejection to the responder's opinion might be a valid addition to the model that can be investigated in future work. We have added variations on the priors, including the decay constant (see Fig. 13), and found qualitatively equivalent results.

1.4 While the paper positions this work as relevant to "using LLMs as proxies for human behavior" (abstract), no comparison to human opinion dynamics data is provided. Without demonstrating that identified biases, effect sizes, and interaction timescales align with human studies, claims about human relevance remain unvalidated.

The goal of this paper is to introduce a novel quantitative framework for evaluating influence factors to opinion shifts in dyadic discussions between LLM agents, with potential future applications for modeling social dynamics or serving as proxies for humans in research settings. Comparing the results we find to human studies is an enticing next step for our research project, but out of scope for this manuscript. In the motivation for our fine-tuning experiments we have hypothesized differences between LLM and human subjects, particularly

that the interaction timescales appear unrealistic. To avoid confusion, we have carefully reformulated these statements and left a possible application to humans only as an outlook in the discussion.

> **1.5** Independent of the decomposition, several observations (biases dominating over interaction; quick convergence; entropy predicting susceptibility) are interesting but do not require the full framework and are already suggested by recent literature. The topical/model scope is narrow, robustness to decoding/prompt initialization and seeds is under-reported, and there is no human baseline to contextualize effect sizes or a downstream demonstration that the decomposition improves debate truthfulness or LLM-as-judge reliability.

We agree that several of the bias effects have been observed qualitatively in LLM agents before and have cited the appropriate literature in our work. What is novel, however, is a principled quantitative comparison of these effects, which we provide through our Bayesian modeling approach. We agree that the original scope has been limited and have added nine new topics and three new LLMs to our results, see general answer (1). Furthermore, we added an appendix chapter discussing robustness to prompt variations. While we do not report the particular seeds used for the LLM agents, we ensure the average results are reproducible by performing 25 differently seeded discussion runs for each pair of initial opinions, resulting in a total of 150,000 data points per LLM used for fitting the Bayesian models.

> **1.6** The work addresses an important question and the framework concept has merit for the emerging field of LLM agent-based modeling, but the provided evidence doesn't support the quantitative decomposition claims that constitute the paper's primary novelty.

We appreciate your acknowledgment of the relevance of our research question and the merit of our framework, and hope that you find our significantly expanded evidence in the form of more topics, LLMs and robustness checks persuasive.

Reviewer 2

This manuscript presents an original and carefully motivated contribution to the study of LLMs in interactive, multi-agent settings. By introducing a Bayesian framework to quantify and distinguish interaction effects from model-internal biases, the authors address an important and timely question at the intersection of AI and social behavior simulation. The approach offers a structured way to analyze how LLMs' opinions evolve in dialogue and to separate genuine influence from systematic biases such as topic alignment, agreement tendency, and anchoring. This focus makes the paper conceptually strong and relevant to ongoing discussions about the interpretability and behavior of generative language models.

We thank you for your assessment of the relevance and quality of our work.

2.1 The paper's overall structure and narrative are clear and well-motivated. The introduction provides a convincing rationale for why interpretability and decomposition of LLM-driven opinion dynamics are necessary, given the widespread use of such models as proxies for human-like discussion and debate. The integration of Bayesian methods adds coherence and interpretability to the analysis, offering a principled framework for comparing models and topics. The decision to evaluate several distinct LLMs and to demonstrate how each shows a characteristic bias profile strengthens the empirical contribution and enhances the general interest of the work.

To support our claims even more strongly, we have expanded the evidence of our claims by introducing more topics and LLMs, see general answer (1).

2.2 The work is both timely and relevant. As LLMs are increasingly used in simulations of dialogue, persuasion, and social interaction, developing rigorous ways to characterize their internal biases and influence patterns is an important step forward. The proposed framework provides a foundation for more systematic investigations of such phenomena and may inform both applied and theoretical research in AI and related disciplines. Overall, this paper represents a valuable and well-executed contribution to an emerging area of study.

We agree that the use of Bayesian modeling of (LLM) opinion dynamics opens the door to many promising future research avenues: First, interactions between more than two LLM agents in small simulated social settings may be studied to investigate novel contributions like peer pressure to the dynamics. Second, the Bayesian models may serve as proxies for the computationally expensive LLM agents, allowing to study the complex systems dynamics

of large networks such as opinion formation in simulated social media platforms. Third, comparisons to human-LLM or human-human dialogues promise to provide valuable insights into the similarities and differences between artificial and human persuasion and opinion formation, with possible ramifications to the understanding of online political debates of humans with (undisclosed) bots.

2.3 A minor point that could be clarified concerns the role of the short “chain-of-thought” texts used to initialize each agent. These texts appear to establish the initial reasoning and stance from which the agents begin their discussions, yet the manuscript does not present or analyze them in detail. Because these texts effectively shape how each agent interprets and frames the topic, variation in their content or tone could influence the agents’ subsequent responses and the overall opinion shifts observed. Providing a few illustrative examples or a brief examination of their consistency would help readers better understand how the initialization process might contribute to the reported effects and strengthen the transparency of the study’s design.

We thank you for the suggestion to add samples of chain-of-thought texts, and have added them in a new Appendix section (Apx. C). We believe that this addition creates valuable insights into cases where agent initialization succeeds, but also where the agents fail to adopt the prompted initial opinion.

Reviewer 3

This manuscript examines opinion dynamics among LLM agents through a Bayesian modeling framework, and disentangle the relative contribution of the effect of interaction between agents and three intrinsic biases – topic, agreement and anchoring – during simulated discussions. Overall, the research is well conducted, with clearly documented procedure and analysis. It contributes to the literature by quantifying the effects through a mathematical framework. Below are some comments for authors to consider when revising this manuscript.

We thank you for your feedback and have addressed your points one by one below.

3.1 Understand that the interaction effect represents an ongoing social influence between two agents who are currently disagreeing, while the anchoring bias applies to the second agent when exposed to the first agent's initial opinion. However, the anchoring bias could be seen as a special case (first round) of an interaction effect when the second agent first joins the conversation, as both lie on the continuum of social influence mechanisms, differing primarily in timing and symmetry. Re-consider these two concepts or a brief clarification of this relationship would help strengthen theoretical coherence.

While it is true that the anchoring bias can be considered a social influence mechanism, we believe that the differentiation between the symmetric influence term and the asymmetric anchoring bias sheds valuable light onto the relevance of the order of responses between the agents. Mathematically, the two effects are distinct and can be faithfully reconstructed from synthetic data (see new appendix chapter on parameter recovery). While the anchoring bias is only one example of an asymmetric interaction effect, it is well-described in the literature and should be considered a starting point to assess more complex asymmetric interaction rules in the future. This relevance can subsequently be compared in direction and strength to anchoring biases in human subjects, providing a basis for future comparisons between asymmetric effects in LLM-agent, human-LLM, and human-human interactions.

3.2 The results differ across topics, but the implications of these variations are not fully discussed. In addition, since only one topic (climate change) was used for fine-tuning, the authors may want to clarify whether the observed changes are topic-specific or reflect generalizable tendencies.

In our revision, we have significantly increased the topic scope from three to twelve topics from four distinct topic categories. For societal questions with scientific consensus, we find that the topic bias positions are closer to the limits of the opinion range and in agreement with the consensus opinion, while they remain closer to zero for topics of personal preference (see Fig. 6).

The shifting topic attractor positions for the fine-tuned model on climate change as a case study furthermore provides interventional evidence for the topic bias position correctly accounting for the prior opinion encoded in the LLM. Investigating the impact of fine-tuning on more LLMs and topics is an important future research direction, but out of the scope for this manuscript.

> **3.3** The description of the simulation procedure could specify how stochasticity is introduced in the Chain-of-Thought prompts and whether each run is seeded differently.

The stochasticity in Chain-of-Thought prompts is introduced by the non-zero temperature of the LLMs, which have been randomly seeded for each run, resulting in diverse Chain-of-Thought outcomes even for the same topic and opinion value. While we do not report the particular seeds used for the LLM agents, we ensure the average results are reproducible by performing 25 differently seeded discussion runs for each pair of initial opinions, resulting in a total of 150,000 data points per LLM used for fitting the Bayesian models. We added illustrative examples for Chain-of-Thought prompts both for successful (i.e., probed opinion closely matches the prescribed opinion) and unsuccessful initialization to Apx. C.

> **3.4** The term “topic bias shift” may be a bit misleading in conceptual terms. It intends to reflect a pulling force from an assigned role back toward the model’s default stance, regardless of whether that default arises from data-driven bias. In other words, as long as the assigned role is different from the default opinion, the pulling effect will occur. Thus, the focus here is not the topic bias, but rather the influence of the prior stance. Given this, the authors could consider using a more neutral term such as “default effect” or “topic prior effect”, or add a note justifying the choice of “Topic Bias” over a neutral term.

The goal of our approach is to assess the usefulness of LLM-based agents in modeling opinion dynamics between agents with different truly held opinions. We show in our work that generating LLM agents that truly hold an opinion through prompting can be insufficient at times, as prompting alone fails to fully override the prior opinions encoded in the data. Since this pull towards the prior opinions is an undesirable effect if the LLMs are used to model truly opinionated agents (as opposed to agents roleplaying or playing “devil’s advocate”), we

term this contribution a “bias”, i.e., a systematic deviation from the expected outcome, and suggest strategies to mitigate it through fine-tuning.

3.5 The use of an exponential decay function for interaction influence is standard in opinion dynamics models. However, the paper itself notes that the inferred decay is “unrealistically fast” and “unrealistically low” compared to humans (p6), and that fine-tuning yielded only modest improvement. It would be valuable to discuss the implications of this rapid decay. For instance, whether it limits the model’s comparability to human data, or whether the effect is intrinsic to LLM interaction patterns rather than a methodological artifact.

So far, the comparison to humans is conjecture and we have rephrased the motivation paragraph for the fine-tuning section in our paper to communicate this more clearly. However, we agree that a comparison to real human data is a promising new research direction which we leave to future work.

3.6 Mixtral-8x7b and GPT-4o-mini exhibit a small negative anchoring effect, which is counter-intuitive. A short discussion of this finding would be helpful. Does it indicate a contrastive or rejection bias against the first-stated opinion, or might it result from constraints in the model fitting?

In the revised model, the anchoring effect close to zero or negative for all LLMs. While this indeed implies a rejection of the first-stated opinion, which might be interpreted as an initial stubborn refusal of opposing opinions, please note that the ablation studies reveal little explanatory power of this effect by itself. Future work experimenting with different variations of asymmetric interaction effects and comparisons to human subjects are likely warranted to give a more conclusive account of this empirical finding, which we will leave for future work.

3.7 A few minor comments: Figure 5 (p. 7): Remove the duplicated “the” in the third-to-last line of the caption. Tables: Ensure consistent use of decimal places across parameter estimates.

We thank you for your detailed feedback and have corrected these issues.

Reviewer 4

This is a thoughtful, well-structured study with a clear methodological contribution and commendable transparency. Addressing the points below, especially scope, measurement, and posterior diagnostics, would materially strengthen the work.

Thank you for your meticulous review. We have addressed your individual points one by one in the following.

4.1 Five main disadvantages: 1. Limited external validity: Findings are drawn from a small set of models, constraining how broad conclusions about LLM opinion dynamics can be generalized.

We agree that the number of models was too constrained and have added three new LLMs to our analysis, two smaller locally-hosted LLMs and one additional more state-of-the-art cloud-hosted LLM (see general response 1).

4.2 2. Narrow topical/measurement scope: A few topics on a five-point scale risk topic-specific effects and discretization artifacts.

Similarly, we agree that the choice of topics was too restrictive. We have extended it in our revised manuscript to twelve topics from four distinct topic categories (see general response 1).

Please note that we already employ several mechanisms to avoid discretization effects: For probing, we use the continuous expectation value of the expressed opinion values instead of just the most likely answer token, providing finer resolution of the LLM agents opinion state. Likewise, for initialization, we first have the agents generate internal Chain-of-thought monologues and prompt their opinion expectation value directly afterwards, providing additional continuous variability on the continuous agreement scale.

4.3 3. Constrained interaction with potential reactivity: Short, dyadic dialogues (5 rounds) limit extrapolation to longer horizons or multi-agent settings; per-round opinion probes (even if not persisted) may influence behavior.

While the dialogues may appear short, we find that the opinion dynamics typically converge quickly with only minor changes after only very few rounds, precluding the necessity for simulating longer sequences, which would drastically increase the required compute. Additionally, in internal experiments, we tested conversations with twice the amount of time steps and saw no qualitative differences.

Whether our models account for the dynamics in multi-agent settings or whether additional influence factors, such as peer pressure, need to be modeled explicitly, remains an interesting question for future research but is beyond the scope of our current inquiry.

Both the prompts and the answered tokens for the opinion probing are completely removed from the LLMs context before discussion continues, making it impossible for them to influence LLM behavior in later steps.

4.4 4. Replication and validation gaps: 25 runs per topic/initialization may be underpowered for stable estimation, and no human-grounded validation is presented.

In the appendix of the revised manuscript, we show that increasing the number of runs from 25 to 50 per topic and initialization does not change the results of the Bayesian analysis on synthetic data meaningfully (see Fig. 13). We therefore refrained from running additional iterations due to computational costs.

Validating our Bayesian models using data from human interactions is a promising future research direction, but out of scope for this manuscript.

4.5 5. Modeling, data, and reproducibility gaps: The linear additive specification may be restrictive with identifiability not fully demonstrated; missing extreme initializations reduce coverage; fine-tuning raises provenance/label-noise/ethics concerns; and runtime details (seeds, hardware, decoding) are insufficient for full replication.

We thank you for these suggestions to improve the robustness of our claims. In our new manuscript, we present an extensive parameter recovery study showing that the Bayesian model faithfully recovers parameters in different parameter regimes (see Apx. F).

For the fine-tuning study, please note that it is meant as a case study demonstrating a possible future research direction. The labels have been generated using GPT-4o-mini, potentially weakening our results due to misclassification of statements. To address your ethics concerns, we confirm that the data has been obtained only from public Telegram channels with >10,000 users and has been stripped of any usernames or other possible identification of individual persons. To ensure reproducibility of our results, we have made the fine-tuning checkpoints available from our github repository.

Reviewer 5

I have now reviewed "Disentangling interaction and bias effects in opinion dynamics of large language models". This manuscript discusses different sources of bias in the behavior of LLMs when they are asked to simulate conversations between people who disagree. The authors decompose bias into three types, which they call topic, agreement, and anchoring biases. Looking at three different LLMs – DolphinMixtral, Mixtral-8X7B, and GPT-4o-mini – the authors use various measures to illustrate the role of these biases when using LLM as simulations of human conversations.

I found much to like about this manuscript – I liked the justification for the two different Mixtral models, the clarity of conceptualization around the different types of biases, and the work to understand how LLMs behave in these contexts. I think the technical results alone make the article have value for researchers working in this space.

We thank you for your review and address all raised concerns in the following sections.

5.1 At the same time, I had a set of questions that I thought the authors should address. These were the following: (1) What is the target of inference for this study? In other words, I was hoping the authors would clarify a bit more what they would like to make scientific inferences about. Are they trying to determine how a class of LLMs performs this specific task? Do they want to know how faithful to human conversations a simulated conversation between two LLMs is? Something else? I wanted this element of the manuscript to be clearer from the start, especially because it has implications for how we should think of the results and what, if anything, is required to support the inferences the authors are pursuing.

The main contribution of this work is to provide a quantitative and modular framework that allows for comparing biases and interaction effects in discussions between LLM agents. We demonstrate that LLM agents are mostly driven by a topic bias and interaction effects, and show differences between LLM classes. For future work, the models may be compared to human-human or human-llm interactions, and can serve as computationally inexpensive proxies for LLM agents in large-scale social simulations.

5.2 (2) Related to this inference point, how stable do the authors feel that this type of behavior is? That is, does it seem to port across different domains (something political, something religious, etc.) or does the pattern rely on a specific context?

We thank you for this clear suggestion to improve our paper. We have added nine new topics, for a total of twelve topics from four distinct topic categories to our new manuscript, highlighting relevant differences in their topic-related biases (see general response 1).

> **5.3** (3) One of the paper's main ideas is to document sources of bias in the behavior of LLMs. I wanted a bit more contextualization of these results and this bias – bias in comparison to what? Knowing the explicit or implied comparison point would help to determine the magnitude of the bias and the degree to which it should correctly be called bias.

In this manuscript, we use the term "bias" broadly to denote any deviation from purely interaction-driven dynamics. Our work evaluates the suitability of LLM-based agents for modeling opinion dynamics among agents with genuinely distinct opinions. We demonstrate that prompting alone is sometimes insufficient to instantiate truly opinionated LLM agents, as it cannot fully override the prior opinions encoded during training. This residual pull toward prior opinions is problematic when LLMs are intended to represent genuinely opinionated agents, rather than agents roleplaying or arguing devil's advocate positions. We therefore frame this phenomenon as a "bias"—a systematic deviation from the expected outcome—and propose fine-tuning strategies to mitigate it.

> **5.4** (4) To what extent do the authors think that these kinds of dynamics in LLM behavior would carry through when the LLM discusses/interacts with a human? This is something to take up or think about in the discussion, rather than the main text.

We believe that the generality of the Bayesian approach invites comparisons to human data. This, however, remains out of the scope for this current manuscript. Where we had conjectured comparisons to humans in the original manuscript, we have carefully removed the reference to suspected human behavior and more clearly present this only as outlook in the discussion.

> **5.5** (5) There were some technical details that I thought could have used a bit more explanation. For example, on line 112 on p. 3, the authors state that they simulated 25 discussions on three topics but also used five initial opinions per agent. I wanted to know more about these decisions and to know what this implies about the full set of data that the authors are drawing from throughout the paper. Without more information on the data set, I have a hard time visualizing the size and composition of the analyses that go along with the figures and tables presented in the main text. Similarly, I thought the manuscript could provide some additional discussion of why they chose the three specific LLMs that they are working with in these analyses.

Thank you for this suggestion. In the new manuscript, we have increased the number of topics to 12 from four distinct topic categories, to capture a broader sample of possible questions. Furthermore, we have included three additional LLMs, to better cover the span from smaller, locally-hosted models to more recent, closed-source and cloud-hosted LLMs.

To ensure statistical reliability of our results, our bayesian models are trained on $150,000$ datapoints per LLM, resulting from 25 repetitions for each of the 12 topics, 2 framings, 5x5 initial opinion pairs, 2 agents and 5 timesteps.

Our selection of LLMs was made to cover the space of models with different architectures, datasets and fine-tuning, by using four locally-hosted open-weight models (Mixtral, Dolphin-Mixtral, Llama and Qwen), and two more state-of-the-art, closed-source and cloud-hosted models (GPT and Grok).

> All in all, I found much to like and think that the author can make revisions that would satisfy my concerns. (Remarks on code availability): I briefly looked through the link provided. It seems to contain all of the necessary and required documents.

We thank you for your feedback and hope we could address all of your remaining points in our revision.

Reviewer 5

> My only lingering question in this regard - which I leave to the discretion of the editorial team - is if the authors can give some examples of places where these LLM to LLM interactions exist or are being used in some current setting. That would help to emphasize what, in the real world, the paper is speaking to.
> Given other things the manuscript says about simulation and some future applications to human interactions, I do not think that this point should prevent the article from being published (although I do think it would be an improvement). As such, I am supportive of the acceptance of this manuscript.

We thank the reviewer for this suggestion and have added the following paragraph to the introduction section:

Beyond simulation studies, direct interactions between LLM agents are rapidly becoming a feature of deployed systems: autonomous agents now coordinate within multi-agent software frameworks [1, 2], negotiate contracts with one another in commercial legal workflows [3], converse and form opinion-laden discourse communities on agent-only social platforms such as Moltbook [4], and debate to improve factuality and enable scalable oversight [5–7]. In all of these settings, whether an agent's response is driven by its interlocutor's arguments or by model-intrinsic biases directly affects outcomes, underscoring the need for quantitative tools to disentangle these factors.

*

References

1. Wu, Q. *et al. AutoGen: Enabling Next-Gen LLM Applications via Multi-Agent Conversation* 2023. arXiv: 2308.08155 [cs.AI]. https://arxiv.org/abs/2308.08155.
2. Li, G., Hammoud, H. A. A. K., Itani, H., Khizbullin, D. & Ghanem, B. *CAMEL: Communicative Agents for "Mind" Exploration of Large Language Model Society* in *Advances in Neural Information Processing Systems* **36** (2023), 51991–52008.
3. CNBC. *An AI just negotiated a contract for the first time ever — and no human was involved* https://www.cnbc.com/2023/11/07/ai-negotiates-legal-contract-without-humans-involved-for-first-time.html. Accessed: 2026-07-23. 2023.
4. Jiang, Y., Zhang, Y., Shen, X., Backes, M. & Zhang, Y. *"Humans welcome to observe": A First Look at the Agent Social Network Moltbook* 2026. arXiv: 2602.10127 [cs.SI]. https://arxiv.org/abs/2602.10127.

5. Du, Y., Li, S., Torralba, A., Tenenbaum, J. B. & Mordatch, I. *Improving Factuality and Reasoning in Language Models through Multiagent Debate* in *Proceedings of the 41st International Conference on Machine Learning* **235.** arXiv:2305.14325 (PMLR, 2024).

6. Khan, A. *et al. Debating with More Persuasive LLMs Leads to More Truthful Answers* in *Proceedings of the 41st International Conference on Machine Learning* **235.** arXiv:2402.06782 (PMLR, 2024).

7. Irving, G., Christiano, P. & Amodei, D. *AI Safety via Debate* 2018. arXiv: `1805.00899 [stat.ML]`. `https://arxiv.org/abs/1805.00899`.
